# Supplementary material for: Health systems analysis and evaluation of the barriers to availability, utilisation and readiness of sexual and reproductive health services in COVID-19-affected areas: a WHO mixed-methods study protocol
Source: BMJ Open. 2022 Jun 1;12(6):e057810. doi: 10.1136/bmjopen-2021-057810 (PMC9160592; doi:10.1136/bmjopen-2021-057810)
Supplement: Supplementary data [file bmjopen-2021-057810supp001.pdf]

Focused group topic guide for women

Demographic information (Note: Collect data on individuals before the FGD)

- Age (in completed years)
- Education (highest level attained)
- Residence (actual, urban, rural, slum)
- COVID-19 infection history (self, close family, work mates, friends)
- Social economic status - Employment status (i.e. formal or informal employment), income earning status and possibly income ranges as suitable in the country setting.

Knowledge about COVID-19 (such as cause, signs and symptoms)

1. What is COVID-19? What do you know about COVID-19?
   PROBE: for causes, transmission, signs, symptoms, sources of information about COVID-19
2. Did you perceive yourself to be at risk for COVID-19 infection?
   PROMPT: Probe for any special considerations (pregnancy, pre-existing health conditions, others).

Care seeking and perception of risk about being infected by COVID-19

3. The decision-making process of treatment seeking during COVID-19
   PROBE: Ask who influenced the decision to seek care during COVID-19 (self, partner, other family member, etc?)
4. Perception of risk of infection during SRH care seeking.
   PROMPT: How did this affect health seeking behaviour during COVID-19?
5. The treatment-seeking process during COVID-19
   a. Were your needs for accurate information on reproductive health services fulfilled? How? Or Why not? PROBE: information sources: brochures, social media, billboards, other printed media, information on service hours or location where services are offered etc.
   b. What were your needs for reproductive health services? Were these needs fulfilled?
      PROBE: Contraception? Abortion care? STI?
   c. Did you encounter any barriers in your bid to access needed reproductive health services?
      PROBES: transportation, behaviour of other persons, etc.

   PROMPT: Interviewer should probe for other context specific intermediaries to seeking care. E.g. paperwork for insurance
6. Do you think that reproductive services were readily available to clients who needed them? Specifically, the availability of the reproductive health medicine and medical supplies needed by clients based on the following:
   a. Availability of staff, medicines and supplies for SRH during COVID-19
   b. Schedule of treatments/appointments during COVID-19
      PROMPT: Probe for changes in the delivery of SRH services (specifically for family planning, abortion, post-abortion care and STI care) i.e. appointment-based system introduced.
   c. Interaction between the service provider and the patient was during COVID-19. PROBE: Interactions with different cadres of health workers. Nurses? Midwives? Doctors? Records? OPD staff? Etc.
   d. Was the interaction between service providers and staff described above different from pre-COVID-19 time? How?

7. What were some of the safety measures ensured by the health facility to protect clients from COVID-19 during services
8. Do you think that the COVID-19 had any impact on client protection and privacy during services?
   PROBE: What was done (or could have been) differently?

Post-pandemic recovery of the facilities to provide services in comparison to the pandemic period.

GENERAL COMMENTS: Based on the country situation, the probes for the questions above can be extended to cover the post-pandemic period if applicable.
