# Supplementary material for: Health systems analysis and evaluation of the barriers to availability, utilisation and readiness of sexual and reproductive health services in COVID-19-affected areas: a WHO mixed-methods study protocol
Source: BMJ Open. 2022 Jun 1;12(6):e057810. doi: 10.1136/bmjopen-2021-057810 (PMC9160592; doi:10.1136/bmjopen-2021-057810)
Supplement: Supplementary data [file bmjopen-2021-057810supp002.pdf]

Focused group topic guide for men

Demographic information

- Age (in completed years)
- Education (highest level attained)
- Residence (actual, urban, rural, slum)
- COVID-19 infection history (self, close family, work mates, friends)
- Social economic status: Employment status (i.e. formal or informal employment), income earning status and possibly income ranges as suitable in the country setting.

Knowledge about COVID-19 (such as cause, signs and symptoms)

1. What is COVID-19? What do you know about COVID-19?
   PROBE: for causes, transmission, signs, symptoms, sources of information about COVID-19
2. Did you perceive yourself to be at risk for COVID-19 infection?
   PROMPT: Probe for any special considerations (pregnancy, pre-existing health conditions, others).

Care seeking and perception of risk about being infected by COVID-19

1. What role did you play in the decision-making process of treatment seeking during COVID-19?
   PROBE: final decision maker (man only, joint, woman only, other); stage of involvement (decision stage or care seeking stage).
2. The role of men in the treatment-seeking process during COVID-19
   a. What role did you play in seeking for accurate information on reproductive health services? Were your needs fulfilled? How? Or Why not?
      PROBE: information sources: brochures, social media, billboards, other printed media, etc. The role of men in looking for sexual reproductive health services (including contraception and abortion care) and whether the needs were fulfilled
   b. What role did you play in looking for reproductive health services? Were your needs for reproductive health services fulfilled?
      PROBE: Contraception? Abortion care? STI?
   c. Did you encounter any barriers in your bid to access needed reproductive health services? PROBES: transportation, behaviour of other persons, entry restrictions for men, etc.
      NOTE: Interviewer should probe for other context specific intermediaries to seeking care. E.g. paperwork for insurance
3. Do you think that reproductive services were readily available to clients who needed them? The availability of the reproductive health medicine and medical supplies needed by clients?
   a. The schedule of treatments/appointments during COVID-19?
4. How was the interaction between the service provider and you as the male partner during COVID-19?
   PROBE: Interactions with different cadres of health workers. Nurses? Midwives? Doctors? Records? OPD staff? Etc
5. Was the interaction between service providers and staff described above different from pre-COVID-19 time? How?

6. What were some of the safety measures ensured by the health facility to protect clients from COVID-19 during services?
   PROBE: Were male partners stopped from entering consulting and treatment rooms due to COVID-19?
7. Do you think that the COVID-19 had any impact on client protection and privacy during services?
8. **PROBE**: What was done (or could have been) differently? E.g. barriers in seeking SRH services during COVID

Post-pandemic recovery of the facilities to provide services in comparison to the pandemic period.

COMMENT: Based on the country situation, the probes for the questions above can be extended to cover the post-pandemic period if applicable.
