# Supplementary material for: Health systems analysis and evaluation of the barriers to availability, utilisation and readiness of sexual and reproductive health services in COVID-19-affected areas: a WHO mixed-methods study protocol
Source: BMJ Open. 2022 Jun 1;12(6):e057810. doi: 10.1136/bmjopen-2021-057810 (PMC9160592; doi:10.1136/bmjopen-2021-057810)
Supplement: Supplementary data [file bmjopen-2021-057810supp004.pdf]

In-depth Interview topic guide for men

1. Demographic information
    - Age
    - Ethnicity
    - Marital status
    - Socio-economic status
    - Occupation
    - Education status
    - Religion
    - Place of residence and housing condition

2. Knowledge about COVID-19
    - Cause (sexual transmission)
        - How do you think COVID-19 virus is transmitted?
        - Do you think it can be transmitted sexually?
    - Signs and symptoms of COVID-19
        - What are the sign and symptoms of COVID-19?
        - If yes, can you mention some that you know?
    - Care-seeking for COVID-19 infection
        - Do you know what to do/who to contact/ where to go if you experience COVID-19 signs and systems?
        - If yes, can you explain?
    - Sources of information and trusted communication channels (v. rumors, misinformation) Experience of COVID-19 (direct / indirect)
        - Where did you get information about COVID-19?
        - Was the information you obtained accurate?
        - Can you give me an example of accurate information you obtained?
        - Were there any rumors and/or misinformation about COVID-19?
        - How did you know that this information was just rumors or misinformation?
        - Have you fund sources of information that you can trust?
        - How did you know which information sources to trust?
        - Have you been infected by COVID-19?
        - Do you know of anyone who has been infected? If yes, was it a close friend or family member?
    - Perception of risk about being infected by COVID-19
        - Do you think you are at risk of being infected by the COVID-19 virus?
        - If no, can you tell me why not?
        - Do you think you can be infected whilst seeking care?
    - Ways to prevent being infected by COVID-19 (probe at individual, household, community levels)
        - Do you know how to prevent infection from COVID-19?
        - What can individuals do? What can households do? What can communities do?
    - Responsibility to prevent COVID-19 (probe at individual, household, community levels, authorities)
        - Who is responsible for the prevention of COVID-19 infection? Do you think this responsibility was fulfilled by individuals, households, communities and

authorities? How?

- Religious / socio-cultural practices and influencers
  - Are there any religious / socio-cultural practices that are used to prevent COVID-19 infection?
  - Does participation in any religious / socio-cultural practices make you practice prevention of COVID-19 differently from what is promoted by health professionals?

3. Sexual and Reproductive Health
   - Decision-making and agency to act
     - Who makes the decisions related to the sexual and reproductive health needs of your partner (you, partner or family member)?
     - Who is usually responsible for carrying out the decision?
   - Issues of access / utilization
     - Did your partner need to seek SRH services during COVID-19?
     - If yes, was your partner able to access the services? How did your partner access the services?
     - If no, what were the barriers? What did you and/or your partner do?
     - What role did you play in ensuring your partner sought and/or accessed the needed SRH services?
   - Challenges faced
     - Are there any other challenges your partner faced in seeking SRH services during COVID-19? What are they and how did you and/or your partner address/manage the challenges?

4. Contraceptive practices
   - Changes in practice (if any) due to COVID-19
     - Which method of contraception do you and/or your partner normally use?
     - Did you have to change your normal method of contraception during COVID-19? If yes, which method and what influenced the change?
     - If no, why didn't you change from your normal practice?
   - Did your partner avoid / delay pregnancy due to COVID-19? If yes, why?
   - Access to / utilization of different methods of contraception (barriers / drivers)
   - Which methods of contraception were accessible to you and your partner during COVID-19?
   - Was there a difference in the methods of contraception accessible to you compared to before COVID-19?
   - What was causing the difference?
   - Challenges in access / utilization
   - Are there any other challenges you and your partner faced in accessing/utilizing contraception services during COVID-19?
   - If yes, what were these challenges and how did you cope with them?
   - What role did you play in ensuring your partner sought and/or accessed contraception services?

5. Induced abortion

- Perceptions about abortion at normal times
- The role abortion plays in women's lives
  - Why do you think women seek abortion services?
  - Do you think abortion is an essential service for women? If yes, in what way?
- Fear of stigma
  - How does family, community and society view women who seek or have sought abortion services?
  - Does this stigma affect your decision whether to have an abortion?
  - Does this stigma affect how you will seek abortion services? If yes, how?
  - What do you think are the effects of this stigma on women who need abortion services?
- Inequities in access to safe abortion
  - Do you think safe abortion services are equally accessible to all women who need them?

- Perceptions about abortion during COVID-19
  - Would you choose to seek the abortion services during COVID-19, if your partner needed one?
  - If no, why not? If yes, where and how would you access the service?
- Access to different methods of abortion (barriers / drivers)
  - Did your partner seek abortion services during COVID-19? If yes, which abortion methods were available (medical abortion or surgical abortion)?
  - What pushed you and your partner to have an abortion? If you were given a choice of methods, which one did you choose and why?
  - Were there any barriers in seeking and accessing abortion services during COVID-19?
- Challenges in accessing / utilizing safe abortion services (inequities in access)
  - Are there any other challenges you faced in accessing/utilizing safe abortion services? If yes, what were the challenges and how did you address them?
- What was your role in ensuring your partner accessed the abortion service?

6. Violence against women (*Interviewer should use the term that is used locally to describe VAW)*
   - Formal and informal systems of support, and coping strategies for VAW
     - Has your partner or any woman you know experienced any form of VAW?
     - What are some of the systems of support available for women who have experienced VAW?
     - Do you know where and how to access this support?
     - What are some of the coping strategies that women who have experienced such violence use?
   - Perceptions of safety in home and in community safety/increased risk during COVID-19
     - Do you think women are safer or at increased risk of experiencing VAW at home or in the community during COVID-19?
     - What has made it safer/riskier for women during COVID-19?
   - Women's expectations of services for gender-based violence

- If your partner experiences GBV during COVID-19, do you expect the care and support services to be available?
- Do you know where and how to access these services?
- Do you expect any barriers to accessing these services during COVID-19? If yes, what kind of barriers?
- What do you perceive as your role in ensuring your partner get the care and support needed?
- What are additional stress factors (e.g. school-age children at home, older parents, loss of job/income, partner loss of job, etc.) that increase the likelihood of VAW?
- How does stress affect frequency and severity of violence, particularly during COVID-19?

6. Sexually transmitted infections *(interviewer should use the locally known terms for STIs and the specific diseases)*

- Perceptions about STI
  - Is anybody at risk of getting a STI? Why or why not?
  - How are people who are known to have a STI perceived in the community?
- Experience of STI during COVID-19
  - Has your partner required care and treatment for a STI during COVID-19? If yes, where and how did you seek this service?
  - Were there any barriers to accessing this service? If yes, which ones and what did you do?
  - If no, would you seek care and treatment if required during COVID-19?
  - If no, why not? If yes, where and how would you access this service?
  - What was your role in ensuring your partner accessed the care and treatment?

7. Psycho-social
   - Psychosocial issues associated with being infected with COVID-19 (probe: more significant for women/men, more significant for pregnant women)
     - Did you experience any stress, anxiety or depression during COVID-19? If yes, at what level (mild, moderate, severe)?
     - What do you think led to the psychosocial issues you experienced? Was it due to being infected or other related issues or both?
     - Did your partner also experience any psychosocial issues during COVID-19?
     - Who was most affected, yourself or your partner?

8. Final questions
   - On-going concerns about COVID-19 and its possible impact
     - Are there any other concerns about COVID-19 and its impact on you, your family or in general that you wish to express?
   - Needs related to accurate information (probe further information needs – NB consider how to provide information if necessary)
     - Was accurate information readily available and accessible during COVID-19?
     - Was it easy to understand?
     - Was the information that was available adequate?
     - Was there additional information you would have liked to obtain but was

missing?
- Are there better ways of making accurate information accessible to you and others?

- Needs related to sexual reproductive health services (probe further SRH needs – NB consider how to support/provide if necessary)
- Do you think SRH services were responsive to your needs during COVID-19?
- If no, which of your needs were not met?
- Which SRH services are most important for you, that should be available even in health emergencies like COVID-19?
- How do you think provision of SRH services can be supported during other health emergencies like COVID-19?

Additional themes will be added as appropriate for each target group and questions revised accordingly (with attention to first person / third person views), and across the three phases of the study.
