# Supplementary material for: Health systems analysis and evaluation of the barriers to availability, utilisation and readiness of sexual and reproductive health services in COVID-19-affected areas: a WHO mixed-methods study protocol
Source: BMJ Open. 2022 Jun 1;12(6):e057810. doi: 10.1136/bmjopen-2021-057810 (PMC9160592; doi:10.1136/bmjopen-2021-057810)
Supplement: Supplementary data [file bmjopen-2021-057810supp005.pdf]

In-depth Interview topic guide for SRH healthcare providers

Demographic information

- Age
- Ethnicity
- Marital status
- Socio-economic status
- Occupation
- Education status
- Religion

Health facility service availability and readiness

Health service delivery

1. Mobilization of resources for the diagnosis and the treatment of COVID-19 patients by the *(insert Country name*) government happened during COVID-19. Did the mobilization involve your facility? How was the facility involved? (Probe community, regional or national level involvement)

   COMMENT: *Indicate if facility was a designated COVID-19 treatment Centre*
2. What was the emergency response plan of your facility during the outbreak?
3. How did the mobilization of resources towards COVID-19 patients impact the service delivery in your facility, including the impact on human resources, space, medical supplies, etc.?
4. Did the mobilization of resources negatively or positively impact service delivery in your facility? Elaborate
5. Was there a set of essential Sexual & Reproductive Health services outlined to be maintained during the lockdown phase of the COVID-19 pandemic?
   PROMPT: Ask for each of the following services – family planning, abortion, post-abortion care, care and support for GBV and STI prevention and treatment
6. Were there disruptions to any of the SRH essential services outlined? What was the nature (services suspended, limited or shifted) of disruptions observed for each of these essential services (if any)?
   PROBES: for causes (lack of personnel, equipment, medicines or reduced demand, physical space), changes in usual service provision (introduction of appointment-based systems, telemedicine, shifting of usual location) and level of disruption (partial/ complete/not disrupted)
7. Do you think the delivery of SRH services during COVID-19 adequately responded to the needs of the clients at that time?
   PROBE: in what way?
8. What has the recovery process been like and what was the turning point?
   NOTE: Only for applicable countries.
   PROBE for coping mechanisms or approaches to ensuring SRH service delivery.

Health workforce （Human resources）

9. How did COVID-19 affect the health status of health workforce, such as doctors, nurses, ambulance drivers and hospital managers etc.? What measures were taken to protect them from the effects of the epidemic?

PROBE: for the provision of PPE, type of PPE, adequacy of supply and frequency of supply.

10. Was there any form of training on COVID-19? PROMPT: Probe for details of the training if provided – What areas of COVID-19? What form did this training take? Who received this training?
11. Were there employees infected? If someone was infected, how did the infection happen? What was the impact on service delivery due to the infection of SRH workers? How did it influence the service delivery?

COMMENT*: In facilities where SRH staff also provide general care in health facility, ask for the impact of an infected clinical staff in the facility even if not SRH-specific*

12. While knowing that they were at higher risk of contracting the virus, did female workers still have to fulfil the obligations such as caring for elders and immediate family (such as children or young dependents)? Is there any other conflict or challenge they faced during COVID-19? Note: Probe for any challenge worth considering in current setting.

Health information system

13. How does the reporting system for infectious diseases run?
PROMPT: Probe for reporting system for COVID-19.
14. How did this system influence the availability of services in SRH facilities?
15. How did you keep information about the COVID-19 and SRH services flowing during the outbreak with your upper-level medical institutions and potential clients in your community?
PROBE: how were changes in the delivery of SRH services communicated to clients? How was important COVID-19 information communicated to clients?

Access to essential medicines

16. Was the access to essential medicines, including regular medicines such as birth control pills, contraceptive methods impacted because of the lockdown/shutdown of the city during the COVID-19?

COMMENT*: Shutdown can be replaced with lockdown/restrictions depending on what the COVID-19 response was in country using this guide*

PROBE: ask about medicines for all the study focus SRH services

17. How has the outbreak affected the access to essential medicines in SRH for the patients and the general population?

PROBE: for each- contraceptive methods, abortion, post abortion care, STI prevention and treatment and GBV care and support

COMMENT: Probe separately for routine medication (such as antibiotics, analgesics etc where applicable)

Health systems financing

18. How did the COVID-19 epidemic affect the availability of funds at your facility?
PROMPT: Inquire about effect on the financing and maintenance of essential SRH services and possible disruptions due to the pandemic, availability of additional government funding allocated to assuring essential health services.

> ***COMMENT:*** *This section should be informed by the existing health financing policy (i.e. National health insurance, existing SRH related exemptions and waiver systems) or COVID-19 specific directives on financing.*

Leadership and governance

19. What are your perceptions on the roles and responsibilities of providing SRH services during outbreaks like COVID-19?
    PROBE: where does the primary responsibility to ensure continuity of services lie? Who are the enablers, implementers?
20. How did your facility respond to the emergency and manage to ensure the SRH-related service delivery in your facility?
    PROMPT: Probe for country-specific plan, policies for maintaining SRH service provision during the pandemic.
    PROBE: separately for, family planning, STI and abortion services, services prioritized by the facility during the pandemic.

Health service provider perceptions on the delivery of specific SRH services in the context of COVID-19

A. Gender based Violence

   Perceptions of changes in the nature and magnitude of violence against women, and especially domestic violence during COVID-19

   - Have you noticed any change in the number of women coming into the health facility reporting gender-based violence since the COVID-19 crisis began?
     - Follow up: Increase or decrease?
     - Follow up: Have the women coming in who report experiencing violence mostly been returning patients, or mostly been new patients?
     - Follow up: Have you noticed an increase in the frequency of abuse that women are experiencing since movement restrictions were put in place?
     - If possible, specify types of violence (physical, sexual, psychological by a partner), non-partner sexual abuse, other

   - Do you think that the violence women have been experiencing has become more severe during the crisis, for example has there been an increase in injuries? Violence during pregnancy?
     - Follow up: Have the types of violence that women are reporting changed? How so?

   Nature of service provision during COVID-19, whether services for VAW are being changed or adapted and if safety considerations are adequately addressed?

   - Since the start of COVID-19, has your health facility changed or adapted services for survivors 'of violence?
     - Probe: (if not mentioned) In some health centers, providers have maintained 'case management' using video calls, or text messages. Did your health center make use of any new technologies to maintain services? If so, did you develop a protocol to ensure safety of survivors?

Access to /use of health services for VAW and related challenges

- Some health centers have had to deal with shortages of personal protective equipment and medications that have impacted their ability to offer services during the COVID-19 crisis. Have there been any new barriers to delivering services to survivors at your health facility?
    - What are these barriers and what approaches are being used to overcome these barriers?

- In a lot of places, external referral systems have also been impacted by the crisis, such as lower numbers of available spaces in shelters due to the need for physical distancing, or NGOs that have been temporarily shut down. To your knowledge, have any changes have been made to your external referral systems? What are these?
    - Follow up: Are any of the services to which you typically refer survivors available in a more limited capacity, or no longer available due to the crisis?

Attitudes towards provision of SRH services in the context of COVID-19

- Do you think it is essential to provide services for VAW during COVID-19? Why do you think so?

B. Induced abortion

Perceptions of changes in the demand for abortion during COVID-19.

- Have you noticed any change in the number of women seeking abortion services at your facility during COVID-19?
    - Follow up: Increase or decrease?
    - Follow-up: any changes in the abortion methods requested?
    - What do you think were the factors driving this change?

Delivery of safe abortion services during COVID-19?

- Where and how can people seek contraceptive services during COVID-19?
    - How many methods of abortion were available in your health facility during COVID-19? Which method of abortion did you suggest during COVID-19? Why?
    - What challenges did you meet in providing safe abortion services during COVID-19? What approaches did you use to overcome these challenges?

Attitudes towards provision of SRH services in the context of COVID-19

- Do you think it is essential to provide safe abortion services during COVID-19? Why do you think so?

C. Contraceptive services

Perceptions of changes in the demand for contraception services during COVID-19.

- Have you noticed any change in the number of women seeking contraception services at your facility during COVID-19?
    - Follow up: Increase or decrease?
    - Follow-up: any changes in the contraception methods requested?
    - What do you think were the factor driving this change?

Delivery of contraception services during COVID-19?

- Where and how can people seek contraceptive services during COVID-19?

- How many kinds of contraceptive methods were available in your health facility during COVID-19?
- What are the new/ altered ways by which contraceptive services are provided as a response to COVID-19?
- What challenges did you meet in providing contraceptive services during COVID-19? What approaches did you use to overcome these challenges?

Attitudes towards provision of SRH services in the context of COVID-19

- Do you think it is essential to provide contraception services during COVID-19? Why do you think so?

D. <u>Prevention and treatment for sexually transmitted infections</u>

Perceptions of changes in the demand for care and treatment for STI during COVID-19.

- Have you noticed any change in the number of women seeking care and treatment for STI at your facility during COVID-19?
  - Follow up: Increase or decrease?
  - What do you think were the factor driving this change?

Availability of STI care and treatment during COVID-19

- Where and how can people seek care and treatment for STI during COVID-19?
  - What are the new/ altered ways by which care and treatment for STI services are provided as a response to COVID-19?
  - Were all the medicines and supplies for providing the care and treatment available during COVID-19?
  - What challenges did you meet in providing care and treatment for STI services during COVID-19? What approaches did you use to overcome these challenges?

Attitudes towards provision of SRH services in the context of COVID-19

- Do you think it is essential to provide prevention and treatment for STIs during COVID-19? Why do you think so?

3. Perceived psychosocial effects of COVID-19
   - Did you experience stress/anxiety/depression during COVID-19?
   - How would rate your psychosocial health?
   - What role did COVID-19 play in influencing your psychosocial health?
   - In what way?
   - What do you think is the psychosocial impact of COVID-19 on people?
   - Do you think the impact was the same for men and women? If yes, why?
   - If no, in what ways is the psychosocial impact different?
   - What do you think is the psychosocial impact of COVID-19 on families?
   - What do you think is the psychosocial impact of COVID-19 on communities?

4. Final questions
   - On-going concerns about COVID-19 and its possible impact
     - Are there any other concerns about COVID-19 and its impact on you, your family or in general that you wish to express?
   - Needs related to accurate information (probe further information needs – NB consider how to provide information if necessary)

  - Was accurate information readily available and accessible during COVID-19?
  - Was it easy to understand?
  - Was the information that was available adequate?
  - Was there additional information you would have liked to obtain but was missing?
  - Are there better ways of making accurate information accessible to you and others?
- Needs related to sexual reproductive health services (probe further SRH needs – NB consider how to support/provide if necessary)
- Do you think SRH services were responsive to the needs of patients during COVID-19?
- If no, which of needs were not met?
- Which SRH services are most important, that should be available even in health emergencies like COVID-19?
- How do you think provision of SRH services can be supported during other health emergencies like COVID-19? E.g. barriers in seeking SRH services during COVID

Additional themes will be added as appropriate for each target group and questions revised accordingly (with attention to first person / third person views), and across the three phases of the study.
