# Supplementary material for: Health systems analysis and evaluation of the barriers to availability, utilisation and readiness of sexual and reproductive health services in COVID-19-affected areas: a WHO mixed-methods study protocol
Source: BMJ Open. 2022 Jun 1;12(6):e057810. doi: 10.1136/bmjopen-2021-057810 (PMC9160592; doi:10.1136/bmjopen-2021-057810)
Supplement: Author's manuscript [file bmjopen-2021-057810.draft_revisions.pdf]

BMJ Open is committed to open peer review. As part of this commitment we make the peer review history of every article we publish publicly available.

When an article is published we post the peer reviewers' comments and the authors' responses online. We also post the versions of the paper that were used during peer review. These are the versions that the peer review comments apply to.

The versions of the paper that follow are the versions that were submitted during the peer review process. They are not the versions of record or the final published versions. They should not be cited or distributed as the published version of this manuscript.

BMJ Open is an open access journal and the full, final, typeset and author-corrected version of record of the manuscript is available on our site with no access controls, subscription charges or pay-per-view fees (http://bmjopen.bmj.com).

If you have any questions on BMJ Open's open peer review process please email info.bmjopen@bmj.com

BMJ Open

Health systems analysis and evaluation of the barriers to availability, utilization and readiness of sexual and reproductive health services in COVID-19 affected areas: A WHO mixed methods study protocol

Journal:	*BMJ Open*
Manuscript ID	bmjopen-2021-057810
Article Type:	Protocol
Date Submitted by the Author:	29-Sep-2021

Complete List of Authors:	Nahyuha Chomi, Eunice; Research Institute of Health Sciences<br>Kouanda, Seni; Institut de Recherche en Sciences de la Sante<br>Kim, Caron; World Health Organization, Department of Sexual and Reproductive Health and Research. Preventing Unsafe Abortion Team<br>Jen, Sothornwit; Khon Kaen University, Department of Obstetrics and Gynaecology<br>Bahamondes, Luis; State University of Campinas – UNICAMP, Obst & Gynecol<br>Cecatti, Jose; University of Campinas, Obstetrics and Gynecology<br>Lumbiganon, Pisake; Khon Kaen University, Faculty of Medicine<br>Emefa, Modey; University of Ghana<br>Brizuela, Vanessa; WHO, Department of Reproductive Health and Research<br>Kuganantham, Hamsadvani; World Health Organization, Department of Reproductive Health and Research<br>Ali, Moazzam; Organisation mondiale de la Sante,

Keywords:	COVID-19, PUBLIC HEALTH, QUALITATIVE RESEARCH

SCHOLARONE™
Manuscripts

Manuscript Protocol

Title page:

Health systems analysis and evaluation of the barriers to availability, utilization and readiness of sexual and reproductive health services in COVID-19 affected areas: A WHO mixed methods study protocol.

1. Nahyuha Chomi, Eunice - Reproductive Health Unit, Institute for Research in Health Sciences, Ouagadougou, Burkina Faso. E. Mail: e_chomi@yahoo.com
2. Kouanda Seni-Reproductive Health Unit, Institute for Research in Health Sciences, Ouagadougou, Burkina Faso and African Institute of Public health. E. Mail: senikouanda@gmail.com
3. Kim Caron- Department of Reproductive Health and Research, World Health Organization, Avenue Appia 20, 1211 Genève 27, Switzerland. E. Mail: kimca@who.int
4. Sothornwit Jen- Department of Obstetrics and Gynaecology, Faculty of Medicine, Khon Kaen University, Khon Kaen, Thailand. E. Mail: jeneration_x021@hotmail.com
5. Bahamondes Luis- School of Medical Sciences, University of Campinas (UNICAMP), Campinas, Brazil. E. Mail: bahamond@caism.unicamp.br
6. Cecatti Jose Guilherme- School of Medical Sciences, University of Campinas (UNICAMP), Campinas, Brazil. E. Mail: cecatti@unicamp.br
7. Lumbiganin Pisake- Faculty of Medicine, Khon Kaen University, Khon Kaen, Thailand. E. Mail: lumbiganon.pisake@gmail.com
8. Modey Emefa-Department of population, family and Reproductive Health, University of Ghana, Legon-Accra, Ghana. E. Mail: ejmodey@ug.edu.gh
9. Brizuela Vanessa- Department of Reproductive Health and Research, World Health Organization, Avenue Appia 20, 1211 Genève 27, Switzerland. E. Mail: brizuelav@who.int
10. Kugananthan Hamsa- Department of Reproductive Health and Research, World Health Organization, Avenue Appia 20, 1211 Genève 27, Switzerland. E. Mail: kugananthamh@who.int
11. Ali Moazzam* on behalf of WHO HRP social Science Research Team**- Department of Reproductive Health and Research, World Health Organization, Avenue Appia 20, 1211 Genève 27, Switzerland. E. Mail: alimoa@who.int

***Corresponding author:** Moazzam Ali (alimoa@who.int)
Department of Reproductive Health and Research, World Health Organization, Avenue Appia 20, 1211 Genève 27, Switzerland

KEYWORDS
Contraception, sexual and reproductive health, comprehensive abortion services, COVID-19, facility assessment, gender-based violence, sexually transmitted infections

For peer review only

Abstract

Introduction The novel COVID-19 disease has led to an unprecedented increase in demand on health systems to care for people infected, necessitating the allocation of significant resources, especially medical resources, towards the response. This, compounded by the restrictions on movement instituted may have led to disruptions in the provision of essential services, including sexual and reproductive health (SRH) services. This study aims to assess the availability of contraception, comprehensive abortion care, sexually transmitted infection (STI) prevention and treatment and gender-based violence (GBV) care and support services in local health facilities during COVID-19 pandemic. This is a standardized generic protocol designed for use across different global settings.

Methods and Analysis This study adopts mix methods to assess health facilities' SRH service availability and readiness, and clients' and providers' perceptions of the availability and readiness of these services in COVID-19-affected areas. The study assesses: (1) Perceptions of clients (and their partners) and healthcare providers perspectives, using qualitative methods, and (2) assessment of infrastructure availability, readiness to provide SRH services through reviews and facility service statistics for clients. The health system assessment will use a cross-sectional panel survey design with two data collection points to capture changes in SRH services availability as a result of the COVID-19 epidemic. Data will be collected using focus group discussions, in-depth interviews and a health facility assessment survey.

Ethics and Dissemination Ethical approval for this study was obtained from the WHO Scientific and Ethics Review Committee (protocol ID CERC.0103) and each study site also obtained ethical and regulatory approvals. The study will be conducted according to the principles outlined in the Declaration of Helsinki and its amendments. Results of this study will be disseminated in peer-reviewed scientific journals.

Article summary

Strengths and limitation of this study

- The study approach and design enable a comprehensive analysis of the barriers to availability, utilization and readiness of sexual and reproductive health services during COVID-19, as well as the post-pandemic recovery as transmission is contained.
- The use of a mainly qualitative approach places women's rights and needs during health emergencies at the center of the debate, underscoring the need for more responsive policies.
- The inclusion of partners will enhance understanding of gender dynamics and support efforts towards identifying strategies to enhance positive male involvement and engagement in women's sexual reproductive health and rights.
- A possible limitation is that the study will be conducted during and after one of the waves of the pandemic, at which stage there may have been changes in the health system as lessons are learnt.
- Another limitation is that the estimation of the recovery period may be difficult in settings where the pandemic is yet to be brought under control. This part of the analysis may be subject to delays until an appropriate period is determined.

For peer review only - http://bmjopen.bmj.com/site/about/guidelines.xhtml

Word count: 4172

INTRODUCTION

The novel COVID-19 disease, which was first reported in December 2019 and rapidly spread globally, has led to an unprecedented increase in demand on health systems to care for people infected, necessitating the allocation of significant resources, especially medical resources, towards the response. The increased demand, compounded by the restrictions on movement instituted as part of containment measures may have led to disruptions in the provision of essential services, including sexual and reproductive health (SRH) services [1,2].

The novel coronavirus is new to humans and only limited scientific evidence is available on the impact of COVID-19 on SRH service delivery. However, lessons from the Ebola and Zika virus outbreaks have highlighted the severe disruptions in SRH services that expose women and girls in particular to preventable health risks [2,3]. Some services may be unavailable due to either facilities and health workers being repurposed to care for COVID-19 patients, patient safety concerns, movement restrictions disrupting travel to hospitals, supply chain disruptions or a reduction in health workers because of increasing numbers being themselves infected by COVID-19 [4,5]. In addition, overwhelmed with COVID-19 cases, clinical staff may not have the time or personal protective equipment (PPE) needed to provide family planning counselling and commodities [5]. Recent evidence suggests disruptions lasting 3-6 months in 2020 left between 4-23 million women in low- and middle-income countries unable to access modern contraceptives, a projected 1.4 million (500,000-2.7 million) unintended pregnancies and an additional 31 million cases of gender-based violence (GBV) [1, 6-9]. Furthermore, studies have modelled the potential impact, showing that even a 10% reduction in essential SRH services could lead to an estimated 15 million unintended pregnancies, 3.3 million unsafe abortions and 29, 000 additional maternal deaths during the next 12 months [7-10]. Continuity of essential health services while keeping people safe during the response to disease outbreaks such as the COVID-19 pandemic is therefore essential for the prevention of both direct and indirect mortality [4,5].

This is a generic standardized protocol designed to maximize the likelihood that data are systematically collected and shared rapidly in a format that can be easily reproducible, aggregated, tabulated and analyzed across many different settings globally and be useful as templates for use in health emergencies in the future. This will facilitate the comparison of results across regions and countries and will potentially improve the quality of observational studies by identifying and minimizing biases. Given that use in different settings will require some adaptation, these possibilities have been highlighted throughout the protocol.

The introduction should be updated with country specific data on COVID-19 epidemiology and current research findings prior to submission to local/country national institutional review boards

For peer review only - http://bmjopen.bmj.com/site/about/guidelines.xhtml

The main aim of this study is to assess the availability of contraception, comprehensive abortion care, STI prevention and treatment and GBV care and support services in local health facilities during COVID-19 pandemic. The four specific objectives of the study are:

1. To explore the availability of, and health facility readiness to provide sexual and reproductive health services in areas most affected by COVID-19.
2. To qualitatively assess the availability and quality of services and barriers to the utilization of these services from clients' and providers' perspectives in the selected COVID-19 affected areas.
3. To assess the post-pandemic recovery* of the facilities in the provision of these services in comparison to the pandemic period.
4. To enhance the SRH service capacity in COVID-19 through advocacy, policy briefs, media dissemination and academic papers towards national and regional stakeholders including policymakers, academia, healthcare providers and the community.

**Post-pandemic recovery refers to the period when ideally health facilities have been able to recover from the disruption in service provision, following reduced transmission levels. Caution should be applied to the term "post-pandemic" as services may be still impacted even with lower levels of transmission. Therefore, the research team should be cognizant of the distinct time periods in their facility assessments.*

METHODS AND ANALYSIS

Study design

This is a repeated cross-sectional study, using both quantitative and qualitative methods. The two data collection points, baseline and end-line, will be 9 to 12-months apart. The aim of having two data collection points is to document and share the local evidence with authorities on the SRH services at baseline, and then at end line to track and demonstrate the changes and improvement in services over time. The WHO situational level assessment of COVID-19 transmission will be used as the basis for determining when to conduct baseline and endpoint data collection [11]:

- Level 0: no known transmission in the preceding 28 days, no restriction on daily activities.
- Level 1: basic measures in place, clusters of cases reported controlled with basic measures, limited and transient disease.
- Level 2: local transmission from imported cases to close contacts. Contact tracing, physical distancing and quarantine measures can contain the spread.
- Level 3: community transmission, where source of infection is untraceable, outbreak rapidly spreads in clusters.
- Level 4: disease outbreak has become an epidemic, where there are major clusters of infection all over the country, high number of deaths and it is very difficult to control transmission without strict containment measures.

Countries should time the baseline and end-line data collection as follows: Baseline data collection is proposed when countries are experiencing level 3 or 4 transmission (referred to here as peak transmission/pandemic period), as disruptions in service provision are likely to occur during these periods. End-line data collection is proposed when countries have tentatively

For peer review only - http://bmjopen.bmj.com/site/about/guidelines.xhtml

managed to contain the spread and are at levels 0,1 or 2 (referred to here as post-pandemic period/after the pandemic) after experiencing levels 3 or 4.
Given the dynamic nature of the epidemic, where each country is experiencing varying levels of COVID-19 transmission (and the challenges from the resurgence of variants and sudden increase in the number of cases), it is difficult to assume standardized pandemic conditions at country level. Therefore, the time interval between data collection points is just an estimation. For countries that have contained the spread, it may be decided to collect baseline data retrospectively, selecting the peak period. However, strategies to minimize or avoid recall bias should be used.

Study Setting and inclusion criteria

The study will be conducted in geographical areas selected based on the epidemic status. To get variation in responses, health facilities will be selected as focal points considering their qualification to provide SRH services and other criteria for the researchers to access community members for participation in the study. It is expected that variation in health facilities distributed within different geographical areas will provide access to communities of all socioeconomic backgrounds.

Due to various local realities and political factors, countries should select the research study sites based on geographical location, organization of SRH service delivery and epidemic status where COVID-19 is likely to have significantly affected service delivery (given that even within countries the transmission status differs, hence the differential impact on facilities in different geographical areas). Consultation with the Ministry of Health will help identify the areas most affected by COVID-19.

Study Population

The study population will be women seeking SRH services from the selected health facilities and their partners who accompany them, as applicable, and healthcare providers working there.

In contexts where it is not customary for men to accompany their partners when they seek SRH services, other options can be used to access them. Healthcare providers can be medical doctors, nurses, midwives, nurse assistants, allied health professionals and other cadres depending on the norms and standards for the provision of SRH services in different country contexts.

Sample selection and sampling

Each sample will be selected based on specific criteria, as well as specific country contextual factors.

For peer review only - http://bmjopen.bmj.com/site/about/guidelines.xhtml

Health facility selection

The minimum criteria for selection should include availability of human resources, primarily the qualification to provide contraception, safe abortion, including the treatment of complications and the provision of post-abortion care, STI care and treatment and support for women experiencing GBV. In addition, the diversity of hospital capacity, administrative rank, urban/rural setting and the willingness of the providers in charge to participate in the study, as well as how the COVID-19 response has been organized in terms of treatment centers. These criteria for selection of health facilities within the geographical sites were made to encourage a representative mix of facilities. The same health facilities will be used for both data collection points to highlight the changes in SRH delivery due to COVID-19 and post-pandemic recovery.

Given the dynamic nature of the pandemic *some flexibility should be given to all study sites in the selection of facilities, maintaining the minimum criteria specified above*

Qualitative sample

Selection of women

Women will be recruited for in-depth interviews (IDIs) and focus group discussions (FGDs). The women will be purposively selected to obtain a sample of women and their partners. Selection will be based on 1) being of reproductive age (18-49) and 2) having sought or tried to receive SRH services from local health facilities. The study will use gatekeepers, who will be healthcare provides not involved in the study to approach the women as they leave the hospital. Their role will be limited to the identification and introduction of potential participants to the study team.

For the IDIs, a sample of 10~15 women (or until saturation) and 6~12 partners (or until saturation) will be recruited. For countries with more enrolled hospitals in the study, the maximum number of the IDIs will be with 10 women (or until saturation) at reproductive age seeking for reproductive healthcare services, and with 5 partners (or until saturation) per hospital. This will be done as an exit interview and the women will be consecutively selected until the desired sample (or saturation) has been achieved.

For FGDs, gatekeepers will also ask participants about their level of comfort discussing experience seeking SRH issues in a group before being recruited. About 6-8 participants per FGD will be recruited, with the expectation of at least two focus groups per facility. Some female and male participants will also be invited for individual interviews after the FGDs.

The ages can be adjusted to account for differences in the nationally recognised reproductive age group in different contexts. Inclusion of girls younger than 18 years should take into consideration ethical issues of assent and consent.

To adjust to challenges posed by COVID-19 transmission, additional participants can be recruited through online recruitment from chat groups such as Facebook, Twitter or WhatsApp. Careful attention must be paid to the ethical issues of privacy and confidentiality of participants and

For peer review only - http://bmjopen.bmj.com/site/about/guidelines.xhtml

integrity of the researchers (transparency of aims, details, risks and benefits of the study, obtaining the necessary permission to join restricted groups and the presence of the researcher in the group). Other challenges of online recruitment that need attention include retention of participants, potential selection bias, verification of participant identity and comprehension of informed consent [12]. Care needs to be taken by hiding the names and faces (e.g., via video conference calls) of the online participants, responsible handling of participants' personal information to minimize the likelihood of embarrassment, loss of dignity or harm because of the online recruitment process to address the online privacy and confidentiality concerns. The moderators will ask the participants to give a short self-introduction, using pseudonyms/numbers, instead of their actual names, and not using video options. This includes not disclosing any of this information without the participants consent.

Selection of healthcare providers

Approximately 1 to 2 healthcare providers per hospital will be purposively selected and only those who 1) deliver SRH services, 2) are most knowledgeable about readiness and availability of SRH services 3) are stationed in the SRH clinic and have been working at the clinic for at least 6 months before the pandemic started will be selected for inclusion in the study. This information will be obtained from the facility in-charges.

Quantitative sampling

Selection of Women

In each hospital, a maximum of 5 -6 clients from each section (post-abortion care, antenatal care, delivery care, post natal care, family planning, GBV, STI, abortion) will be consecutively selected to achieve up to 40 -60 clients per hospital.

Selection of healthcare providers

One healthcare provider, preferably most knowledgeable in the health facility about SRH services provided, as determined by seniority, position or function, for example, will be selected to assist in the health facility assessment.

Data Collection

FGDs and IDIs will be used to understand client's perspectives on their experiences in accessing SRH services during and after peak transmission and healthcare providers' perspectives of SRH service availability and readiness in COVID-19-affected areas. The health system assessment will be used to assess the health system response to COVID-19. Study teams should be aware of the possibility that healthcare provider burnout or heavy workload may affect willingness or ability to participate in the study. Adaptations to data collection should be made to avoid overburdening the healthcare providers and clearly explained during recruitment.

Data Collection tools

There will be five sets of tools for clients- 1 FGD guide for women (published as **supplementary file S1**), 1 FGD guide for men/partners (published as **supplementary file S2)**, 1 interview guide

For peer review only - http://bmjopen.bmj.com/site/about/guidelines.xhtml

for women, 1 interview guide for women (published as **supplementary file S3**) and a interview guide for men (published as part of **supplementary file S4**); and 1 interview guide for healthcare providers (published as **supplementary file S5**).

For the facility assessment, a facility and readiness assessment questionnaire has been developed based on the following validated tools [13-16]:

(1) WHO Service Availability and Readiness Assessment (SARA) guide
(2) WHO Hospital Readiness Checklist
(3) WHO safe abortion assessment tool
(4) GBV Gender-Based Violence Quality Assurance Tool

The facility and readiness assessment tool comprises five modules: (1) health services continuation (; (2) family planning services; (3) abortion services; (4) STI and (5) GBV; in line with the study SRH focus areas. The following indicators will be used the assess the availability and readiness:

(1) Policies and plans
(2) Service maintenance and referrals
(3) Infrastructure
(4) Commodities
(5) Human resources

Each study site will adapt the tool to its specific context, taking into consideration the existing policies, SRH treatment guidelines, staffing norms and standards, types of facilities, national essential medicines lists and national health information systems among others. After adaptation to specific country context all tools should be piloted before being used.

Data Collection Methods

Participant Informed Consent Process

All study participants will be taken through a detailed informed consent process (for participation and audio-recording), which will be documented and those who agree to participate in the study will be asked for signed written (oral consent for those who cannot write, or for situations that necessitate online data collection) consent.

Given that data collection will be conducted during COVID-19, an assessment of the risks to the study team and participants should be made. The decision of whether to conduct face-to-face or remote data collection should be based on the risk assessment, followed by context-specific recommendations on adaptations to the original data collection process. Remote data collection will necessitate adaptation to the length of interviews and discussions, rapport building, oral informed consent, privacy and confidentiality measures as well as making special arrangements where access to internet and mobile connectivity is limited [17]. Face-to-face data collection will require special arrangements to ensure the safety of the study team and participants, following WHO as well as country-specific safety protocols, including educating and training the research team and participants about COVID-19, provision of PPE and hygiene supplies, mandatory hygiene practices and sanitization of venues and equipment and physical distancing [18].

For peer review only - http://bmjopen.bmj.com/site/about/guidelines.xhtml

The FGDs and IDIs will be conducted at times and in venues that are considered both convenient and safe for the participants to freely discuss the subject matters. In addition, qualified researchers with experience in conducting IDIs and facilitating FGDs will be trained to ensure the validity of the data collection and will be selected based on the gender of the participants. The FGDs and IDIs will be semi-structured and follow a topic guide specific to each group of participants but will take place as a conversation in which the researchers promote a safe, comfortable environment to enable a comprehensive record. In addition to the semi-structured interview, narrative interview techniques may be used depending on the participants' narratives about life events and reproductive healthcare needs.

The FGDs and IDIs will be audio-recorded. Prior consent for recording will be sought again from each participant. Anonymity of the participants will be ensured by removing any personal or family identifiers and all recorded sessions will be coded for purposes of identification with a date, geographical site and session number. After each session, the recording will be sent to the team supervisor for secure storage for transcription. The audio files will be encrypted and sent to an electronic database which will be shared with WHO. Therefore, only the research team will be authorized to listen to the recordings. These audio recordings will be retained until they have been transcribed and checked for accuracy, after which they will be destroyed.

The FGDs will explore knowledge about COVID-19, care-seeking during COVID-19 and risk perceptions and availability of SRH services (end-line FGD will also explore post-pandemic services in comparison to the pandemic period). Before starting the FGD, the facilitators will collect socio-demographic data for each participant, build rapport with and among participants and set ground rules to ensure positive group dynamics that foster an effective and rich discussion. The facilitators will also use this time to know and understand the emic categories (field research and viewpoints obtained from within the social group, from the perspective of the subject) used by locals to describe their perceptions and practices related to the study topics. Each FGD is expected to last approximately 60 – 90 minutes.

IDIs with women will explore the psychosocial effects of COVID-19 on fertility desires, knowledge of COVID-19, risk perceptions and concerns about effects on SRH, care seeking behaviors, experience and barriers in accessing SRH services reproductive health, particularly comprehensive abortion, STI prevention and treatment and GBV care and support, and their related needs for accurate information and reproductive health services. IDIs with partners will explore their knowledge of COVID-19, risk perceptions and concerns about effects on SRH, their influence on the access to SRH services of their partners and find out what role they play when their partners require SRH services like contraception, comprehensive abortion care, STI prevention and treatment and GBV care and support. Each interview is expected to last approximately 40 – 60 minutes.

In some countries, abortion is illegal, posing challenges in data quality since participants, fearing reprisals, may not provide accurate responses to abortion-related questions. Necessary precautions should be taken to ensure the interviews are conducted in a supportive and non-

For peer review only - http://bmjopen.bmj.com/site/about/guidelines.xhtml

judgmental manner, encouraging the participants to respond freely. These include selection and training of interviewers to enable them to overcome their biases and stereotypes about abortion, building trust and rapport with participants and selection of interview venue to guarantee privacy [19].

Interviews with healthcare providers will be based on WHO's six building blocks framework [20] with a focus on the delivery of the focus SRH services during COVID-19; their perceptions on the roles and responsibilities of different parties to provide these services in the COVID-19 context; health system capacity to provide good quality of care for people during COVID-19; training needs, attitudes, biases about contraception, abortion, STI and GBV in the context of COVID-19; and perceived psychosocial effects on men and women, their families and local communities.

The health availability and readiness assessment will be implemented in all the selected facilities using a cross-sectional survey design to highlight gaps or service delivery issues during COVID-19 and recovery in service availability and readiness in the same health facilities. The assessment will also include a review of health facility records in family planning and contraception, STI care and treatment and care and support for women who have experienced GBV to assess the availability, type and range of commodities offered. A follow-up assessment will be done at end-line. The follow-up survey plans to assess the recovery in service availability and readiness in the same health facilities. The research team will fill out the data extraction tool and the questionnaire with the assistance of the most knowledgeable person in the health facility about SRH services provided (senior health care provider and/or administrator) for each section of the questionnaire. The assessment will also include a questionnaire to the clients of the health facility.

The client questionnaire contains questions related to general characteristics of clients and their experience in seeking SRH services. All the selected hospitals will be required to collect this data from all clients seeking SRH services once every month throughout the study period, to capture trends in service availability and utilization.

Data Analysis

Health facility assessment data will use the presence of the tracer items for the provision of these services, such as availability of guidelines, staff and essential commodities. Data will be entered into an electronic database using data entry programmes such as Epi data and CSPro. Study sites using electronic data collection may skip this step and for WHO supported centres implementing the study an online platform (OpenClinica) will be used. Descriptive analysis will be used to illustrate the basic characteristics of the different facilities, including the monthly number of clients, types of procedures provided, number of medical staff, stocks of drugs, etc.

Qualitative analysis will form the main analysis for this study, beginning while data collection is ongoing to assess progress and determine any problems. The audio-recorded data will be transcribed verbatim and de-identified by using ID numbers in place of names. Where required, transcriptions will be translated to English and back-translated and analyzed using content

For peer review only - http://bmjopen.bmj.com/site/about/guidelines.xhtml

analysis, according to the suggested steps by Elo & Kyngäs (2008) [21], as illustrated in the Table (see Table 1). The WHO team based in Geneva, in conjunction with the study principal investigator (PI) and project team, will support and monitor the data analysis.

Table 1. Planned analytical procedures for content analysis

Phase	Procedures
Preparation	Reading through verbatim transcriptions of the interviews several times to familiarize with the data, gain an understanding of what has been expressed, selecting the unit of analysis, deciding on the analysis of manifest content.
Organizing	Open coding and creating categories, grouping codes under higher order headings, formulating a general description of the research topic through generating categories and subcategories as abstracting.
Reporting	Reporting the analysis process and the results through models, conceptual systems, conceptual map or categories, and a story line.

Data management and access

Data management plans should include information about how data will be stored, including levels of protection, who will have access to the data and when it would be destroyed, how data will be transferred (if needed) securely. In countries where data protection legislation exists, protocols should specify that data would be handled in accordance with those policies.

Quantitative data

Data collectors will be trained on data collection, transmission, verification, storage and primary analysis to assess errors. Data can be collected electronically, or on paper.

Qualitative data

The quality and trustworthiness of the qualitative data collected will be assured through triangulation, as we will conduct interviews with different parties, including health professionals, health workers, and clients. The process of the study will be clearly documented to ensure the transparency and the rigor of the study.

Ethics and dissemination

Scientific approval has been obtained from WHO research project review panel (RP2). Ethical approval for this study was also obtained from the WHO Ethics Review Committee (protocol ID CERC.0103). Each study site is required to obtain the necessary ethical and regulatory approvals that are required in the corresponding country. Ethical considerations of informed consent, voluntary participation, privacy and confidentiality, anonymity and compensation for incidental costs (the decision to offer compensation and its value will depend on the specific country context and respective local policies) will be respected and detailed in the informed consent process.

For peer review only

Given the sensitive nature of the data being collected, special care will be taken to ensure the "do no harm" principle is respected. This will include making arrangements with locally available support services (medical, psychosocial, legal) where participants can be referred, training the research team to respond to and provide immediate emotional support to study participants or for situations where support services are not locally available or are inadequate [22, 23].

At country level the results will be presented to policy makers, researchers, managers through policy briefs and workshops in collaboration with the WHO office. The results will also be disseminated among the communities/participants through online platforms/text messages, which will be provided to the participants by the data collectors/health workers. In addition, the results will also be presented to the scientific and funding community in collaboration with WHO country and regional offices, by communication and manuscript publications in peer-reviewed international journals

Discussion

This is a generic standardized protocol designed to be used across many different settings globally and to be useful as templates for use in health emergencies in the future. This will facilitate the comparison of results across regions and countries.

This study provides a unique opportunity to assess the availability, utilization and readiness of the SRH services during the COVID-19 pandemic. The main lesson learned will be the adaptations of the health system in pandemic situations and what can be done to ensure continuity of essential SRH services.

The mixed methods approach and panel design with two data collection points enable a comprehensive analysis of the barriers to availability, utilization and readiness of sexual and reproductive health services during COVID-19, as well as the post-pandemic recovery as transmission is contained.

This study is well placed to advocate for the development and strengthening of policies and services that are responsive to the needs of women and girls during health emergencies, given the potential to exacerbate existing gender and social inequalities and increase the vulnerability of women and girls to preventable health risks. The use of a mainly qualitative approach places women's rights and needs at the center of the debate. The inclusion of partners will enhance understanding of gender dynamics and support efforts towards identifying strategies to enhance positive male involvement and engagement in women's sexual reproductive health and rights.

Authors' contributions

MA and CRK conceptualized and designed the protocol. EC and SK performed the first round of adaptation of the protocol to this manuscript under the supervision of MA and CRK. All authors contributed in the to improve the manuscript, read, and approved the final written manuscript.

For peer review only - http://bmjopen.bmj.com/site/about/guidelines.xhtml

Funding Statement

This research has been supported by German Federal Ministry of Health (BMG) COVID-19 Research and development funding to WHO. It has also been supported by the UNDP-UNICEF-WHO-World Bank Special Programme of Research, Development and Research Training in Human Reproduction (HRP); a co-sponsored programme executed by the World Health Organisation (WHO). The adaptation and implementation of this protocol in the HRP collaborating centres in Brazil, Burkina Faso, China, Ghana, Italy, Kenya, Pakistan, Thailand and the United Kingdom was also funded by the same. The views of the funding body have not influenced the content of this manuscript. This article represents the views of the named authors only and does not represent the view of their affiliated institutions.

Award Number: Not applicable.

Disclaimer

This report contains the collective views of an international group of experts and does not necessarily represent the decisions or the stated policy of the World Health Organization.

Competing interests

The authors declare that they have no competing interests.

Conflicts of interest

None declared

Patient and public involvement

There was no patient or public involvement in the design of this protocol.

Data availability statement

The data base will be made available upon request. Contact: alimoa@who.int

Acknowledgement

We thank all country research team members for their contributions to the wider discussions around this research.

** ***WHO HRP Social Science Research Team***: **Brazil** (Luis Bahamondes, Jose Guilherme Cecatti); **Burkina Faso** (Seni Kouanda, Eunice Chomi, Kadi Kadio); **China** (Kun Tang, Hanxiyue Zhang, Yifan Zhu, Yueping Guo, Ge Yang and Chunxiao Peng); **Ghana** (Deda Ogum Alangea, Kwasi Tropsey, Emefa Judith Modey); **Pakistan** (Rozina Karmaliani, Laila Ladak); **Thailand** (Pisake Lumbiganon, Jen Sothornwit); **Kenya** (Marleen Temmerman, Abdu Mohiddin, Ferdinand Okwaro); **Italy** (Massimo Mirandola, Maddalena Cordioli, Alessia Savoldi, Simone Garzon, Stefano Uccella, Ranieri Poli.) **UK** (Nigel Sherriff, Alexandra Sawyer, Jorg Huber, Jaime Vera, Debbie Williams).

For peer review only - http://bmjopen.bmj.com/site/about/guidelines.xhtml

WHO Secretariat (Moazzam Ali, Caron Kim, Hamsadvani Kuganantham, Igor Toskin, Vanessa Brizuela, Anna Thorson, Joy Jerop Chebet, Hugo Gamerro Abrego , Soe Soe Thwin, Armando Seuc)

Acronyms

COVID-19	Corona Virus Disease 2019
CSPro	Census and Survey Processing System
FGD	Focus Group discussion
GBV	Gender Based Violence
IDI	In-depth Interview
RP2	Research Project Review Panel
SRH	Sexual and Reproductive Health
STI	Sexually Transmitted Infections
WHO	World Health Organisation

References

1. Riley T, Sully E, Ahmed Z, Biddlecom A. Estimates of the potential impact of the COVID-19 pandemic on sexual and reproductive health in low- and middle-income countries. *Int Perspect Sex Reprod Health* 2020; 46:73–76. doi: 10.1363/46e9020.
2. Addressing human rights as key to the COVID-19 response. Geneva: World Health Organization; April 2020 (WHO/2019-nCoV/SRH/Rights/2020.1; https://www.who.int/publications/i/item/addressing-human-rights-as-key-to-the-covid-19-response (accessed August 15, 2020)
3. Sexual and Reproductive Health and Rights during the COVID-19 pandemic: A joint report by EPF & IPPF EN. April 2020. https://www.ippfen.org/sites/ippfen/files/2020-04/Sexual and Reproductive Health during the COVID-19 pandemic.pdf (accessed August 16, 2020)
4. COVID-19: operational guidance for maintaining essential health services during an outbreak: interim guidance. 25 March 2020. Geneva: World Health Organization. 2020 WHO/2019-nCoV/essential_health_services/2020.1; https://apps.who.int/iris/handle/10665/331561 (accessed on August 15, 2020).
5. Maintaining essential health services: operational guidance for the COVID-19 context. World Health Organisation; 2020. https://apps.who.int/iris/handle/10665/332240 (Accessed August 15, 2020)
6. Impact of the COVID-19 pandemic on family planning and ending gender-based violence, female genital mutilation and child marriage: pandemic threatens achievement of the transformative results committed to by UNFPA. New York, United Nations Population Fund; 2020 https://www.unfpa.org/resources/impact-covid-19-pandemic-family-planning-and-endinggender-based-violence-female-genital-mutilation (accessed on August 15, 2020).
7. Millions more cases of violence, child marriage, female genital mutilation, unintended pregnancy expected due to the COVID-19 pandemic [Internet]. Unfpa.org. 2020 [cited Sept 24 2020]. Available from: https://www.unfpa.org/news/millions-more-cases-violence-child-marriage-female-genital-mutilation-unintended-pregnancies#

For peer review only - http://bmjopen.bmj.com/site/about/guidelines.xhtml

8. Coronavirus Disease (COVID-19) Pandemic UNFPA Response Plan. June 2020 https://www.unfpa.org/sites/default/files/resource-pdf/UNFPA_Global_Response_Plan_Revised_June_2020_.pdf (accessed August 16, 2020)
9. United Nations Population Fund. Impact of COVID-19 on family planning: What we know one year into the pandemic. Technical note. New York, UNFPA;2021
10. Julia Hussein. COVID-19: What implications for sexual and reproductive health and right globally? *Sex Reprod Health Matters* 2020;28(1):1746065 DOI:10.1080/26410397.20201756065.
11. World Health Organization. Considerations for implementing and adjusting public health and social measures in the context of COVID-19. Interim guidance. Geneva, World Health Organization;2020 https://www.who.int/publications/i/item/considerations-in-adjusting-public-health-and-social-measures-in-the-context-of-covid-19-interim-guidance (Accessed Sept 24, 2020)
12. Gelinas L, Pierce R, Winkler S, et.al. Using social media as a research recruitment tool: ethical issues and recommendations. *Am J Bioeth 2017*;17(3):3-14
13. World Health Organization. Service availability and readiness assessment (SARA): implementation guide. Geneva: World Health Organization; 2015 http://www.who.int/healthinfo/systems/sara_implementation_guide/en/. (Accessed Sept 24, 2020)
14. World Health Organization. Safe abortion: technical and policy guidance for health systems. 2nd ed. Geneva: World Health Organization; 2012 http://www.who.int/reproductivehealth/publications/unsafe_abortion/9789241548434/en/. (Accessed Sept 24, 2021)
15. JHPIEGO. 2018. Gender-Based Violence Quality Assurance Tool: Standards for the Provision of High-Quality Post-Violence Care in Health Facilities. https://www.who.int/reproductivehealth/publications/post-violence-care-in-health-facilities/en/ (Accessed Sept 24, 2021)
16. World Health Organisation. Rapid Hospital Readiness Checklist: Harmonized Health Service Capacity Assessment in the Context of the COVID-19 Pandemic. Interim Guidance. Geneva: World Health Organization; 2020 https://apps.who.int/iris/handle/10665/332779
17. Hensen B, Mackworth-Young CRS, Simwinga M, et al. Remote data collection for public health research in the covid-19 era: ethical implications, challenges and opportunities. *Health Policy Plan* 2021;czaa158 https://doi.org/10.1093/heapol/czaa158
18. IMPACT. 2020. SOPs for Data Collection during COVID-19. https://www.reachresourcecentre.info/wp-content/uploads/2020/05/DataCollectionSOPCOVID-19.pdf (Accessed Sept 24, 2021)
19. Zulu JM, Ali J, Hallex K, et.al. Ethical challenges in research on post-abortion care with adolescents: experiences of researchers in Zambia. *Glob Bioeth* 2018; 31(1):104-119
20. World Health Organisation. Everybody business: strengthening health systems to improve health outcomes: WHO's framework for action. Geneva, World Health Organisation; 2007.

For peer review only - http://bmjopen.bmj.com/site/about/guidelines.xhtml

https://www.who.int/healthsystems/strategy/everybodys_business.pdf?ua=1 (Accessed Sept 24, 2020)

21. Elo S, Kyngäs H. The qualitative content analysis process. *J Adv Nurse* 2008;62:107-115.
22. UN Women, World Health Organisation. Violence Against Women and Girls. Data Collection during COVID-19. New York, UN Women; 2020 https://www.unwomen.org/-/media/headquarters/attachments/sections/library/publications/2020/vawg-data-collection-during-covid-19 (Accessed Sept 24 10, 2021)
23. World Health Organisation. Putting Women First: Ethical and Safety Recommendations for research on Domestic Violence Against Women. Geneva, World Health Organization;2001 https://apps.who.int/iris/bitstream/handle/10665/65893/WHO_FCH_GWH_01.1.pdf?sequence=1 (Accessed Sept 24, 2021)

For peer review only

Focused group topic guide for women

Demographic information (Note: Collect data on individuals before the FGD)

- Age (in completed years)
- Education (highest level attained)
- Residence (actual, urban, rural, slum)
- COVID-19 infection history (self, close family, work mates, friends)
- Social economic status - Employment status (i.e. formal or informal employment), income earning status and possibly income ranges as suitable in the country setting.

Knowledge about COVID-19 (such as cause, signs and symptoms)

1. What is COVID-19? What do you know about COVID-19?
   PROBE: for causes, transmission, signs, symptoms, sources of information about COVID-19
2. Did you perceive yourself to be at risk for COVID-19 infection?
   PROMPT: Probe for any special considerations (pregnancy, pre-existing health conditions, others).

Care seeking and perception of risk about being infected by COVID-19

3. The decision-making process of treatment seeking during COVID-19
   PROBE: Ask who influenced the decision to seek care during COVID-19 (self, partner, other family member, etc?)
4. Perception of risk of infection during SRH care seeking.
   PROMPT: How did this affect health seeking behaviour during COVID-19?
5. The treatment-seeking process during COVID-19
   a. Were your needs for accurate information on reproductive health services fulfilled? How? Or Why not? PROBE: information sources: brochures, social media, billboards, other printed media, information on service hours or location where services are offered etc.
   b. What were your needs for reproductive health services? Were these needs fulfilled?
      PROBE: Contraception? Abortion care? STI?
   c. Did you encounter any barriers in your bid to access needed reproductive health services? PROBES: transportation, behaviour of other persons, etc.

   PROMPT: Interviewer should probe for other context specific intermediaries to seeking care. E.g. paperwork for insurance
6. Do you think that reproductive services were readily available to clients who needed them? Specifically, the availability of the reproductive health medicine and medical supplies needed by clients based on the following:
   a. Availability of staff, medicines and supplies for SRH during COVID-19
   b. Schedule of treatments/appointments during COVID-19
      PROMPT: Probe for changes in the delivery of SRH services (specifically for family planning, abortion, post-abortion care and STI care) i.e. appointment-based system introduced.
   c. Interaction between the service provider and the patient was during COVID-19. PROBE: Interactions with different cadres of health workers. Nurses? Midwives? Doctors? Records? OPD staff? Etc.
   d. Was the interaction between service providers and staff described above different from pre-COVID-19 time? How?

7. What were some of the safety measures ensured by the health facility to protect clients from COVID-19 during services
8. Do you think that the COVID-19 had any impact on client protection and privacy during services?
   PROBE: What was done (or could have been) differently?

Post-pandemic recovery of the facilities to provide services in comparison to the pandemic period.

GENERAL COMMENTS: Based on the country situation, the probes for the questions above can be extended to cover the post-pandemic period if applicable.

For peer review only

Focused group topic guide for men

Demographic information

- Age (in completed years)
- Education (highest level attained)
- Residence (actual, urban, rural, slum)
- COVID-19 infection history (self, close family, work mates, friends)
- Social economic status: Employment status (i.e. formal or informal employment), income earning status and possibly income ranges as suitable in the country setting.

Knowledge about COVID-19 (such as cause, signs and symptoms)

1. What is COVID-19? What do you know about COVID-19?
   PROBE: for causes, transmission, signs, symptoms, sources of information about COVID-19
2. Did you perceive yourself to be at risk for COVID-19 infection?
   PROMPT: Probe for any special considerations (pregnancy, pre-existing health conditions, others).

Care seeking and perception of risk about being infected by COVID-19

1. What role did you play in the decision-making process of treatment seeking during COVID-19?
   PROBE: final decision maker (man only, joint, woman only, other); stage of involvement (decision stage or care seeking stage).
2. The role of men in the treatment-seeking process during COVID-19
   a. What role did you play in seeking for accurate information on reproductive health services? Were your needs fulfilled? How? Or Why not?
      PROBE: information sources: brochures, social media, billboards, other printed media, etc. The role of men in looking for sexual reproductive health services (including contraception and abortion care) and whether the needs were fulfilled
   b. What role did you play in looking for reproductive health services? Were your needs for reproductive health services fulfilled?
      PROBE: Contraception? Abortion care? STI?
   c. Did you encounter any barriers in your bid to access needed reproductive health services? PROBES: transportation, behaviour of other persons, entry restrictions for men, etc.
      NOTE: Interviewer should probe for other context specific intermediaries to seeking care. E.g. paperwork for insurance
3. Do you think that reproductive services were readily available to clients who needed them? The availability of the reproductive health medicine and medical supplies needed by clients?
   a. The schedule of treatments/appointments during COVID-19?
4. How was the interaction between the service provider and you as the male partner during COVID-19?
   PROBE: Interactions with different cadres of health workers. Nurses? Midwives? Doctors? Records? OPD staff? Etc
5. Was the interaction between service providers and staff described above different from pre-COVID-19 time? How?

For peer review only

6. What were some of the safety measures ensured by the health facility to protect clients from COVID-19 during services?
   PROBE: Were male partners stopped from entering consulting and treatment rooms due to COVID-19?
7. Do you think that the COVID-19 had any impact on client protection and privacy during services?
8. **PROBE**: What was done (or could have been) differently? E.g. barriers in seeking SRH services during COVID

Post-pandemic recovery of the facilities to provide services in comparison to the pandemic period.

COMMENT: Based on the country situation, the probes for the questions above can be extended to cover the post-pandemic period if applicable.

For peer review only

In-depth Interview topic guide for women

1. Demographic information
    - Age
    - Ethnicity
    - Marital status
    - Socio-economic status
    - Occupation
    - Education status
    - Religion
    - Place of residence and housing condition
    - Obstetric characteristics (no. of pregnancies, parity)

2. Knowledge about COVID-19
    - Cause (sexual transmission)
        - How do you think COVID-19 virus is transmitted?
        - Do you think it can be transmitted sexually?
    - Signs and symptoms of COVID-19
        - What are the sign and symptoms of COVID-19?
        - If yes, can you mention some that you know?
    - Care-seeking for COVID-19 infection
        - Do you know what to do/who to contact/ where to go if you experience COVID-19 signs and systems?
        - If yes, can you explain?
    - Sources of information and trusted communication channels (v. rumors, misinformation) Experience of COVID-19 (direct / indirect)
        - Where did you get information about COVID-19?
        - Was the information you obtained accurate?
        - Can you give me an example of accurate information you obtained?
        - Were there any rumors and/or misinformation about COVID-19?
        - How did you know that this information was just rumors or misinformation?
        - Have you fund sources of information that you can trust?
        - How did you know which information sources to trust?
        - Have you been infected by COVID-19?
        - Do you know of anyone who has been infected? If yes, was it a close friend or family member?
    - Perception of risk about being infected by COVID-19
        - Do you think you are at risk of being infected by the COVID-19 virus?
        - If no, can you tell me why not?
        - Do you think you can be infected whilst seeking care?
    - Ways to prevent being infected by COVID-19 (probe at individual, household, community levels)
        - Do you know how to prevent infection from COVID-19?
        - What can individuals do? What can households do? What can communities do?
    - Responsibility to prevent COVID-19 (probe at individual, household, community levels, authorities)
        - Who is responsible for the prevention of COVID-19 infection? Do you think this

For peer review only

responsibility was fulfilled by individuals, households, communities and authorities? How?

- Religious / socio-cultural practices and influencers
  - Are there any religious / socio-cultural practices that are used to prevent COVID-19 infection?
  - Does participation in any religious / socio-cultural practices make you practice prevention of COVID-19 differently from what is promoted by health professionals?

3. Sexual and Reproductive Health
   - Decision-making and agency to act
     - Who makes the decisions related to your sexual and reproductive health needs (self, husband/partner or family member)?
     - Who is usually responsible for carrying out the decision?
   - Issues of access / utilization
     - Did you need to seek SRH services during COVID-19?
     - If yes, were you able to access the services? How did you access the services?
     - If no, what were the barriers? What did you do?
   - Challenges faced
     - Are there any other challenges you faced in seeking SRH services during COVID-19? What are they and how did you address/manage the challenges?

4. Contraceptive practices
   - Changes in practice (if any) due to COVID-19
     - Which method of contraception do you normally use?
     - Did you have to change your normal method of contraception during COVID-19? If yes, which method and what influenced the change?
     - If no, why didn't you change from your normal practice?
   - Is pregnancy being avoided / delayed due to COVID-19? If yes, why?
   - Access to / utilization of different methods of contraception (barriers / drivers)
   - Which methods of contraception were accessible to you during COVID-19?
   - Was there a difference in the methods of contraception accessible to you compared to before COVID-19?
   - What was causing the difference?
   - Challenges in access / utilization
   - Are there any other challenges you faced in accessing/utilizing contraception services during COVID-19?
   - If yes, what were these challenges and how did you cope with them?

5. Induced abortion
   - Perceptions about abortion at normal times
   - The role abortion plays in women's lives
     - Why do you think women seek abortion services?
     - Do you think abortion is an essential service for women? If yes, in what way?
   - Fear of stigma
     - How does family, community and society view women who seek or have sought abortion services?

For peer review only

- Does this stigma affect your decision whether to have an abortion?
- Does this stigma affect how you will seek abortion services? If yes, how?
- What do you think are the effects of this stigma on women who need abortion services?

- Inequities in access to safe abortion
  - Do you think safe abortion services are equally accessible to all women who need them?
- Perceptions about abortion during COVID-19
  - Would you choose to seek the abortion services during COVID-19, if you need one?
  - If no, why not? If yes, where and how would you access the service?

- Access to different methods of abortion (barriers / drivers)
  - Did you seek abortion services during COVID-19? If yes, which abortion methods were available for you (medical abortion or surgical abortion)?
  - What pushed you to have an abortion? If you were given a choice of methods, which one did you choose and why?
  - Did you face any barriers in seeking and accessing abortion services during COVID-19?
- Challenges in accessing / utilizing safe abortion services (inequities in access)
  - Are there any other challenges you faced in accessing/utilizing safe abortion services? If yes, what were the challenges and how did you address them?

- 9. Violence against women (*Interviewer should use the term that is used locally to describe VAW)*

- Formal and informal systems of support, and coping strategies for VAW
  - Have you or any woman you know experienced any form of VAW?
  - What are some of the systems of support available for women who have experienced VAW?
  - Do you know where and how to access this support?
  - What are some of the coping strategies women who have experience such violence use?
- Perceptions of safety in home and in community safety/increased risk during COVID-19
  - Do you think are safer or at increased risk of experiencing VAW at home or in the community during COVID-19?
  - What has made it safer/riskier for women during COVID-19?
- Women's expectations of services for gender-based violence
  - If you or any other woman experiences GBV during COVID-19, do you expect the care and support services to be available?
  - Do you know where and how to access these services?
  - Do you expect any barriers to accessing these services during COVID-19? If yes, what kind of barriers?
- What are additional stress factors (e.g. school-age children at home, older parents, loss of job/income, partner loss of job, etc.) that increase the likelihood of VAW?
- How does stress affect frequency and severity of violence, particularly during COVID-19?

For peer review only

6. Sexually transmitted infections *(interviewer should use the locally known terms for STIs and the specific diseases)*
   - Perceptions about STI
     - Is anybody at risk of getting a STI? Why or why not?
     - How are people who are known to have a STI perceived in the community?
   - Experience of STI during COVID-19
     - Have you required care and treatment for a STI during COVID-19? If yes, where and how did you seek this service?
     - Were there any barriers to accessing this service? If yes, which ones and what did you do?
     - If no, would you seek care and treatment if required during COVID-19?
     - If no, why not? If yes, where and how would you access this service?

7. Psycho-social
   - Psychosocial issues associated with being infected with COVID-19 (probe: more significant for women/men, more significant for pregnant women)
     - Did you experience any stress, anxiety or depression during COVID-19? If yes, at what level (mild, moderate, severe)?
     - What do you think led to the psychosocial issues you experienced? Was it due to being infected or other related issues or both?
     - Did your partner also experience any psychosocial issues during COVID-19?
     - Who was most affected, yourself or your partner?

8. Final questions
   - On-going concerns about COVID-19 and its possible impact
     - Are there any other concerns about COVID-19 and its impact on you, your family or in general that you wish to express?
   - Needs related to accurate information (probe further information needs – NB consider how to provide information if necessary)
     - Was accurate information readily available and accessible during COVID-19?
     - Was it easy to understand?
     - Was the information that was available adequate?
     - Was there additional information you would have liked to obtain but was missing?
     - Are there better ways of making accurate information accessible to you and others?
   - Needs related to sexual reproductive health services (probe further SRH needs – NB consider how to support/provide if necessary)
   - Do you think SRH services were responsive to your needs during COVID-19?
   - If no, which of your needs were not met?
   - Which SRH services are most important for you, that should be available even in health emergencies like COVID-19?
   - How do you think provision of SRH services can be supported during other health emergencies like COVID-19?

For peer review only

Additional themes will be added as appropriate for each target group and questions revised accordingly (with attention to first person / third person views), and across the three phases of the study.

For peer review only

In-depth Interview topic guide for men

1. Demographic information
   - Age
   - Ethnicity
   - Marital status
   - Socio-economic status
   - Occupation
   - Education status
   - Religion
   - Place of residence and housing condition

2. Knowledge about COVID-19
   - Cause (sexual transmission)
     - How do you think COVID-19 virus is transmitted?
     - Do you think it can be transmitted sexually?
   - Signs and symptoms of COVID-19
     - What are the sign and symptoms of COVID-19?
     - If yes, can you mention some that you know?
   - Care-seeking for COVID-19 infection
     - Do you know what to do/who to contact/ where to go if you experience COVID-19 signs and systems?
     - If yes, can you explain?
   - Sources of information and trusted communication channels (v. rumors, misinformation) Experience of COVID-19 (direct / indirect)
     - Where did you get information about COVID-19?
     - Was the information you obtained accurate?
     - Can you give me an example of accurate information you obtained?
     - Were there any rumors and/or misinformation about COVID-19?
     - How did you know that this information was just rumors or misinformation?
     - Have you fund sources of information that you can trust?
     - How did you know which information sources to trust?
     - Have you been infected by COVID-19?
     - Do you know of anyone who has been infected? If yes, was it a close friend or family member?
   - Perception of risk about being infected by COVID-19
     - Do you think you are at risk of being infected by the COVID-19 virus?
     - If no, can you tell me why not?
     - Do you think you can be infected whilst seeking care?
   - Ways to prevent being infected by COVID-19 (probe at individual, household, community levels)
     - Do you know how to prevent infection from COVID-19?
     - What can individuals do? What can households do? What can communities do?
   - Responsibility to prevent COVID-19 (probe at individual, household, community levels, authorities)
     - Who is responsible for the prevention of COVID-19 infection? Do you think this responsibility was fulfilled by individuals, households, communities and

For peer review only

authorities? How?

- Religious / socio-cultural practices and influencers
  - Are there any religious / socio-cultural practices that are used to prevent COVID-19 infection?
  - Does participation in any religious / socio-cultural practices make you practice prevention of COVID-19 differently from what is promoted by health professionals?

3. Sexual and Reproductive Health
   - Decision-making and agency to act
     - Who makes the decisions related to the sexual and reproductive health needs of your partner (you, partner or family member)?
     - Who is usually responsible for carrying out the decision?
   - Issues of access / utilization
     - Did your partner need to seek SRH services during COVID-19?
     - If yes, was your partner able to access the services? How did your partner access the services?
     - If no, what were the barriers? What did you and/or your partner do?
     - What role did you play in ensuring your partner sought and/or accessed the needed SRH services?
   - Challenges faced
     - Are there any other challenges your partner faced in seeking SRH services during COVID-19? What are they and how did you and/or your partner address/manage the challenges?

4. Contraceptive practices
   - Changes in practice (if any) due to COVID-19
     - Which method of contraception do you and/or your partner normally use?
     - Did you have to change your normal method of contraception during COVID-19? If yes, which method and what influenced the change?
     - If no, why didn't you change from your normal practice?
   - Did your partner avoid / delay pregnancy due to COVID-19? If yes, why?
   - Access to / utilization of different methods of contraception (barriers / drivers)
   - Which methods of contraception were accessible to you and your partner during COVID-19?
   - Was there a difference in the methods of contraception accessible to you compared to before COVID-19?
   - What was causing the difference?
   - Challenges in access / utilization
   - Are there any other challenges you and your partner faced in accessing/utilizing contraception services during COVID-19?
   - If yes, what were these challenges and how did you cope with them?
   - What role did you play in ensuring your partner sought and/or accessed contraception services?

5. Induced abortion

For peer review only

- Perceptions about abortion at normal times
- The role abortion plays in women's lives
  - Why do you think women seek abortion services?
  - Do you think abortion is an essential service for women? If yes, in what way?
- Fear of stigma
  - How does family, community and society view women who seek or have sought abortion services?
  - Does this stigma affect your decision whether to have an abortion?
  - Does this stigma affect how you will seek abortion services? If yes, how?
  - What do you think are the effects of this stigma on women who need abortion services?
- Inequities in access to safe abortion
  - Do you think safe abortion services are equally accessible to all women who need them?
- Perceptions about abortion during COVID-19
  - Would you choose to seek the abortion services during COVID-19, if your partner needed one?
  - If no, why not? If yes, where and how would you access the service?
- Access to different methods of abortion (barriers / drivers)
  - Did your partner seek abortion services during COVID-19? If yes, which abortion methods were available (medical abortion or surgical abortion)?
  - What pushed you and your partner to have an abortion? If you were given a choice of methods, which one did you choose and why?
  - Were there any barriers in seeking and accessing abortion services during COVID-19?
- Challenges in accessing / utilizing safe abortion services (inequities in access)
  - Are there any other challenges you faced in accessing/utilizing safe abortion services? If yes, what were the challenges and how did you address them?
- What was your role in ensuring your partner accessed the abortion service?

6. Violence against women (*Interviewer should use the term that is used locally to describe VAW)*
   - Formal and informal systems of support, and coping strategies for VAW
     - Has your partner or any woman you know experienced any form of VAW?
     - What are some of the systems of support available for women who have experienced VAW?
     - Do you know where and how to access this support?
     - What are some of the coping strategies that women who have experienced such violence use?
   - Perceptions of safety in home and in community safety/increased risk during COVID-19
     - Do you think women are safer or at increased risk of experiencing VAW at home or in the community during COVID-19?
     - What has made it safer/riskier for women during COVID-19?
   - Women's expectations of services for gender-based violence

For peer review only

- If your partner experiences GBV during COVID-19, do you expect the care and support services to be available?
- Do you know where and how to access these services?
- Do you expect any barriers to accessing these services during COVID-19? If yes, what kind of barriers?
- What do you perceive as your role in ensuring your partner get the care and support needed?

- What are additional stress factors (e.g. school-age children at home, older parents, loss of job/income, partner loss of job, etc.) that increase the likelihood of VAW?
- How does stress affect frequency and severity of violence, particularly during COVID-19?

6. Sexually transmitted infections *(interviewer should use the locally known terms for STIs and the specific diseases)*
   - Perceptions about STI
     - Is anybody at risk of getting a STI? Why or why not?
     - How are people who are known to have a STI perceived in the community?
   - Experience of STI during COVID-19
     - Has your partner required care and treatment for a STI during COVID-19? If yes, where and how did you seek this service?
     - Were there any barriers to accessing this service? If yes, which ones and what did you do?
     - If no, would you seek care and treatment if required during COVID-19?
     - If no, why not? If yes, where and how would you access this service?
     - What was your role in ensuring your partner accessed the care and treatment?

7. Psycho-social
   - Psychosocial issues associated with being infected with COVID-19 (probe: more significant for women/men, more significant for pregnant women)
     - Did you experience any stress, anxiety or depression during COVID-19? If yes, at what level (mild, moderate, severe)?
     - What do you think led to the psychosocial issues you experienced? Was it due to being infected or other related issues or both?
     - Did your partner also experience any psychosocial issues during COVID-19?
     - Who was most affected, yourself or your partner?

8. Final questions
   - On-going concerns about COVID-19 and its possible impact
     - Are there any other concerns about COVID-19 and its impact on you, your family or in general that you wish to express?
   - Needs related to accurate information (probe further information needs – NB consider how to provide information if necessary)
     - Was accurate information readily available and accessible during COVID-19?
     - Was it easy to understand?
     - Was the information that was available adequate?
     - Was there additional information you would have liked to obtain but was

For peer review only

missing?
- Are there better ways of making accurate information accessible to you and others?

- Needs related to sexual reproductive health services (probe further SRH needs – NB consider how to support/provide if necessary)
- Do you think SRH services were responsive to your needs during COVID-19?
- If no, which of your needs were not met?
- Which SRH services are most important for you, that should be available even in health emergencies like COVID-19?
- How do you think provision of SRH services can be supported during other health emergencies like COVID-19?

Additional themes will be added as appropriate for each target group and questions revised accordingly (with attention to first person / third person views), and across the three phases of the study.

For peer review only

In-depth Interview topic guide for SRH healthcare providers

Demographic information

- Age
- Ethnicity
- Marital status
- Socio-economic status
- Occupation
- Education status
- Religion

Health facility service availability and readiness

Health service delivery

1. Mobilization of resources for the diagnosis and the treatment of COVID-19 patients by the *(insert Country name*) government happened during COVID-19. Did the mobilization involve your facility? How was the facility involved? (Probe community, regional or national level involvement)

   COMMENT: *Indicate if facility was a designated COVID-19 treatment Centre*
2. What was the emergency response plan of your facility during the outbreak?
3. How did the mobilization of resources towards COVID-19 patients impact the service delivery in your facility, including the impact on human resources, space, medical supplies, etc.?
4. Did the mobilization of resources negatively or positively impact service delivery in your facility? Elaborate
5. Was there a set of essential Sexual & Reproductive Health services outlined to be maintained during the lockdown phase of the COVID-19 pandemic?
   PROMPT: Ask for each of the following services – family planning, abortion, post-abortion care, care and support for GBV and STI prevention and treatment
6. Were there disruptions to any of the SRH essential services outlined? What was the nature (services suspended, limited or shifted) of disruptions observed for each of these essential services (if any)?
   PROBES: for causes (lack of personnel, equipment, medicines or reduced demand, physical space), changes in usual service provision (introduction of appointment-based systems, telemedicine, shifting of usual location) and level of disruption (partial/ complete/not disrupted)
7. Do you think the delivery of SRH services during COVID-19 adequately responded to the needs of the clients at that time?
   PROBE: in what way?
8. What has the recovery process been like and what was the turning point?
   NOTE: Only for applicable countries.
   PROBE for coping mechanisms or approaches to ensuring SRH service delivery.

Health workforce （Human resources）

9. How did COVID-19 affect the health status of health workforce, such as doctors, nurses, ambulance drivers and hospital managers etc.? What measures were taken to protect them from the effects of the epidemic?

PROBE: for the provision of PPE, type of PPE, adequacy of supply and frequency of supply.

10. Was there any form of training on COVID-19? PROMPT: Probe for details of the training if provided – What areas of COVID-19? What form did this training take? Who received this training?
11. Were there employees infected? If someone was infected, how did the infection happen? What was the impact on service delivery due to the infection of SRH workers? How did it influence the service delivery?

COMMENT: *In facilities where SRH staff also provide general care in health facility, ask for the impact of an infected clinical staff in the facility even if not SRH-specific*

12. While knowing that they were at higher risk of contracting the virus, did female workers still have to fulfil the obligations such as caring for elders and immediate family (such as children or young dependents)? Is there any other conflict or challenge they faced during COVID-19? Note: Probe for any challenge worth considering in current setting.

Health information system

13. How does the reporting system for infectious diseases run?
    PROMPT: Probe for reporting system for COVID-19.
14. How did this system influence the availability of services in SRH facilities?
15. How did you keep information about the COVID-19 and SRH services flowing during the outbreak with your upper-level medical institutions and potential clients in your community?
    PROBE: how were changes in the delivery of SRH services communicated to clients? How was important COVID-19 information communicated to clients?

Access to essential medicines

16. Was the access to essential medicines, including regular medicines such as birth control pills, contraceptive methods impacted because of the lockdown/shutdown of the city during the COVID-19?

COMMENT*: Shutdown can be replaced with lockdown/restrictions depending on what the COVID-19 response was in country using this guide*

PROBE: ask about medicines for all the study focus SRH services

17. How has the outbreak affected the access to essential medicines in SRH for the patients and the general population?

PROBE: for each- contraceptive methods, abortion, post abortion care, STI prevention and treatment and GBV care and support

COMMENT: Probe separately for routine medication (such as antibiotics, analgesics etc where applicable)

Health systems financing

18. How did the COVID-19 epidemic affect the availability of funds at your facility?
    PROMPT: Inquire about effect on the financing and maintenance of essential SRH services and possible disruptions due to the pandemic, availability of additional government funding allocated to assuring essential health services.

For peer review only

COMMENT: *This section should be informed by the existing health financing policy (i.e. National health insurance, existing SRH related exemptions and waiver systems) or COVID-19 specific directives on financing.*

Leadership and governance

19. What are your perceptions on the roles and responsibilities of providing SRH services during outbreaks like COVID-19?
PROBE: where does the primary responsibility to ensure continuity of services lie? Who are the enablers, implementers?
20. How did your facility respond to the emergency and manage to ensure the SRH-related service delivery in your facility?
PROMPT: Probe for country-specific plan, policies for maintaining SRH service provision during the pandemic.
PROBE: separately for, family planning, STI and abortion services, services prioritized by the facility during the pandemic.

Health service provider perceptions on the delivery of specific SRH services in the context of COVID-19

A. Gender based Violence

Perceptions of changes in the nature and magnitude of violence against women, and especially domestic violence during COVID-19

- Have you noticed any change in the number of women coming into the health facility reporting gender-based violence since the COVID-19 crisis began?
  - Follow up: Increase or decrease?
  - Follow up: Have the women coming in who report experiencing violence mostly been returning patients, or mostly been new patients?
  - Follow up: Have you noticed an increase in the frequency of abuse that women are experiencing since movement restrictions were put in place?
  - If possible, specify types of violence (physical, sexual, psychological by a partner), non-partner sexual abuse, other

- Do you think that the violence women have been experiencing has become more severe during the crisis, for example has there been an increase in injuries? Violence during pregnancy?
  - Follow up: Have the types of violence that women are reporting changed? How so?

Nature of service provision during COVID-19, whether services for VAW are being changed or adapted and if safety considerations are adequately addressed?

- Since the start of COVID-19, has your health facility changed or adapted services for survivors 'of violence?
  - Probe: (if not mentioned) In some health centers, providers have maintained 'case management' using video calls, or text messages. Did your health center make use of any new technologies to maintain services? If so, did you develop a protocol to ensure safety of survivors?

For peer review only

Access to /use of health services for VAW and related challenges

- Some health centers have had to deal with shortages of personal protective equipment and medications that have impacted their ability to offer services during the COVID-19 crisis. Have there been any new barriers to delivering services to survivors at your health facility?
    - What are these barriers and what approaches are being used to overcome these barriers?

- In a lot of places, external referral systems have also been impacted by the crisis, such as lower numbers of available spaces in shelters due to the need for physical distancing, or NGOs that have been temporarily shut down. To your knowledge, have any changes have been made to your external referral systems? What are these?
    - Follow up: Are any of the services to which you typically refer survivors available in a more limited capacity, or no longer available due to the crisis?

Attitudes towards provision of SRH services in the context of COVID-19

- Do you think it is essential to provide services for VAW during COVID-19? Why do you think so?

B. Induced abortion

Perceptions of changes in the demand for abortion during COVID-19.

- Have you noticed any change in the number of women seeking abortion services at your facility during COVID-19?
    - Follow up: Increase or decrease?
    - Follow-up: any changes in the abortion methods requested?
    - What do you think were the factors driving this change?

Delivery of safe abortion services during COVID-19?

- Where and how can people seek contraceptive services during COVID-19?
    - How many methods of abortion were available in your health facility during COVID-19? Which method of abortion did you suggest during COVID-19? Why?
    - What challenges did you meet in providing safe abortion services during COVID-19? What approaches did you use to overcome these challenges?

Attitudes towards provision of SRH services in the context of COVID-19

- Do you think it is essential to provide safe abortion services during COVID-19? Why do you think so?

C. Contraceptive services

Perceptions of changes in the demand for contraception services during COVID-19.

- Have you noticed any change in the number of women seeking contraception services at your facility during COVID-19?
    - Follow up: Increase or decrease?
    - Follow-up: any changes in the contraception methods requested?
    - What do you think were the factor driving this change?

Delivery of contraception services during COVID-19?

- Where and how can people seek contraceptive services during COVID-19?

- How many kinds of contraceptive methods were available in your health facility during COVID-19?
- What are the new/ altered ways by which contraceptive services are provided as a response to COVID-19?
- What challenges did you meet in providing contraceptive services during COVID-19? What approaches did you use to overcome these challenges?

Attitudes towards provision of SRH services in the context of COVID-19

- Do you think it is essential to provide contraception services during COVID-19? Why do you think so?

D. <u>Prevention and treatment for sexually transmitted infections</u>

Perceptions of changes in the demand for care and treatment for STI during COVID-19.

- Have you noticed any change in the number of women seeking care and treatment for STI at your facility during COVID-19?
    - Follow up: Increase or decrease?
    - What do you think were the factor driving this change?

Availability of STI care and treatment during COVID-19

- Where and how can people seek care and treatment for STI during COVID-19?
  - What are the new/ altered ways by which care and treatment for STI services are provided as a response to COVID-19?
  - Were all the medicines and supplies for providing the care and treatment available during COVID-19?
  - What challenges did you meet in providing care and treatment for STI services during COVID-19? What approaches did you use to overcome these challenges?

Attitudes towards provision of SRH services in the context of COVID-19

- Do you think it is essential to provide prevention and treatment for STIs during COVID-19? Why do you think so?

3. Perceived psychosocial effects of COVID-19
    - Did you experience stress/anxiety/depression during COVID-19?
    - How would rate your psychosocial health?
    - What role did COVID-19 play in influencing your psychosocial health?
    - In what way?
    - What do you think is the psychosocial impact of COVID-19 on people?
    - Do you think the impact was the same for men and women? If yes, why?
    - If no, in what ways is the psychosocial impact different?
    - What do you think is the psychosocial impact of COVID-19 on families?
    - What do you think is the psychosocial impact of COVID-19 on communities?

4. Final questions
    - On-going concerns about COVID-19 and its possible impact
        - Are there any other concerns about COVID-19 and its impact on you, your family or in general that you wish to express?
    - Needs related to accurate information (probe further information needs – NB consider how to provide information if necessary)

For peer review only

- Was accurate information readily available and accessible during COVID-19?
- Was it easy to understand?
- Was the information that was available adequate?
- Was there additional information you would have liked to obtain but was missing?
- Are there better ways of making accurate information accessible to you and others?

- Needs related to sexual reproductive health services (probe further SRH needs – NB consider how to support/provide if necessary)
- Do you think SRH services were responsive to the needs of patients during COVID-19?
- If no, which of needs were not met?
- Which SRH services are most important, that should be available even in health emergencies like COVID-19?
- How do you think provision of SRH services can be supported during other health emergencies like COVID-19? E.g. barriers in seeking SRH services during COVID

Additional themes will be added as appropriate for each target group and questions revised accordingly (with attention to first person / third person views), and across the three phases of the study.

For peer review only

BMJ Open

Health systems analysis and evaluation of the barriers to availability, utilization and readiness of sexual and reproductive health services in COVID-19 affected areas: A WHO mixed methods study protocol

Journal:	*BMJ Open*
Manuscript ID	bmjopen-2021-057810.R1
Article Type:	Protocol
Date Submitted by the Author:	25-Jan-2022
Complete List of Authors:	Kouanda, Seni; Institut de Recherche en Sciences de la Sante; Institut Africain de Sante Publique<br>Nahyuha Chomi, Eunice; Research Institute of Health Sciences<br>Kim, Caron; World Health Organization, Department of Sexual and Reproductive Health and Research. Preventing Unsafe Abortion Team<br>Jen, Sothornwit; Khon Kaen University, Department of Obstetrics and Gynaecology<br>Bahamondes, Luis; State University of Campinas – UNICAMP, Obst & Gynecol<br>Cecatti, Jose; University of Campinas, Obstetrics and Gynecology<br>Lumbiganon, Pisake; Khon Kaen University, Faculty of Medicine<br>Emefa, Modey; University of Ghana<br>Brizuela, Vanessa; WHO, Department of Reproductive Health and Research<br>Kuganantham, Hamsadvani; World Health Organization, Department of Reproductive Health and Research<br>Ali, Moazzam; Organisation mondiale de la Sante,
**Primary Subject Heading**:	Public health
Secondary Subject Heading:	Health policy, Health services research, Infectious diseases
Keywords:	COVID-19, PUBLIC HEALTH, Reproductive medicine < GYNAECOLOGY

SCHOLARONE™
Manuscripts

Manuscript Protocol

Health systems analysis and evaluation of the barriers to availability, utilization and readiness of sexual and reproductive health services in COVID-19 affected areas: A WHO mixed methods study protocol

- Kouanda Seni-Reproductive Health Unit, Institute for Research in Health Sciences, Ouagadougou, Burkina Faso
- Chomi Eunice- Reproductive Health Unit, Institute for Research in Health Sciences, Ouagadougou, Burkina Faso
- Kim Caron- Department of Reproductive Health and Research, World Health Organization, Avenue Appia 20, 1211 Genève 27, Switzerland
- Sothornwit Jen- Department of Obstetrics and Gynaecology, Faculty of Medicine, Khon Kaen University, Khon Kaen, Thailand.
- Bahamondes Luis- School of Medical Sciences, University of Campinas (UNICAMP), Campinas, Brazil
- Cecatti Jose Guilherme- School of Medical Sciences, University of Campinas (UNICAMP), Campinas, Brazil
- Lumbiganin Pisake- Faculty of Medicine, Khon Kaen University, Khon Kaen, Thailand
- Modey Emefa-Department of population, family and Reproductive Health, University of Ghana, Legon-Accra, Ghana.
- Brizuela Vanessa- Department of Reproductive Health and Research, World Health Organization, Avenue Appia 20, 1211 Genève 27, Switzerland
- Kugananthan Hamsa- Department of Reproductive Health and Research, World Health Organization, Avenue Appia 20, 1211 Genève 27, Switzerland
- Ali, Moazzam* on behalf of WHO HRP social Science Research Team**- Department of Reproductive Health and Research, World Health Organization, Avenue Appia 20, 1211 Genève 27, Switzerland

*Corresponding author: Moazzam Ali (alimoa@who.int)
Department of Reproductive Health and Research, World Health Organization, Avenue Appia 20, 1211 Genève 27, Switzerland

**** WHO HRP Social Science Research Team**: **Brazil** (Luis Bahamondes, Jose Guilherme Cecatti); **Burkina Faso** (Eunice Chomi, Seni Kouanda); **China** (Kun Tang, Hanxiyue Zhang, Yifan Zhu, Yueping Guo, Ge Yang and Chunxiao Peng); **Ghana** (Deda Ogum Alangea, Kwasi Tropsey, Emefa Judith Modey); **Pakistan** (Rozina Karmaliani, Laila Ladak); **Thailand** (Pisake Lumbiganon, Jen Sothornwit); **Kenya** (Marleen Temmerman, Abdu Mohiddin, Ferdinand Okwaro); **Italy** (Massimo Mirandola, Maddalena Cordioli, Alessia Savoldi, Simone Garzon, Stefano Uccella, Ranieri Poli.) **UK** (Nigel Sherriff, Alexandra Sawyer, Jorg Huber, Jaime Vera, Debbie Williams).
WHO Secretariat (Moazzam Ali, Caron Kim, Hamsadvani Kuganantham, Igor Toskin, Vanessa Brizuela, Anna Thorson, Joy Jerop Chebet, Hugo Gamerro Abrego , Soe Soe Thwin, Mohammad Ali)

For peer review only

Word count (excluding title page, abstract, references, figures and tables)=4163

KEYWORDS
Contraception, sexual and reproductive health, comprehensive abortion services, COVID-19, sexual and gender-based violence

For peer review only

Abstract

Introduction The novel Corona Virus Disease of 2019 (COVID-19) has led to an unprecedented increase in demand on health systems to care for people infected, necessitating the allocation of significant resources, especially medical resources, towards the response. This, compounded by the restrictions on movement instituted may have led to disruptions in the provision of essential services, including sexual and reproductive health (SRH) services. This study aims to assess the availability of contraception, comprehensive abortion care, sexually transmitted infection (STI) prevention and treatment and sexual and gender-based violence (SGBV) care and support services in local health facilities during COVID-19 pandemic. This is a standardized generic protocol designed for use across different global settings.

Methods and Analysis This study adopts both quantitative and qualitative methods to assess health facilities' SRH service availability and readiness, and clients' and providers' perceptions of the availability and readiness of these services in COVID-19-affected areas. The study has 2 levels: (1) Perceptions of clients (and their partners) and healthcare providers, using qualitative methods, and (2) assessment of infrastructure availability and readiness to provide SRH services through reviews, facility service statistics for clients and a qualitative survey for healthcare provider perspectives. The health system assessment will use a cross-sectional panel survey design with two data collection points to capture changes in SRH services availability as a result of the COVID-19 epidemic. Data will be collected using focus group discussions, in-depth interviews and a health facility assessment survey.

Ethics and Dissemination Ethical approval for this study was obtained from the World Health Organisation (WHO) Scientific and Ethics Review Committee (protocol ID CERC.0103). Each study site is required to obtain the necessary ethical and regulatory approvals that are required in each specific country.

Strengths and limitation of this study

- The study approach and design with mixed method (qualitative and quantitative) enable a comprehensive analysis of sexual and reproductive health in the context of COVID19 .
- The inclusion of partners will enhance understanding of gender dynamics and support efforts towards identifying strategies.
- A possible limitation is that the study will be conducted during and after one of the waves of the pandemic, at which stage there may have been changes in the health system as lessons are learnt.
- Another limitation is that the estimation of the recovery period may be difficult in settings where the pandemic is yet to be brought under control.

For peer review only - http://bmjopen.bmj.com/site/about/guidelines.xhtml

INTRODUCTION

The novel COVID-19 disease, which was first reported in December 2019 and rapidly spread globally, has led to an unprecedented increase in demand on health systems to care for people infected, necessitating the allocation of significant resources, especially medical resources, towards the response. The increased demand, compounded by the restrictions on movement instituted as part of containment measures may have led to disruptions in the provision of essential services, including sexual and reproductive health (SRH) services [1,2].

The novel coronavirus is new to humans and only limited scientific evidence is available on the impact of COVID-19 on SRH service delivery. However, lessons from the Ebola and Zika virus outbreaks have highlighted the severe disruptions in SRH services that expose women and girls in particular to preventable health risks [2,3]. Some services may be unavailable due to either facilities and health workers being repurposed to care for COVID-19 patients, patient safety concerns, movement restrictions disrupting travel to hospitals, supply chain disruptions or a reduction in health workers because of increasing numbers being themselves infected by COVID-19 [4,5]. In addition, overwhelmed with COVID-19 cases, clinical staff may not have the time or personal protective equipment (PPE) needed to provide family planning counselling and commodities [5]. Recent evidence suggests disruptions lasting 3-6 months in 2020 left between 4-23 million women in low- and middle-income countries unable to access modern contraceptives, a projected 1.4 million (500,000-2.7 million) unintended pregnancies and an additional 31 million cases of secual and gender-based violence (SGBV) [1, 6-9]. Furthermore, studies have modelled the potential impact, showing that even a 10% reduction in essential SRH services could lead to an estimated 15 million unintended pregnancies, 3.3 million unsafe abortions and 29, 000 additional maternal deaths during the next 12 months [7-10]. Continuity of essential health services while keeping people safe during the response to disease outbreaks such as the COVID-19 pandemic is therefore essential for the prevention of both direct and indirect mortality [4,5].

This is a generic standardized protocol designed to maximize the likelihood that data are systematically collected and shared rapidly in a format that can be easily reproducible, aggregated, tabulated and analyzed across many different settings globally and be useful as templates for use in health emergencies in the future. This will facilitate the comparison of results across regions and countries and will potentially improve the quality of observational studies by identifying and minimizing biases. Given that use in different settings will require some adaptation, these possibilities have been highlighted throughout the protocol.

The introduction should be updated with country specific data on COVID-19 epidemiology and current research findings prior to submission to local/country national institutional review boards

For peer review only - http://bmjopen.bmj.com/site/about/guidelines.xhtml

The main aim of this study is to assess the availability of contraception, comprehensive abortion care, STI prevention and treatment (including HIV) and SGBV care and support services in local health facilities during COVID-19 pandemic. The four specific objectives of the study are:

1. To explore the availability of, and health facility readiness to provide these services in areas most affected by COVID-19.
2. To assess the availability and quality of services and barriers to the utilization of these services from clients' and providers' perspectives in the selected COVID-19 affected areas.
3. To assess the post-pandemic recovery* of the facilities in the provision of these services in comparison to the pandemic period.
4. To enhance the SRH service capacity in COVID-19 through advocacy, policy briefs, media dissemination and academic papers towards national and regional stakeholders including policymakers, academia, healthcare providers and the community.

**Post-pandemic recovery refers to the period when ideally health facilities have been able to recover from the disruption in service provision, following reduced transmission levels. Caution should be applied to the term "post-pandemic" as services may be still impacted even with lower levels of transmission. Therefore, the research team should be cognizant of the distinct time periods in their facility assessments.*

METHODS AND ANALYSIS

Study design

This is a repeated cross-sectional study, using both quantitative and qualitative methods. The two data collection points, baseline and end-line, will be 9 to 12-months apart. The aim of having two data collection points is to document and share the local evidence with authorities on the SRH services at baseline, and then at end line to track and demonstrate the changes and improvement in services over time. The World Health Organisation (WHO) situational level assessment of COVID-19 transmission will be used as the basis for determining when to conduct baseline and endpoint data collection [11]:

- Level 0: no known transmission in the preceding 28 days, no restriction on daily activities.
- Level 1: basic measures in place, clusters of cases reported controlled with basic measures, limited and transient disease.
- Level 2: local transmission from imported cases to close contacts. Contact tracing, physical distancing and quarantine measures can contain the spread.
- Level 3: community transmission, where source of infection is untraceable, outbreak rapidly spreads in clusters.
- Level 4: disease outbreak has become an epidemic, where there are major clusters of infection all over the country, high number of deaths and it is very difficult to control transmission without strict containment measures.

Countries should time the baseline and end-line data collection as follows: Baseline data collection is rproposed when countries are experiencing level 3 or 4 transmission (referred to here as peak transmission/pandemic period), as disruptions in service provision are likely to occur during these periods. End-line data collection is proposed when countries have tentatively

For peer review only - http://bmjopen.bmj.com/site/about/guidelines.xhtml

managed to contain the spread and are at levels 0,1 or 2 (referred to here as post-pandemic period/after the pandemic) after experiencing levels 3 or 4.
Given the dynamic nature of the epidemic, where each country is experiencing varying levels of COVID-19 transmission (and the challenges from the resurgence of variants and sudden increase in the number of cases), it is difficult to assume standardized pandemic conditions at country level. Therefore, the time interval between data collection points is just an estimation. For countries that have contained the spread, it may be decided to collect baseline data retrospectively, selecting the peak period. However, strategies to minimize or avoid recall bias should be used.

Study Setting
The study will be conducted in geographical areas selected based on the epidemic status. To get variation in responses, health facilities will be selected as focal points considering their qualification to provide SRH services and other criteria (see section 3.4) for the researchers to access community members for participation in the study. It is expected that variation in health facilities distributed within different geographical areas will provide access to communities of all socioeconomic backgrounds.

Due to various local realities and political factors, countries should select the research study sites based on geographical location, organization of SRH service delivery and epidemic status where COVID-19 is likely to have significantly affected service delivery (given that even within countries the transmission status differs, hence the differential impact on facilities in different geographical areas). Consultation with the Ministry of Health will help identify the areas most affected by COVID-19.

Study Population
The study population will be women seeking SRH services from the selected health facilities and their partners who accompany them, as applicable, and healthcare providers working there.

In contexts where it is not customary for men to accompany their partners when they seek SRH services, other options can be used to access them. Healthcare providers can be medical doctors, nurses, midwives, nurse assistants, allied health professionals and other cadres depending on the norms and standards for the provision of SRH services in different country contexts.

Sample selection
Each sample will be selected based on specific criteria, as well as specific country contextual factors.

For peer review only - http://bmjopen.bmj.com/site/about/guidelines.xhtml

Health facility selection

The minimum criteria for selection should include availability of human resources, primarily the qualification to provide contraception, safe abortion, including the treatment of complications and the provision of post-abortion care, STI care and treatment and support for women experiencing SGBV. In addition, the diversity of hospital capacity, administrative rank, urban/rural setting and the willingness of the providers in charge to participate in the study, as well as how the COVID-19 response has been organized in terms of treatment centers. These criteria for selection of health facilities within the geographical sites were made to encourage a representative mix of facilities. The same health facilities will be used for both data collection points to highlight the changes in SRH delivery due to COVID-19 and post-pandemic recovery.

Given the dynamic nature of the pandemic *some flexibility should be given to all study sites in the selection of facilities, maintaining the minimum criteria specified above*

Qualitative sample

Selection of women

Women will be recruited for in-depth interviews (IDIs) and focus group discussions (FGDs). The women will be purposively selected to obtain a sample of women and their partners. Selection will be based on 1) being of reproductive age (18-49) and 2) having sought or tried to receive SRH services from local health facilities. The study will use gatekeepers, who will be healthcare provides not involved in the study to approach the women as they leave the hospital. Their role will be limited to the identification and introduction of potential participants to the study team.

For the IDIs, a sample of 10~15 women (or until saturation) and 6~12 partners (or until saturation) will be recruited. For countries with more enrolled hospitals in the study, the maximum number of the IDIs will be with 10 women (or until saturation) at reproductive age seeking for reproductive healthcare services, and with 5 partners (or until saturation) per hospital. This will be done as an exit interview and the women will be consecutively selected until the desired sample (or saturation) has been achieved.

For FGDs, gatekeepers will also ask participants about their level of comfort discussing experience seeking SRH issues in a group before being recruited. About 6-8 participants per FGD will be recruited, with the expectation of at least two focus groups per facility. Some female and male participants will also be invited for individual interviews after the FGDs.

The ages can be adjusted to account for differences in the nationally recognised reproductive age group in different contexts. Inclusion of girls younger than 18 years should take into consideration ethical issues of assent and consent.

To adjust to challenges posed by COVID-19 transmission, additional participants can be recruited through online recruitment from chat groups such as Facebook, Twitter or WhatsApp. Careful attention must be paid to the ethical issues of privacy and confidentiality of participants and

For peer review only - http://bmjopen.bmj.com/site/about/guidelines.xhtml

integrity of the researchers (transparency of aims, details, risks and benefits of the study, obtaining the necessary permission to join restricted groups and the presence of the researcher in the group). Other challenges of online recruitment that need attention include retention of participants, potential selection bias, verification of participant identity and comprehension of informed consent [12]. Care needs to be taken by hiding the names and faces (e.g., via video conference calls) of the online participants, responsible handling of participants' personal information to minimize the likelihood of embarrassment, loss of dignity or harm because of the online recruitment process to address the online privacy and confidentiality concerns. The moderators will ask the participants to give a short self-introduction, using pseudonyms/numbers, instead of their actual names, and not using video options. This includes not disclosing any of this information without the participants consent.

Selection of healthcare providers

Approximately 1 to 2 healthcare providers per hospital will be purposively selected and only those who 1) deliver SRH services, 2) are most knowledgeable about readiness and availability of SRH services 3) are stationed in the SRH clinic and have been working at the clinic for at least 6 months before the pandemic started will be selected for inclusion in the study. This information will be obtained from the facility in-charges.

Quantitative sample

Selection of Women

In each hospital, a maximum of 3 clients from each section (post-abortion care, family planning, SGBV, STI, abortion) will be consecutively selected to achieve up to 20 clients per hospital.

Selection of healthcare providers

One healthcare provider, preferably most knowledgeable in the health facility about SRH services provided, as determined by seniority, position or function, for example, will be selected to assist in the health facility assessment.

Data Collection

FGDs and IDIs will be used to understand client's perspectives on their experiences in accessing SRH services during and after peak transmission and healthcare providers' perspectives of SRH service availability and readiness in COVID-19-affected areas. The health system assessment will be used to assess the health system response to COVID-19. Study teams should be aware of the possibility that healthcare provider burnout or heavy workload may affect willingness or ability to participate in the study. Adaptations to data collection should be made to avoid overburdening the healthcare providers and clearly explained during recruitment.

Data Collection tools

There will be five sets of tools for clients- 1 FGD guide for women (published as **supplementary file S1**), 1 FGD guide for partners (published as **supplementary file S2**), 1 interview guide for women (published as **supplementary file S3**), 1 interview guide for men (published as

For peer review only - http://bmjopen.bmj.com/site/about/guidelines.xhtml

supplementary file S4) and a health services continuation questionnaire for women (published as part of **supplementary file S3**); and 1 interview guide for healthcare providers (published as **supplementary file S5**).

For the facility assessment, a facility and readiness assessment questionnaire has been developed based on the following validated tools [13-16]:

(1) WHO Service Availability and Readiness Assessment (SARA) guide
(2) WHO Hospital Readiness Checklist
(3) WHO safe abortion assessment tool
(4) SGBV Secual and gender-based violence Quality Assurance Tool

The facility and readiness assessment tool comprises five modules: (1) health services continuation (; (2) family planning services; (3) abortion services; (4) STI and (5) SGBV; in line with the study SRH focus areas (published as **supplementary file S3**). The following indicators will be used the assess the availability and readiness:

(1) Policies and plans
(2) Service maintenance and referrals
(3) Infrastructure
(4) Commodities
(5) Human resources

Each study site will adapt (including translation to local language) the tool to its specific context, taking into consideration the existing policies, SRH treatment guidelines, staffing norms and standards, types of facilities, national essential medicines lists and national health information systems among others. After adaptation to specific country context all tools should be piloted before being used.

Data Collection Methods

Participant Informed Consent Process

All study participants will be taken through a detailed informed consent process (for participation and audio-recording), which will be documented and those who agree to participate in the study will be asked for signed written (oral consent for those who cannot write, or for situations that necessitate online data collection) consent.

Given that data collection will be conducted during COVID-19, an assessment of the risks to the study team and participants should be made. The decision of whether to conduct face-to-face or remote data collection should be based on the risk assessment, followed by context-specific recommendations on adaptations to the original data collection process. Remote data collection will necessitate adaptation to the length of interviews and discussions, rapport building, oral informed consent, privacy and confidentiality measures as well as making special arrangements where access to internet and mobile connectivity is limited [17]. Face-to-face data collection will require special arrangements to ensure the safety of the study team and participants, following WHO as well as country-specific safety protocols, including educating and training the research

For peer review only - http://bmjopen.bmj.com/site/about/guidelines.xhtml

team and participants about COVID-19, provision of PPE and hygiene supplies, mandatory hygiene practices and sanitization of venues and equipment and physical distancing [18].

The FGDs and IDIs will be conducted at times and in venues that are considered both convenient and safe for the participants to freely discuss the subject matters. In addition, qualified researchers with experience in conducting IDIs and facilitating FGDs will be trained to ensure the validity of the data collection and will be selected based on the gender of the participants. The FGDs and IDIs will be semi-structured and follow a topic guide specific to each group of participants but will take place as a conversation in which the researchers promote a safe, comfortable environment to enable a comprehensive and candid record. In addition to the semi-structured interview, narrative interview techniques may be used depending on the participants' narratives about life events and reproductive healthcare needs.

The FGDs and IDIs will be audio-recorded. Prior consent for recording will be sought again from each participant. Anonymity of the participants will be ensured by removing any personal or family identifiers and all recorded sessions will be coded for purposes of identification with a date, geographical site and session number. After each session, the recording will be sent to the team supervisor for secure storage for transcription. The audio files will be encrypted and sent to an electronic database which will be shared with WHO. Therefore, only the research team will be authorized to listen to the recordings. These audio recordings will be retained until they have been transcribed and checked for accuracy, after which they will be destroyed.

The FGDs will explore knowledge about COVID-19, care-seeking during COVID-19 and risk perceptions and availability of SRH services (end-line FGD will also explore post-pandemic services in comparison to the pandemic period). Before starting the FGD, the facilitators will collect socio-demographic data for each participant, build rapport with and among participants and set ground rules to ensure positive group dynamics that foster an effective and rich discussion. The facilitators will also use this time to know and understand the emic categories (field research and viewpoints obtained from within the social group, from the perspective of the subject) used by locals to describe their perceptions and practices related to the study topics. Each FGD is expected to last approximately 60 – 90 minutes.

IDIs with women will explore the psychosocial effects of COVID-19 on fertility desires, knowledge of COVID-19, risk perceptions and concerns about effects on SRH, care seeking behaviors, experience and barriers in accessing SRH services reproductive health, particularly comprehensive abortion, STI prevention and treatment and SGBV care and support, and their related needs for accurate information and reproductive health services. IDIs with partners will explore their knowledge of COVID-19, risk perceptions and concerns about effects on SRH, their influence on the access to SRH services of their partners and find out what role they play when their partners require SRH services like contraception, comprehensive abortion care, STI prevention and treatment and SGBV care and support. Each interview is expected to last approximately 40 – 60 minutes. IDIs for the women and their partners will be conducted separately.

For peer review only - http://bmjopen.bmj.com/site/about/guidelines.xhtml

In some countries, abortion is illegal, posing challenges in data quality since participants, fearing reprisals, may not provide accurate responses to abortion-related questions. Necessary precautions should be taken to ensure the interviews are conducted in a supportive and non-judgmental manner, encouraging the participants to respond freely. These include selection and training of interviewers to enable them to overcome their biases and stereotypes about abortion, building trust and rapport with participants and selection of interview venue to guarantee privacy [19].

Given the sensitive nature of the data being collected, care must be taken in the selection of data collectors to ensure protection of participants. The gender, experience and attitudes of the data collectors are important considerations. Training of data collectors is essential with emphasis on the risks to the participants and how to protect them, the importance of non-judgmental attitudes and provision of necessary support to participants.

Interviews with healthcare providers will be based on WHO's six building blocks framework [20 with a focus on the delivery of the focus SRH services during COVID-19; their perceptions on the roles and responsibilities of different parties to provide these services in the COVID-19 context; health system capacity to provide good quality of care for people during COVID-19; training needs, attitudes, biases about contraception, abortion, STI and SGBV in the context of COVID-19; and perceived psychosocial effects on men and women, their families and local communities.

The health availability and readiness assessment will be implemented in all the selected facilities using a cross-sectional survey design to highlight gaps or service delivery issues during COVID-19 and recovery in service availability and readiness in the same health facilities. The assessment will also include a review of health facility records in family planning and contraception, STI care and treatment and care and support for women who have experienced SGBV to assess the availability, type and range of commodities offered. A follow-up assessment will be done at end-line. The follow-up survey plans to assess the recovery in service availability and readiness in the same health facilities. The research team will fill out the data extraction tool and the questionnaire with the assistance of the most knowledgeable person in the health facility about SRH services provided (senior health care provider and/or administrator) for each section of the questionnaire. The assessment will also include a questionnaire to the clients of the health facility.

The client questionnaire contains questions related to general characteristics of clients and their experience in seeking SRH services. All the selected hospitals will be required to collect this data from all clients seeking SRH services once every month throughout the study period, to capture trends in service availability and utilization.

Data Analysis

Health facility assessment data will use the presence of the tracer items for the provision of these services, such as availability of guidelines, staff and essential commodities. Data will be entered into an electronic database using data entry programmes such as Epi data and CSPro. Study sites

For peer review only

using electronic data collection may skip this step and for WHO supported centres implementing the study an online platform (OpenClinica) will be used. Descriptive analysis will be used to illustrate the basic characteristics of the different facilities, including the monthly number of clients, types of procedures provided, number of medical staff, stocks of drugs, etc.

Qualitative analysis will form the main analysis for this study, beginning while data collection is ongoing to assess progress and determine any problems. The audio-recorded data will be transcribed verbatim and de-identified by using ID numbers in place of names. Where required, transcriptions will be translated to English and back-translated and analyzed using content analysis, according to the suggested steps by Elo & Kyngäs (2008) [21], as illustrated in Table 1. The WHO team based in Geneva, in conjunction with the study principal investigator (PI) and project team, will support and monitor the data analysis.

Table 1. Planned analytical procedures for content analysis

Phase	Procedures
Preparation	Reading through verbatim transcriptions of the interviews several times to familiarize with the data, gain an understanding of what has been expressed, selecting the unit of analysis, deciding on the analysis of manifest content.
Organizing	Open coding and creating categories, grouping codes under higher order headings, formulating a general description of the research topic through generating categories and subcategories as abstracting.
Reporting	Reporting the analysis process and the results through models, conceptual systems, conceptual map or categories, and a story line.

Data management and access

Data management plans should include information about how data will be stored, including levels of protection, who will have access to the data and when it would be destroyed, how data will be transferred (if needed) securely. In countries where data protection legislation exists, protocols should specify that data would be handled in accordance with those policies.

Quantitative data

Data collectors will be trained on data collection, transmission, verification, storage and primary analysis to assess errors. Data can be collected electronically, or on paper.

Qualitative data

The quality and trustworthiness of the qualitative data collected will be assured through triangulation, as we will conduct interviews with different parties, including health professionals, health workers, and clients. The process of the study will be clearly documented to ensure the transparency and the rigor of the study.

For peer review only - http://bmjopen.bmj.com/site/about/guidelines.xhtml

Ethics and dissemination

Scientific approval has been obtained from WHO research project review panel (RP2). Ethical approval for this study was also obtained from the WHO Ethics Review Committee (protocol ID CERC.0103). Each study site is required to obtain the necessary ethical and regulatory approvals that are required in the corresponding country. Ethical considerations of informed consent, voluntary participation, privacy and confidentiality, anonymity and compensation for incidental costs (the decision to offer compensation and its value will depend on the specific country context and respective local policies) will be respected and detailed in the informed consent process.

Given the sensitive nature of the data being collected, special care will be taken to ensure the "do no harm" principle is respected. This will include making arrangements with locally available support services (medical, psychosocial, legal) where participants can be referred, training the research team to respond to and provide immediate emotional support to study participants or for situations where support services are not locally available or are inadequate [22, 23].

At country level the results will be presented to policy makers, researchers, managers through policy briefs and workshops in collaboration with the WHO office. The results will also be disseminated among the communities/participants through online platforms/text messages, which will be provided to the participants by the data collectors/health workers. In addition, the results will also be presented to the scientific and funding community in collaboration with WHO country and regional offices, by communication and manuscript publications in peer-reviewed international journals

Patient and public involvement

There was no patient or public involvement in the design of this protocol.

Discussion

This is a generic standardized protocol designed to be used across many different settings globally and to be useful as templates for use in health emergencies in the future. This will facilitate the comparison of results across regions and countries.

This study provides a unique opportunity to assess the availability, utilization and readiness of the SRH services during the COVID-19 pandemic. The main lesson learned will be the adaptations of the health system in pandemic situations and what can be done to ensure continuity of essential SRH services.

The mixed methods approach and panel design with two data collection points enable a comprehensive analysis of the barriers to availability, utilization and readiness of sexual and reproductive health services during COVID-19, as well as the post-pandemic recovery as transmission is contained.

This study is well placed to advocate for the development and strengthening of policies and services that are responsive to the needs of women and girls during health emergencies, given

For peer review only - http://bmjopen.bmj.com/site/about/guidelines.xhtml

For peer review

the potential to exacerbate existing gender and social inequalities and increase the vulnerability of women and girls to preventable health risks. The use of a mainly qualitative approach places women's rights and needs at the center of the debate. The inclusion of partners will enhance understanding of gender dynamics and support efforts towards identifying strategies to enhance positive male involvement and engagement in women's sexual reproductive health and rights.

Contributorship statement

Contributors: MA and CRK conceptualized and designed the protocol. All authors contributed to developing the detailed methodology. SK, EC and MA drafted the first draft manuscript. JS, CK, LB, VB, JGC, HK and PL edited the draft, provided critical inputs, and approved the final written version for publication.

Funding Statement

This research has been supported by German Federal Ministry of Health (BMG) COVID-19 Research and development funding to WHO. It has also been supported by the UNDP-UNICEF-WHO-World Bank Special Programme of Research, Development and Research Training in Human Reproduction (HRP); a co-sponsored programme executed by the World Health Organisation (WHO). The adaptation and implementation of this protocol in the HRP collaborating centres in Brazil, Burkina Faso, China, Ghana, Italy, Kenya, Pakistan, Thailand and the United Kingdom was also funded by the same. The views of the funding body have not influenced the content of this manuscript. This article represents the views of the named authors only and does not represent the view of their affiliated institutions.

Disclaimer

This report contains the collective views of an international group of experts, and does not necessarily represent the decisions or the stated policy of the World Health Organization.

Competing interests

The authors declare that they have no competing interests.

For peer review only

Acronyms

COVID-19	Corona Virus Disease 2019
CSPro	Census and Survey Processing System
FGD	Focus Group discussion
SGBV	Gender Based Violence
IDI	In-depth Interview
RP2	Project Review Panel
SRH	Sexual and Reproductive Health
STI	Sexually Transmitted Infections
WHO	World Health Organisation

References

1. Riley T, Sully E, Ahmed Z, Biddlecom A. Estimates of the potential impact of the COVID-19 pandemic on sexual and reproductive health in low- and middle-income countries. *Int Perspect Sex Reprod Health* 2020; 46:73–76. doi: 10.1363/46e9020.
2. Addressing human rights as key to the COVID-19 response. Geneva: World Health Organization; April 2020 (WHO/2019-nCoV/SRH/Rights/2020.1; https://www.who.int/publications/i/item/addressing-human-rights-as-key-to-the-covid-19-response (accessed August 15, 2020)
3. Sexual and Reproductive Health and Rights during the COVID-19 pandemic: A joint report by EPF & IPPF EN. April 2020. https://www.ippfen.org/sites/ippfen/files/2020-04/Sexual and Reproductive Health during the COVID-19 pandemic.pdf (accessed August 16, 2020)
4. COVID-19: operational guidance for maintaining essential health services during an outbreak: interim guidance. 25 March 2020. Geneva: World Health Organization. 2020 WHO/2019-nCoV/essential_health_services/2020.1; https://apps.who.int/iris/handle/10665/331561 (accessed on August 15, 2020).
5. Maintaining essential health services: operational guidance for the COVID-19 context. World Health Organisation; 2020. https://apps.who.int/iris/handle/10665/332240 (Accessed August 15, 2020)
6. Impact of the COVID-19 pandemic on family planning and ending secual and gender-based violence, female genital mutilation and child marriage: pandemic threatens achievement of the transformative results committed to by UNFPA. New York, United Nations Population Fund; 2020 https://www.unfpa.org/resources/impact-covid-19-pandemic-family-planning-and-endinggender-based-violence-female-genital-mutilation (accessed on August 15, 2020).
7. Millions more cases of violence, child marriage, female genital mutilation, unintended pregnancy expected due to the COVID-19 pandemic [Internet]. Unfpa.org. 2020 [cited 3 May 2020]. Available from: https://www.unfpa.org/news/millions-more-cases-violence-child-marriage-female-genital-mutilation-unintended-pregnancies#
8. Coronavirus Disease (COVID-19) Pandemic UNFPA Response Plan. June 2020 https://www.unfpa.org/sites/default/files/resource-pdf/UNFPA_Global_Response_Plan_Revised_June_2020_.pdf (accessed August 16, 2020)
9. United Nations Population Fund. Impact of COVID-19 on family planning: What we know one year into the pandemic. Technical note. New York, UNFPA;2021
10. Julia Hussein. COVID-19: What implications for sexual and reproductive health and right globally? *Sex Reprod Health Matters* 2020;28(1):1746065 DOI:10.1080/26410397.20201756065.
11. World Health Organization. Considerations for implementing and adjusting public health and social measures in the context of COVID-19. Interim guidance. Geneva, World Health Organization;2020 https://www.who.int/publications/i/item/considerations-in-adjusting-public-health-and-social-measures-in-the-context-of-covid-19-interim-guidance (Accessed December 5, 2020)
12. Gelinas L, Pierce R, Winkler S, et.al. Using social media as a research recruitment tool: ethical issues and recommendations. *Am J Bioeth 2017*;17(3):3-14

For peer review only - http://bmjopen.bmj.com/site/about/guidelines.xhtml

13. World Health Organization. Service availability and readiness assessment (SARA): implementation guide. Geneva: World Health Organization; 2015 http://www.who.int/healthinfo/systems/sara_implementation_guide/en/. (Accessed August 20, 2020)
14. World Health Organization. Safe abortion: technical and policy guidance for health systems. 2nd ed. Geneva: World Health Organization; 2012 http://www.who.int/reproductivehealth/publications/unsafe_abortion/9789241548434/en/. (Accessed March 10, 2021)
15. JHPIEGO. 2018. Secual and gender-based violence Quality Assurance Tool: Standards for the Provision of High-Quality Post-Violence Care in Health Facilities. https://www.who.int/reproductivehealth/publications/post-violence-care-in-health-facilities/en/ (Accessed March 10, 2021)
16. World Health Organisation. Rapid Hospital Readiness Checklist: Harmonized Health Service Capacity Assessment in the Context of the COVID-19 Pandemic. Interim Guidance. Geneva: World Health Organization; 2020 https://apps.who.int/iris/handle/10665/332779
17. Hensen B, Mackworth-Young CRS, Simwinga M, et al. Remote data collection for public health research in the covid-19 era: ethical implications, challenges and opportunities. *Health Policy Plan* 2021;czaa158 https://doi.org/10.1093/heapol/czaa158
18. IMPACT. 2020. SOPs for Data Collection during COVID-19. https://www.reachresourcecentre.info/wp-content/uploads/2020/05/DataCollectionSOPCOVID-19.pdf (Accessed March 10, 2021)
19. Zulu JM, Ali J, Hallex K, et.al. Ethical challenges in research on post-abortion care with adolescents: experiences of researchers in Zambia. *Glob Bioeth* 2018; 31(1):104-119
20. World Health Organisation. Everybody business: strengthening health systems to improve health outcomes: WHO's framework for action. Geneva, World Health Organisation; 2007. https://www.who.int/healthsystems/strategy/everybodys_business.pdf?ua=1 (Accessed August 15, 2020)
21. Elo S, Kyngäs H. The qualitative content analysis process. *J Adv Nurse* 2008;62:107-115.
22. UN Women, World Health Organisation. Violence Against Women and Girls. Data Collection during COVID-19. New York, UN Women; 2020 https://www.unwomen.org/-/media/headquarters/attachments/sections/library/publications/2020/vawg-data-collection-during-covid-19 (Accessed March 10, 2021)
23. World Health Organisation. Putting Women First: Ethical and Safety Recommendations for research on Domestic Violence Against Women. Geneva, World Health Organization;2001 https://apps.who.int/iris/bitstream/handle/10665/65893/WHO_FCH_GWH_01.1.pdf?sequence=1 (Accessed Jan 6, 2021)

For peer review only - http://bmjopen.bmj.com/site/about/guidelines.xhtml

Focused group topic guide for women

Demographic information (Note: Collect data on individuals before the FGD)

- Age (in completed years)
- Education (highest level attained)
- Residence (actual, urban, rural, slum)
- COVID-19 infection history (self, close family, work mates, friends)
- Social economic status - Employment status (i.e. formal or informal employment), income earning status and possibly income ranges as suitable in the country setting.

Knowledge about COVID-19 (such as cause, signs and symptoms)

1. What is COVID-19? What do you know about COVID-19?
   PROBE: for causes, transmission, signs, symptoms, sources of information about COVID-19
2. Did you perceive yourself to be at risk for COVID-19 infection?
   PROMPT: Probe for any special considerations (pregnancy, pre-existing health conditions, others).

Care seeking and perception of risk about being infected by COVID-19

3. The decision-making process of treatment seeking during COVID-19
   PROBE: Ask who influenced the decision to seek care during COVID-19 (self, partner, other family member, etc?)
4. Perception of risk of infection during SRH care seeking.
   PROMPT: How did this affect health seeking behaviour during COVID-19?
5. The treatment-seeking process during COVID-19
   a. Were your needs for accurate information on reproductive health services fulfilled? How? Or Why not? PROBE: information sources: brochures, social media, billboards, other printed media, information on service hours or location where services are offered etc.
   b. What were your needs for reproductive health services? Were these needs fulfilled? **PROBE**: Contraception? Abortion care? STI?
   c. Did you encounter any barriers in your bid to access needed reproductive health services? PROBES: transportation, behaviour of other persons, etc.

   PROMPT: Interviewer should probe for other context specific intermediaries to seeking care. E.g. paperwork for insurance
6. Do you think that reproductive services were readily available to clients who needed them? Specifically, the availability of the reproductive health medicine and medical supplies needed by clients based on the following:
   a. Availability of staff, medicines and supplies for SRH during COVID-19
   b. Schedule of treatments/appointments during COVID-19
      PROMPT: Probe for changes in the delivery of SRH services (specifically for family planning, abortion, post-abortion care and STI care) i.e. appointment-based system introduced.
   c. Interaction between the service provider and the patient was during COVID-19. PROBE: Interactions with different cadres of health workers. Nurses? Midwives? Doctors? Records? OPD staff? Etc.
   d. Was the interaction between service providers and staff described above different from pre-COVID-19 time? How?
7. What were some of the safety measures ensured by the health facility to protect clients from COVID-19 during services
8. Do you think that the COVID-19 had any impact on client protection and privacy during services?
   PROBE: What was done (or could have been) differently?

Post-pandemic recovery of the facilities to provide services in comparison to the pandemic period.

GENERAL COMMENTS: Based on the country situation, the probes for the questions above can be extended to cover the post-pandemic period if applicable.

For peer review only

Focused group topic guide for men

Demographic information

- Age (in completed years)
- Education (highest level attained)
- Residence (actual, urban, rural, slum)
- COVID-19 infection history (self, close family, work mates, friends)
- Social economic status: Employment status (i.e. formal or informal employment), income earning status and possibly income ranges as suitable in the country setting.

Knowledge about COVID-19 (such as cause, signs and symptoms)

1. What is COVID-19? What do you know about COVID-19?
   PROBE: for causes, transmission, signs, symptoms, sources of information about COVID-19
2. Did you perceive yourself to be at risk for COVID-19 infection?
   PROMPT: Probe for any special considerations (pregnancy, pre-existing health conditions, others).

Care seeking and perception of risk about being infected by COVID-19

1. What role did you play in the decision-making process of treatment seeking during COVID-19?
   PROBE: final decision maker (man only, joint, woman only, other); stage of involvement (decision stage or care seeking stage).
2. The role of men in the treatment-seeking process during COVID-19
   a. What role did you play in seeking for accurate information on reproductive health services? Were your needs fulfilled? How? Or Why not?
      PROBE: information sources: brochures, social media, billboards, other printed media, etc. The role of men in looking for sexual reproductive health services (including contraception and abortion care) and whether the needs were fulfilled
   b. What role did you play in looking for reproductive health services? Were your needs for reproductive health services fulfilled?
      PROBE: Contraception? Abortion care? STI?
   c. Did you encounter any barriers in your bid to access needed reproductive health services? PROBES: transportation, behaviour of other persons, entry restrictions for men, etc.
      NOTE: Interviewer should probe for other context specific intermediaries to seeking care. E.g. paperwork for insurance
3. Do you think that reproductive services were readily available to clients who needed them? The availability of the reproductive health medicine and medical supplies needed by clients?
   a. The schedule of treatments/appointments during COVID-19?
4. How was the interaction between the service provider and you as the male partner during COVID-19?
   PROBE: Interactions with different cadres of health workers. Nurses? Midwives? Doctors? Records? OPD staff? Etc
5. Was the interaction between service providers and staff described above different from pre-COVID-19 time? How?

For peer review only

6. What were some of the safety measures ensured by the health facility to protect clients from COVID-19 during services?
   PROBE: Were male partners stopped from entering consulting and treatment rooms due to COVID-19?
7. Do you think that the COVID-19 had any impact on client protection and privacy during services?
8. **PROBE**: What was done (or could have been) differently? E.g. barriers in seeking SRH services during COVID

Post-pandemic recovery of the facilities to provide services in comparison to the pandemic period.

COMMENT: Based on the country situation, the probes for the questions above can be extended to cover the post-pandemic period if applicable.

For peer review only

In-depth Interview topic guide for women

1. Demographic information
   - Age
   - Ethnicity
   - Marital status
   - Socio-economic status
   - Occupation
   - Education status
   - Religion
   - Place of residence and housing condition
   - Obstetric characteristics (no. of pregnancies, parity)

2. Knowledge about COVID-19
   - Cause (sexual transmission)
     - How do you think COVID-19 virus is transmitted?
     - Do you think it can be transmitted sexually?
   - Signs and symptoms of COVID-19
     - What are the sign and symptoms of COVID-19?
     - If yes, can you mention some that you know?
   - Care-seeking for COVID-19 infection
     - Do you know what to do/who to contact/ where to go if you experience COVID-19 signs and systems?
     - If yes, can you explain?
   - Sources of information and trusted communication channels (v. rumors, misinformation) Experience of COVID-19 (direct / indirect)
     - Where did you get information about COVID-19?
     - Was the information you obtained accurate?
     - Can you give me an example of accurate information you obtained?
     - Were there any rumors and/or misinformation about COVID-19?
     - How did you know that this information was just rumors or misinformation?
     - Have you fund sources of information that you can trust?
     - How did you know which information sources to trust?
     - Have you been infected by COVID-19?
     - Do you know of anyone who has been infected? If yes, was it a close friend or family member?
   - Perception of risk about being infected by COVID-19
     - Do you think you are at risk of being infected by the COVID-19 virus?
     - If no, can you tell me why not?
     - Do you think you can be infected whilst seeking care?
   - Ways to prevent being infected by COVID-19 (probe at individual, household, community levels)
     - Do you know how to prevent infection from COVID-19?
     - What can individuals do? What can households do? What can communities do?
   - Responsibility to prevent COVID-19 (probe at individual, household, community levels, authorities)
     - Who is responsible for the prevention of COVID-19 infection? Do you think this

For peer review only

responsibility was fulfilled by individuals, households, communities and authorities? How?

- Religious / socio-cultural practices and influencers
  - Are there any religious / socio-cultural practices that are used to prevent COVID-19 infection?
  - Does participation in any religious / socio-cultural practices make you practice prevention of COVID-19 differently from what is promoted by health professionals?

3. Sexual and Reproductive Health
   - Decision-making and agency to act
     - Who makes the decisions related to your sexual and reproductive health needs (self, husband/partner or family member)?
     - Who is usually responsible for carrying out the decision?
   - Issues of access / utilization
     - Did you need to seek SRH services during COVID-19?
     - If yes, were you able to access the services? How did you access the services?
     - If no, what were the barriers? What did you do?
   - Challenges faced
     - Are there any other challenges you faced in seeking SRH services during COVID-19? What are they and how did you address/manage the challenges?

4. Contraceptive practices
   - Changes in practice (if any) due to COVID-19
     - Which method of contraception do you normally use?
     - Did you have to change your normal method of contraception during COVID-19? If yes, which method and what influenced the change?
     - If no, why didn't you change from your normal practice?
   - Is pregnancy being avoided / delayed due to COVID-19? If yes, why?
   - Access to / utilization of different methods of contraception (barriers / drivers)
   - Which methods of contraception were accessible to you during COVID-19?
   - Was there a difference in the methods of contraception accessible to you compared to before COVID-19?
   - What was causing the difference?
   - Challenges in access / utilization
   - Are there any other challenges you faced in accessing/utilizing contraception services during COVID-19?
   - If yes, what were these challenges and how did you cope with them?

5. Induced abortion
   - Perceptions about abortion at normal times
   - The role abortion plays in women's lives
     - Why do you think women seek abortion services?
     - Do you think abortion is an essential service for women? If yes, in what way?
   - Fear of stigma
     - How does family, community and society view women who seek or have sought abortion services?

For peer review only - http://bmjopen.bmj.com/site/about/guidelines.xhtml

    - Does this stigma affect your decision whether to have an abortion?
    - Does this stigma affect how you will seek abortion services? If yes, how?
    - What do you think are the effects of this stigma on women who need abortion services?
  - Inequities in access to safe abortion
    - Do you think safe abortion services are equally accessible to all women who need them?
  - Perceptions about abortion during COVID-19
    - Would you choose to seek the abortion services during COVID-19, if you need one?
    - If no, why not? If yes, where and how would you access the service?
- Access to different methods of abortion (barriers / drivers)
  - Did you seek abortion services during COVID-19? If yes, which abortion methods were available for you (medical abortion or surgical abortion)?
  - What pushed you to have an abortion? If you were given a choice of methods, which one did you choose and why?
  - Did you face any barriers in seeking and accessing abortion services during COVID-19?
  - Challenges in accessing / utilizing safe abortion services (inequities in access)
    - Are there any other challenges you faced in accessing/utilizing safe abortion services? If yes, what were the challenges and how did you address them?

  - 9. Violence against women (*Interviewer should use the term that is used locally to describe VAW)*

  - Formal and informal systems of support, and coping strategies for VAW
    - Have you or any woman you know experienced any form of VAW?
    - What are some of the systems of support available for women who have experienced VAW?
    - Do you know where and how to access this support?
    - What are some of the coping strategies women who have experience such violence use?
  - Perceptions of safety in home and in community safety/increased risk during COVID-19
    - Do you think are safer or at increased risk of experiencing VAW at home or in the community during COVID-19?
    - What has made it safer/riskier for women during COVID-19?
  - Women's expectations of services for gender-based violence
    - If you or any other woman experiences GBV during COVID-19, do you expect the care and support services to be available?
    - Do you know where and how to access these services?
    - Do you expect any barriers to accessing these services during COVID-19? If yes, what kind of barriers?
  - What are additional stress factors (e.g. school-age children at home, older parents, loss of job/income, partner loss of job, etc.) that increase the likelihood of VAW?
  - How does stress affect frequency and severity of violence, particularly during COVID-19?

For peer review only

6. Sexually transmitted infections *(interviewer should use the locally known terms for STIs and the specific diseases)*
    - Perceptions about STI
        - Is anybody at risk of getting a STI? Why or why not?
        - How are people who are known to have a STI perceived in the community?
    - Experience of STI during COVID-19
        - Have you required care and treatment for a STI during COVID-19? If yes, where and how did you seek this service?
        - Were there any barriers to accessing this service? If yes, which ones and what did you do?
        - If no, would you seek care and treatment if required during COVID-19?
        - If no, why not? If yes, where and how would you access this service?

7. Psycho-social
    - Psychosocial issues associated with being infected with COVID-19 (probe: more significant for women/men, more significant for pregnant women)
        - Did you experience any stress, anxiety or depression during COVID-19? If yes, at what level (mild, moderate, severe)?
        - What do you think led to the psychosocial issues you experienced? Was it due to being infected or other related issues or both?
        - Did your partner also experience any psychosocial issues during COVID-19?
        - Who was most affected, yourself or your partner?

8. Final questions
    - On-going concerns about COVID-19 and its possible impact
        - Are there any other concerns about COVID-19 and its impact on you, your family or in general that you wish to express?
    - Needs related to accurate information (probe further information needs – NB consider how to provide information if necessary)
        - Was accurate information readily available and accessible during COVID-19?
        - Was it easy to understand?
        - Was the information that was available adequate?
        - Was there additional information you would have liked to obtain but was missing?
        - Are there better ways of making accurate information accessible to you and others?
    - Needs related to sexual reproductive health services (probe further SRH needs – NB consider how to support/provide if necessary)
    - Do you think SRH services were responsive to your needs during COVID-19?
    - If no, which of your needs were not met?
    - Which SRH services are most important for you, that should be available even in health emergencies like COVID-19?
    - How do you think provision of SRH services can be supported during other health emergencies like COVID-19?

For peer review only

Additional themes will be added as appropriate for each target group and questions revised accordingly (with attention to first person / third person views), and across the three phases of the study.

For peer review only

In-depth Interview topic guide for men

1. Demographic information
    - Age
    - Ethnicity
    - Marital status
    - Socio-economic status
    - Occupation
    - Education status
    - Religion
    - Place of residence and housing condition

2. Knowledge about COVID-19
    - Cause (sexual transmission)
        - How do you think COVID-19 virus is transmitted?
        - Do you think it can be transmitted sexually?
    - Signs and symptoms of COVID-19
        - What are the sign and symptoms of COVID-19?
        - If yes, can you mention some that you know?
    - Care-seeking for COVID-19 infection
        - Do you know what to do/who to contact/ where to go if you experience COVID-19 signs and systems?
        - If yes, can you explain?
    - Sources of information and trusted communication channels (v. rumors, misinformation) Experience of COVID-19 (direct / indirect)
        - Where did you get information about COVID-19?
        - Was the information you obtained accurate?
        - Can you give me an example of accurate information you obtained?
        - Were there any rumors and/or misinformation about COVID-19?
        - How did you know that this information was just rumors or misinformation?
        - Have you fund sources of information that you can trust?
        - How did you know which information sources to trust?
        - Have you been infected by COVID-19?
        - Do you know of anyone who has been infected? If yes, was it a close friend or family member?
    - Perception of risk about being infected by COVID-19
        - Do you think you are at risk of being infected by the COVID-19 virus?
        - If no, can you tell me why not?
        - Do you think you can be infected whilst seeking care?
    - Ways to prevent being infected by COVID-19 (probe at individual, household, community levels)
        - Do you know how to prevent infection from COVID-19?
        - What can individuals do? What can households do? What can communities do?
    - Responsibility to prevent COVID-19 (probe at individual, household, community levels, authorities)
        - Who is responsible for the prevention of COVID-19 infection? Do you think this responsibility was fulfilled by individuals, households, communities and

authorities? How?

- Religious / socio-cultural practices and influencers
  - Are there any religious / socio-cultural practices that are used to prevent COVID-19 infection?
  - Does participation in any religious / socio-cultural practices make you practice prevention of COVID-19 differently from what is promoted by health professionals?

3. Sexual and Reproductive Health
   - Decision-making and agency to act
     - Who makes the decisions related to the sexual and reproductive health needs of your partner (you, partner or family member)?
     - Who is usually responsible for carrying out the decision?
   - Issues of access / utilization
     - Did your partner need to seek SRH services during COVID-19?
     - If yes, was your partner able to access the services? How did your partner access the services?
     - If no, what were the barriers? What did you and/or your partner do?
     - What role did you play in ensuring your partner sought and/or accessed the needed SRH services?
   - Challenges faced
     - Are there any other challenges your partner faced in seeking SRH services during COVID-19? What are they and how did you and/or your partner address/manage the challenges?

4. Contraceptive practices
   - Changes in practice (if any) due to COVID-19
     - Which method of contraception do you and/or your partner normally use?
     - Did you have to change your normal method of contraception during COVID-19? If yes, which method and what influenced the change?
     - If no, why didn't you change from your normal practice?
   - Did your partner avoid / delay pregnancy due to COVID-19? If yes, why?
   - Access to / utilization of different methods of contraception (barriers / drivers)
   - Which methods of contraception were accessible to you and your partner during COVID-19?
   - Was there a difference in the methods of contraception accessible to you compared to before COVID-19?
   - What was causing the difference?
   - Challenges in access / utilization
   - Are there any other challenges you and your partner faced in accessing/utilizing contraception services during COVID-19?
   - If yes, what were these challenges and how did you cope with them?
   - What role did you play in ensuring your partner sought and/or accessed contraception services?

5. Induced abortion

For peer review only

- Perceptions about abortion at normal times
- The role abortion plays in women's lives
  - Why do you think women seek abortion services?
  - Do you think abortion is an essential service for women? If yes, in what way?
- Fear of stigma
  - How does family, community and society view women who seek or have sought abortion services?
  - Does this stigma affect your decision whether to have an abortion?
  - Does this stigma affect how you will seek abortion services? If yes, how?
  - What do you think are the effects of this stigma on women who need abortion services?
- Inequities in access to safe abortion
  - Do you think safe abortion services are equally accessible to all women who need them?

- Perceptions about abortion during COVID-19
  - Would you choose to seek the abortion services during COVID-19, if your partner needed one?
  - If no, why not? If yes, where and how would you access the service?
- Access to different methods of abortion (barriers / drivers)
  - Did your partner seek abortion services during COVID-19? If yes, which abortion methods were available (medical abortion or surgical abortion)?
  - What pushed you and your partner to have an abortion? If you were given a choice of methods, which one did you choose and why?
  - Were there any barriers in seeking and accessing abortion services during COVID-19?
- Challenges in accessing / utilizing safe abortion services (inequities in access)
  - Are there any other challenges you faced in accessing/utilizing safe abortion services? If yes, what were the challenges and how did you address them?
- What was your role in ensuring your partner accessed the abortion service?

6. Violence against women (*Interviewer should use the term that is used locally to describe VAW)*
   - Formal and informal systems of support, and coping strategies for VAW
     - Has your partner or any woman you know experienced any form of VAW?
     - What are some of the systems of support available for women who have experienced VAW?
     - Do you know where and how to access this support?
     - What are some of the coping strategies that women who have experienced such violence use?
   - Perceptions of safety in home and in community safety/increased risk during COVID-19
     - Do you think women are safer or at increased risk of experiencing VAW at home or in the community during COVID-19?
     - What has made it safer/riskier for women during COVID-19?
   - Women's expectations of services for gender-based violence

For peer review only

- If your partner experiences GBV during COVID-19, do you expect the care and support services to be available?
- Do you know where and how to access these services?
- Do you expect any barriers to accessing these services during COVID-19? If yes, what kind of barriers?
- What do you perceive as your role in ensuring your partner get the care and support needed?

- What are additional stress factors (e.g. school-age children at home, older parents, loss of job/income, partner loss of job, etc.) that increase the likelihood of VAW?
- How does stress affect frequency and severity of violence, particularly during COVID-19?

6. Sexually transmitted infections *(interviewer should use the locally known terms for STIs and the specific diseases)*

- Perceptions about STI
  - Is anybody at risk of getting a STI? Why or why not?
  - How are people who are known to have a STI perceived in the community?
- Experience of STI during COVID-19
  - Has your partner required care and treatment for a STI during COVID-19? If yes, where and how did you seek this service?
  - Were there any barriers to accessing this service? If yes, which ones and what did you do?
  - If no, would you seek care and treatment if required during COVID-19?
  - If no, why not? If yes, where and how would you access this service?
  - What was your role in ensuring your partner accessed the care and treatment?

7. Psycho-social

- Psychosocial issues associated with being infected with COVID-19 (probe: more significant for women/men, more significant for pregnant women)
  - Did you experience any stress, anxiety or depression during COVID-19? If yes, at what level (mild, moderate, severe)?
  - What do you think led to the psychosocial issues you experienced? Was it due to being infected or other related issues or both?
  - Did your partner also experience any psychosocial issues during COVID-19?
  - Who was most affected, yourself or your partner?

8. Final questions

- On-going concerns about COVID-19 and its possible impact
  - Are there any other concerns about COVID-19 and its impact on you, your family or in general that you wish to express?
- Needs related to accurate information (probe further information needs – NB consider how to provide information if necessary)
  - Was accurate information readily available and accessible during COVID-19?
  - Was it easy to understand?
  - Was the information that was available adequate?
  - Was there additional information you would have liked to obtain but was

For peer review only

missing?
- Are there better ways of making accurate information accessible to you and others?

- Needs related to sexual reproductive health services (probe further SRH needs – NB consider how to support/provide if necessary)
- Do you think SRH services were responsive to your needs during COVID-19?
- If no, which of your needs were not met?
- Which SRH services are most important for you, that should be available even in health emergencies like COVID-19?
- How do you think provision of SRH services can be supported during other health emergencies like COVID-19?

Additional themes will be added as appropriate for each target group and questions revised accordingly (with attention to first person / third person views), and across the three phases of the study.

For peer review only

In-depth Interview topic guide for SRH healthcare providers

Demographic information

- Age
- Ethnicity
- Marital status
- Socio-economic status
- Occupation
- Education status
- Religion

Health facility service availability and readiness

Health service delivery

1. Mobilization of resources for the diagnosis and the treatment of COVID-19 patients by the *(insert Country name*) government happened during COVID-19. Did the mobilization involve your facility? How was the facility involved? (Probe community, regional or national level involvement)

   COMMENT: *Indicate if facility was a designated COVID-19 treatment Centre*
2. What was the emergency response plan of your facility during the outbreak?
3. How did the mobilization of resources towards COVID-19 patients impact the service delivery in your facility, including the impact on human resources, space, medical supplies, etc.?
4. Did the mobilization of resources negatively or positively impact service delivery in your facility? Elaborate
5. Was there a set of essential Sexual & Reproductive Health services outlined to be maintained during the lockdown phase of the COVID-19 pandemic?
   PROMPT: Ask for each of the following services – family planning, abortion, post-abortion care, care and support for GBV and STI prevention and treatment
6. Were there disruptions to any of the SRH essential services outlined? What was the nature (services suspended, limited or shifted) of disruptions observed for each of these essential services (if any)?
   PROBES: for causes (lack of personnel, equipment, medicines or reduced demand, physical space), changes in usual service provision (introduction of appointment-based systems, telemedicine, shifting of usual location) and level of disruption (partial/ complete/not disrupted)
7. Do you think the delivery of SRH services during COVID-19 adequately responded to the needs of the clients at that time?
   PROBE: in what way?
8. What has the recovery process been like and what was the turning point?
   NOTE: Only for applicable countries.
   PROBE for coping mechanisms or approaches to ensuring SRH service delivery.

Health workforce （Human resources）

9. How did COVID-19 affect the health status of health workforce, such as doctors, nurses, ambulance drivers and hospital managers etc.? What measures were taken to protect them from the effects of the epidemic?

For peer review only

PROBE: for the provision of PPE, type of PPE, adequacy of supply and frequency of supply.

10. Was there any form of training on COVID-19? PROMPT: Probe for details of the training if provided – What areas of COVID-19? What form did this training take? Who received this training?
11. Were there employees infected? If someone was infected, how did the infection happen? What was the impact on service delivery due to the infection of SRH workers? How did it influence the service delivery?

COMMENT*: In facilities where SRH staff also provide general care in health facility, ask for the impact of an infected clinical staff in the facility even if not SRH-specific*

12. While knowing that they were at higher risk of contracting the virus, did female workers still have to fulfil the obligations such as caring for elders and immediate family (such as children or young dependents)? Is there any other conflict or challenge they faced during COVID-19? Note: Probe for any challenge worth considering in current setting.

Health information system

13. How does the reporting system for infectious diseases run?
    PROMPT: Probe for reporting system for COVID-19.
14. How did this system influence the availability of services in SRH facilities?
15. How did you keep information about the COVID-19 and SRH services flowing during the outbreak with your upper-level medical institutions and potential clients in your community?
    PROBE: how were changes in the delivery of SRH services communicated to clients? How was important COVID-19 information communicated to clients?

Access to essential medicines

16. Was the access to essential medicines, including regular medicines such as birth control pills, contraceptive methods impacted because of the lockdown/shutdown of the city during the COVID-19?

COMMENT*: Shutdown can be replaced with lockdown/restrictions depending on what the COVID-19 response was in country using this guide*

PROBE: ask about medicines for all the study focus SRH services

17. How has the outbreak affected the access to essential medicines in SRH for the patients and the general population?

PROBE: for each- contraceptive methods, abortion, post abortion care, STI prevention and treatment and GBV care and support

COMMENT: Probe separately for routine medication (such as antibiotics, analgesics etc where applicable)

Health systems financing

18. How did the COVID-19 epidemic affect the availability of funds at your facility?
    PROMPT: Inquire about effect on the financing and maintenance of essential SRH services and possible disruptions due to the pandemic, availability of additional government funding allocated to assuring essential health services.

For peer review only

> ***COMMENT:*** *This section should be informed by the existing health financing policy (i.e. National health insurance, existing SRH related exemptions and waiver systems) or COVID-19 specific directives on financing.*

Leadership and governance

19. What are your perceptions on the roles and responsibilities of providing SRH services during outbreaks like COVID-19?
    PROBE: where does the primary responsibility to ensure continuity of services lie? Who are the enablers, implementers?
20. How did your facility respond to the emergency and manage to ensure the SRH-related service delivery in your facility?
    PROMPT: Probe for country-specific plan, policies for maintaining SRH service provision during the pandemic.
    PROBE: separately for, family planning, STI and abortion services, services prioritized by the facility during the pandemic.

Health service provider perceptions on the delivery of specific SRH services in the context of COVID-19

A. Gender based Violence

Perceptions of changes in the nature and magnitude of violence against women, and especially domestic violence during COVID-19

- Have you noticed any change in the number of women coming into the health facility reporting gender-based violence since the COVID-19 crisis began?
    - Follow up: Increase or decrease?
    - Follow up: Have the women coming in who report experiencing violence mostly been returning patients, or mostly been new patients?
    - Follow up: Have you noticed an increase in the frequency of abuse that women are experiencing since movement restrictions were put in place?
    - If possible, specify types of violence (physical, sexual, psychological by a partner), non-partner sexual abuse, other

- Do you think that the violence women have been experiencing has become more severe during the crisis, for example has there been an increase in injuries? Violence during pregnancy?
    - Follow up: Have the types of violence that women are reporting changed? How so?

Nature of service provision during COVID-19, whether services for VAW are being changed or adapted and if safety considerations are adequately addressed?

- Since the start of COVID-19, has your health facility changed or adapted services for survivors ‘of violence?
    - Probe: (if not mentioned) In some health centers, providers have maintained ‘case management’ using video calls, or text messages. Did your health center make use of any new technologies to maintain services? If so, did you develop a protocol to ensure safety of survivors?

For peer review only

Access to /use of health services for VAW and related challenges

- Some health centers have had to deal with shortages of personal protective equipment and medications that have impacted their ability to offer services during the COVID-19 crisis. Have there been any new barriers to delivering services to survivors at your health facility?
    - What are these barriers and what approaches are being used to overcome these barriers?

- In a lot of places, external referral systems have also been impacted by the crisis, such as lower numbers of available spaces in shelters due to the need for physical distancing, or NGOs that have been temporarily shut down. To your knowledge, have any changes have been made to your external referral systems? What are these?
    - Follow up: Are any of the services to which you typically refer survivors available in a more limited capacity, or no longer available due to the crisis?

Attitudes towards provision of SRH services in the context of COVID-19

- Do you think it is essential to provide services for VAW during COVID-19? Why do you think so?

B. <u>Induced abortion</u>

Perceptions of changes in the demand for abortion during COVID-19.

- Have you noticed any change in the number of women seeking abortion services at your facility during COVID-19?
    - Follow up: Increase or decrease?
    - Follow-up: any changes in the abortion methods requested?
    - What do you think were the factors driving this change?

Delivery of safe abortion services during COVID-19?

- Where and how can people seek contraceptive services during COVID-19?
    - How many methods of abortion were available in your health facility during COVID-19? Which method of abortion did you suggest during COVID-19? Why?
    - What challenges did you meet in providing safe abortion services during COVID-19? What approaches did you use to overcome these challenges?

Attitudes towards provision of SRH services in the context of COVID-19

- Do you think it is essential to provide safe abortion services during COVID-19? Why do you think so?

C. <u>Contraceptive services</u>

Perceptions of changes in the demand for contraception services during COVID-19.

- Have you noticed any change in the number of women seeking contraception services at your facility during COVID-19?
    - Follow up: Increase or decrease?
    - Follow-up: any changes in the contraception methods requested?
    - What do you think were the factor driving this change?

Delivery of contraception services during COVID-19?

- Where and how can people seek contraceptive services during COVID-19?

For peer review only

- How many kinds of contraceptive methods were available in your health facility during COVID-19?
- What are the new/ altered ways by which contraceptive services are provided as a response to COVID-19?
- What challenges did you meet in providing contraceptive services during COVID-19? What approaches did you use to overcome these challenges?

Attitudes towards provision of SRH services in the context of COVID-19

- Do you think it is essential to provide contraception services during COVID-19? Why do you think so?

D. Prevention and treatment for sexually transmitted infections

Perceptions of changes in the demand for care and treatment for STI during COVID-19.

- Have you noticed any change in the number of women seeking care and treatment for STI at your facility during COVID-19?
  - Follow up: Increase or decrease?
  - What do you think were the factor driving this change?

Availability of STI care and treatment during COVID-19

- Where and how can people seek care and treatment for STI during COVID-19?
  - What are the new/ altered ways by which care and treatment for STI services are provided as a response to COVID-19?
  - Were all the medicines and supplies for providing the care and treatment available during COVID-19?
  - What challenges did you meet in providing care and treatment for STI services during COVID-19? What approaches did you use to overcome these challenges?

Attitudes towards provision of SRH services in the context of COVID-19

- Do you think it is essential to provide prevention and treatment for STIs during COVID-19? Why do you think so?

3. Perceived psychosocial effects of COVID-19
   - Did you experience stress/anxiety/depression during COVID-19?
   - How would rate your psychosocial health?
   - What role did COVID-19 play in influencing your psychosocial health?
   - In what way?
   - What do you think is the psychosocial impact of COVID-19 on people?
   - Do you think the impact was the same for men and women? If yes, why?
   - If no, in what ways is the psychosocial impact different?
   - What do you think is the psychosocial impact of COVID-19 on families?
   - What do you think is the psychosocial impact of COVID-19 on communities?

4. Final questions
   - On-going concerns about COVID-19 and its possible impact
     - Are there any other concerns about COVID-19 and its impact on you, your family or in general that you wish to express?
   - Needs related to accurate information (probe further information needs – NB consider how to provide information if necessary)

For peer review only

- Was accurate information readily available and accessible during COVID-19?
- Was it easy to understand?
- Was the information that was available adequate?
- Was there additional information you would have liked to obtain but was missing?
- Are there better ways of making accurate information accessible to you and others?

- Needs related to sexual reproductive health services (probe further SRH needs – NB consider how to support/provide if necessary)
- Do you think SRH services were responsive to the needs of patients during COVID-19?
- If no, which of needs were not met?
- Which SRH services are most important, that should be available even in health emergencies like COVID-19?
- How do you think provision of SRH services can be supported during other health emergencies like COVID-19? E.g. barriers in seeking SRH services during COVID

Additional themes will be added as appropriate for each target group and questions revised accordingly (with attention to first person / third person views), and across the three phases of the study.

For peer review only

BMJ Open

Health systems analysis and evaluation of the barriers to availability, utilization and readiness of sexual and reproductive health services in COVID-19 affected areas: A WHO mixed methods study protocol

Journal:	*BMJ Open*
Manuscript ID	bmjopen-2021-057810.R2
Article Type:	Protocol
Date Submitted by the Author:	25-Feb-2022
Complete List of Authors:	Kouanda, Seni; Institut de Recherche en Sciences de la Sante; Institut Africain de Sante Publique<br>Nahyuha Chomi, Eunice; Research Institute of Health Sciences<br>Kim, Caron; World Health Organization, Department of Sexual and Reproductive Health and Research. Preventing Unsafe Abortion Team<br>Jen, Sothornwit; Khon Kaen University, Department of Obstetrics and Gynaecology<br>Bahamondes, Luis; State University of Campinas – UNICAMP, Obst & Gynecol<br>Cecatti, Jose; University of Campinas, Obstetrics and Gynecology<br>Lumbiganon, Pisake; Khon Kaen University, Faculty of Medicine<br>Emefa, Modey; University of Ghana<br>Brizuela, Vanessa; WHO, Department of Reproductive Health and Research<br>Kuganantham, Hamsadvani; World Health Organization, Department of Reproductive Health and Research<br>Seuc, Armando; World Health Organization<br>Ali, Moazzam; Organisation mondiale de la Sante,
**Primary Subject Heading**:	Public health
Secondary Subject Heading:	Health policy, Health services research, Infectious diseases
Keywords:	COVID-19, PUBLIC HEALTH, Reproductive medicine < GYNAECOLOGY

SCHOLARONE™
Manuscripts

Manuscript Protocol

Health systems analysis and evaluation of the barriers to availability, utilization and readiness of sexual and reproductive health services in COVID-19 affected areas: A WHO mixed methods study protocol

- Kouanda, Seni-Reproductive Health Unit, Institute for Research in Health Sciences, Ouagadougou, Burkina Faso
- Chomi, Eunice- Reproductive Health Unit, Institute for Research in Health Sciences, Ouagadougou, Burkina Faso
- Kim, Caron- Department of Reproductive Health and Research, World Health Organization, Avenue Appia 20, 1211 Genève 27, Switzerland
- Sothornwit, Jen- Department of Obstetrics and Gynaecology, Faculty of Medicine, Khon Kaen University, Khon Kaen, Thailand.
- Bahamondes, Luis- School of Medical Sciences, University of Campinas (UNICAMP), Campinas, Brazil
- Cecatti, Jose Guilherme- School of Medical Sciences, University of Campinas (UNICAMP), Campinas, Brazil
- Lumbiganon, Pisake- Faculty of Medicine, Khon Kaen University, Khon Kaen, Thailand
- Modey, Emefa-Department of population, family and Reproductive Health, University of Ghana, Legon-Accra, Ghana.
- Brizuela, Vanessa- Department of Reproductive Health and Research, World Health Organization, Avenue Appia 20, 1211 Genève 27, Switzerland
- Kugananthan, Hamsa- Department of Reproductive Health and Research, World Health Organization, Avenue Appia 20, 1211 Genève 27, Switzerland
- Seuc, Armando Humberto, Consultant, Department of Reproductive Health and Research, World Health Organization, Avenue Appia 20, 1211 Genève 27, Switzerland
- Ali, Moazzam* on behalf of WHO HRP social Science Research Team**- Department of Reproductive Health and Research, World Health Organization, Avenue Appia 20, 1211 Genève 27, Switzerland

*Corresponding author: Moazzam Ali (alimoa@who.int). Telephone: +41-(0)22-791-3442
Department of Reproductive Health and Research, World Health Organization, Avenue Appia 20, 1211 Genève 27, Switzerland.

**** WHO HRP Social Science Research Team**: **Brazil** (Luis Bahamondes, Jose Guilherme Cecatti); **Burkina Faso** (Eunice Chomi, Seni Kouanda); **China** (Kun Tang, Hanxiyue Zhang, Yifan Zhu, Yueping Guo, Ge Yang and Chunxiao Peng); **Ghana** (Deda Ogum Alangea, Kwasi Tropsey, Emefa Judith Modey); **Pakistan** (Rozina Karmaliani, Laila Ladak); **Thailand** (Pisake Lumbiganon, Jen Sothornwit); **Kenya** (Marleen Temmerman, Abdu Mohiddin, Ferdinand Okwaro); **Italy** (Massimo Mirandola, Maddalena Cordioli, Alessia Savoldi, Simone Garzon, Stefano Uccella, Ranieri Poli.) **UK** (Nigel Sherriff, Alexandra Sawyer, Jorg Huber, Jaime Vera, Debbie Williams).

For peer review only

WHO Secretariat (Moazzam Ali, Caron Kim, Hamsadvani Kuganantham, Igor Toskin, Vanessa Brizuela, Anna Thorson, Joy Jerop Chebet, Hugo Gamerro Abrego , Soe Soe Thwin, Armando Seuc)

Word count (excluding title page, abstract, references, figures and tables)- 4345

KEYWORDS
Contraception, sexual and reproductive health, abortion services, COVID-19, facility assessment, sexual and gender-based violence, sexually transmitted infections

For peer review only

Abstract

Introduction The novel Corona Virus Disease 2019 (COVID-19) has led to an unprecedented increase in demand on health systems to care for people infected, necessitating the allocation of significant resources, especially medical resources, towards the response. This, compounded by the restrictions on movement instituted may have led to disruptions in the provision of essential services, including sexual and reproductive health (SRH) services. This study aims to assess the availability of contraception, comprehensive abortion care, sexually transmitted infection (STI) prevention and treatment and sexual and gender-based violence (SGBV) care and support services in local health facilities during COVID-19 pandemic. This is a standardized generic protocol designed for use across different global settings.

Methods and Analysis This study adopts both quantitative and qualitative methods to assess health facilities' SRH service availability and readiness, and clients' and providers' perceptions of the availability and readiness of these services in COVID-19-affected areas. The study has 2 levels: (1) Perceptions of clients (and their partners) and healthcare providers, using qualitative methods, and (2) assessment of infrastructure availability and readiness to provide SRH services through reviews, facility service statistics for clients and a qualitative survey for healthcare provider perspectives. The health system assessment will use a cross-sectional panel survey design with two data collection points to capture changes in SRH services availability as a result of the COVID-19 epidemic. Data will be collected using focus group discussions, in-depth interviews and a health facility assessment survey.

Ethics and Dissemination Ethical approval for this study was obtained from the World Health Organisation (WHO) Scientific and Ethics Review Committee (protocol ID CERC.0103). Each study site is required to obtain the necessary ethical and regulatory approvals that are required in each specific country.

Strengths and limitation of this study

- The study approach and design enable a comprehensive analysis of the barriers to availability, utilization and readiness of sexual and reproductive health services during COVID-19, as well as the post-pandemic recovery as transmission is contained.
- The use of a mainly qualitative approach places women's rights and needs during health emergencies at the center of the debate, underscoring the need for more responsive policies.
- The inclusion of partners will enhance understanding of gender dynamics and support efforts towards identifying strategies to enhance positive male involvement and engagement in women's sexual reproductive health and rights.
- A possible limitation is that the study will be conducted during and after one of the waves of the pandemic, at which stage there may have been changes in the health system as lessons are learnt.
- Another limitation is that the estimation of the recovery period may be difficult in settings where the pandemic is yet to be brought under control. This part of the analysis may be subject to delays until an appropriate period is determined.

For peer review only - http://bmjopen.bmj.com/site/about/guidelines.xhtml

INTRODUCTION

The novel COVID-19 disease, which was first reported in December 2019 and rapidly spread globally, has led to an unprecedented increase in demand on health systems to care for people infected, necessitating the allocation of significant resources, especially medical resources, towards the response. The increased demand, compounded by the restrictions on movement instituted as part of containment measures may have led to disruptions in the provision of essential services, including sexual and reproductive health (SRH) services [1,2].

The novel coronavirus is new to humans and only limited scientific evidence is available on the impact of COVID-19 on SRH service delivery. However, lessons from the Ebola and Zika virus outbreaks have highlighted the severe disruptions in SRH services that expose women and girls in particular to preventable health risks [2,3]. Some services may be unavailable due to either facilities and health workers being repurposed to care for COVID-19 patients, patient safety concerns, movement restrictions disrupting travel to health facilities, supply chain disruptions or a reduction in health workers because of increasing numbers being themselves infected by COVID-19 [4,5]. In addition, overwhelmed with COVID-19 cases, clinical staff may not have the time or personal protective equipment (PPE) needed to provide family planning counselling and commodities [5]. Recent evidence suggests disruptions lasting 3-6 months in 2020 left between 4-23 million women in low- and middle-income countries unable to access modern contraceptives, a projected 1.4 million (500,000-2.7 million) unintended pregnancies and an additional 31 million cases of secual and gender-based violence (SGBV) [1, 6-9]. Furthermore, studies have modelled the potential impact, showing that even a 10% reduction in essential SRH services could lead to an estimated 15 million unintended pregnancies, 3.3 million unsafe abortions and 29, 000 additional maternal deaths during the next 12 months [7-10]. Continuity of essential health services while keeping people safe during the response to disease outbreaks such as the COVID-19 pandemic is therefore essential for the prevention of both direct and indirect mortality [4,5].

This is a generic standardized protocol designed to maximize the likelihood that data are systematically collected and shared rapidly in a format that can be easily reproducible, aggregated, tabulated and analyzed across many different settings globally and be useful as templates for use in health emergencies in the future. This will facilitate the comparison of results across regions and countries and will potentially improve the quality of observational studies by identifying and minimizing biases. Given that use in different settings will require some adaptation, these possibilities have been highlighted throughout the protocol.

The introduction should be updated with country specific data on COVID-19 epidemiology and current research findings prior to submission to local/country national institutional review boards

For peer review only - http://bmjopen.bmj.com/site/about/guidelines.xhtml

The main aim of this study is to assess the availability of contraception, comprehensive abortion care, STI prevention and treatment (including HIV) and SGBV care and support services in local health facilities during COVID-19 pandemic. The four specific objectives of the study are:

1. To explore the availability of, and health facility readiness to provide these services in areas most affected by COVID-19.
2. To assess the availability and quality of services and barriers to the utilization of these services from clients' and providers' perspectives in the selected COVID-19 affected areas.
3. To assess the post-pandemic recovery* of the facilities in the provision of these services in comparison to the pandemic period.
4. To enhance the SRH service capacity in COVID-19 through advocacy, policy briefs, media dissemination and academic papers towards national and regional stakeholders including policymakers, academia, healthcare providers and the community.

**Post-pandemic recovery refers to the period when ideally health facilities have been able to recover from the disruption in service provision, following reduced transmission levels. Caution should be applied to the term "post-pandemic" as services may be still impacted even with lower levels of transmission. Therefore, the research team should be cognizant of the distinct time periods in their facility assessments.*

METHODS AND ANALYSIS

Study design

This is a repeated cross-sectional study, using both quantitative and qualitative methods. The two data collection points, baseline and end-line, will be 9 to 12-months apart. The aim of having two data collection points is to document and share the local evidence with authorities on the SRH services at baseline, and then at end line to track and demonstrate the changes and improvement in services over time. The World Health Organisation (WHO) situational level assessment of COVID-19 transmission will be used as the basis for determining when to conduct baseline and endpoint data collection [11]:

- Level 0: no known transmission in the preceding 28 days, no restriction on daily activities.
- Level 1: basic measures in place, clusters of cases reported controlled with basic measures, limited and transient disease.
- Level 2: local transmission from imported cases to close contacts. Contact tracing, physical distancing and quarantine measures can contain the spread.
- Level 3: community transmission, where source of infection is untraceable, outbreak rapidly spreads in clusters.
- Level 4: disease outbreak has become an epidemic, where there are major clusters of infection all over the country, high number of deaths and it is very difficult to control transmission without strict containment measures.

Countries should time the baseline and end-line data collection as follows: Baseline data collection is rproposed when countries are experiencing level 3 or 4 transmission (referred to here as peak transmission/pandemic period), as disruptions in service provision are likely to occur during these periods. End-line data collection is proposed when countries have tentatively

managed to contain the spread and are at levels 0,1 or 2 (referred to here as post-pandemic period/after the pandemic) after experiencing levels 3 or 4.
Given the dynamic nature of the epidemic, where each country is experiencing varying levels of COVID-19 transmission (and the challenges from the resurgence of variants and sudden increase in the number of cases), it is difficult to assume standardized pandemic conditions at country level. Therefore, the time interval between data collection points is just an estimation. For countries that have contained the spread, it may be decided to collect baseline data retrospectively, selecting the peak period. However, strategies to minimize or avoid recall bias should be used.

Study Setting
The study will be conducted in geographical areas selected based on the epidemic status. To get variation in responses, health facilities will be selected as focal points considering their qualification to provide SRH services and other criteria (see section 3.4) for the researchers to access community members for participation in the study. It is expected that variation in health facilities distributed within different geographical areas will provide access to communities of all socioeconomic backgrounds.

Due to various local realities and political factors, countries should select the research study sites based on geographical location, organization of SRH service delivery and epidemic status where COVID-19 is likely to have significantly affected service delivery (given that even within countries the transmission status differs, hence the differential impact on facilities in different geographical areas). Consultation with the Ministry of Health will help identify the areas most affected by COVID-19.

Study Population
The study population will be women seeking SRH services from the selected health facilities and their partners who accompany them, as applicable, and healthcare providers working there.

In contexts where it is not customary for men to accompany their partners when they seek SRH services, other options can be used to access them. Healthcare providers can be medical doctors, nurses, midwives, nurse assistants, allied health professionals and other cadres depending on the norms and standards for the provision of SRH services in different country contexts.

Sample selection
Each sample will be selected based on specific criteria, as well as specific country contextual factors.

For peer review only - http://bmjopen.bmj.com/site/about/guidelines.xhtml

Health facility selection

The minimum criteria for selection should include availability of human resources, primarily the qualification to provide contraception, safe abortion, including the treatment of complications and the provision of post-abortion care, STI care and treatment and support for women experiencing SGBV. In addition, the diversity of health facility capacity, administrative rank, urban/rural setting and the willingness of the providers in charge to participate in the study, as well as how the COVID-19 response has been organized in terms of treatment centers. These criteria for selection of health facilities within the geographical sites were made to encourage a representative mix of facilities. The same health facilities will be used for both data collection points to highlight the changes in SRH delivery due to COVID-19 and post-pandemic recovery.

Given the dynamic nature of the pandemic *some flexibility should be given to all study sites in the selection of facilities, maintaining the minimum criteria specified above*

Qualitative sample

Selection of women

Women will be recruited for in-depth interviews (IDIs) and focus group discussions (FGDs). The women will be purposively selected to obtain a sample of women and their partners. Selection will be based on 1) being of reproductive age (18-49) and 2) having sought or tried to receive SRH services from local health facilities. The study will use gatekeepers, who will be healthcare provides not involved in the study to approach the women as they leave the health facility. Their role will be limited to the identification and introduction of potential participants to the study team.

For the IDIs, a sample of 10~15 women (or until saturation) and 6~12 partners (or until saturation) will be recruited. For countries with more enrolled health facilities in the study, the maximum number of the IDIs will be with 10 women (or until saturation) at reproductive age seeking for reproductive healthcare services, and with 5 partners (or until saturation) per health facility. This will be done as an exit interview and the women will be consecutively selected until the desired sample (or saturation) has been achieved.

For FGDs, gatekeepers will also ask participants about their level of comfort discussing experience seeking SRH issues in a group before being recruited. About 6-8 participants per FGD will be recruited, with the expectation of at least two focus groups per facility. Some female and male participants will also be invited for individual interviews after the FGDs.

The ages can be adjusted to account for differences in the nationally recognised reproductive age group in different contexts. Inclusion of girls younger than 18 years should take into consideration ethical issues of assent and consent.

To adjust to challenges posed by COVID-19 transmission, additional participants can be recruited through online recruitment from chat groups such as Facebook, Twitter or WhatsApp. Careful

For peer review only - http://bmjopen.bmj.com/site/about/guidelines.xhtml

attention must be paid to the ethical issues of privacy and confidentiality of participants and integrity of the researchers (transparency of aims, details, risks and benefits of the study, obtaining the necessary permission to join restricted groups and the presence of the researcher in the group). Other challenges of online recruitment that need attention include retention of participants, potential selection bias, verification of participant identity and comprehension of informed consent [12]. Care needs to be taken by hiding the names and faces (e.g., via video conference calls) of the online participants, responsible handling of participants' personal information to minimize the likelihood of embarrassment, loss of dignity or harm because of the online recruitment process to address the online privacy and confidentiality concerns. The moderators will ask the participants to give a short self-introduction, using pseudonyms/numbers, instead of their actual names, and not using video options. This includes not disclosing any of this information without the participants consent.

Selection of healthcare providers

Approximately 1 to 2 healthcare providers per health facility will be purposively selected and only those who 1) deliver SRH services, 2) are most knowledgeable about readiness and availability of SRH services 3) are stationed in the SRH clinic and have been working at the clinic for at least 6 months before the pandemic started will be selected for inclusion in the study. This information will be obtained from the facility in-charges.

Quantitative sample

Selection of Women

In each health facility, a maximum of 3 clients from each section (post-abortion care, family planning, SGBV, STI, abortion) will be consecutively selected to achieve up to 20 clients per health facility.

Selection of healthcare providers

One healthcare provider, preferably most knowledgeable in the health facility about SRH services provided, as determined by seniority, position or function, for example, will be selected to assist in the health facility assessment.

Data Collection

FGDs and IDIs will be used to understand client's perspectives on their experiences in accessing SRH services during and after peak transmission and healthcare providers' perspectives of SRH service availability and readiness in COVID-19-affected areas. The health system assessment will be used to assess the health system response to COVID-19. Study teams should be aware of the possibility that healthcare provider burnout or heavy workload may affect willingness or ability to participate in the study. Adaptations to data collection should be made to avoid overburdening the healthcare providers and clearly explained during recruitment.

Data Collection tools

There will be five sets of tools for clients- 1 FGD guide for women, 1 FGD guide for partners, 1

For peer review only - http://bmjopen.bmj.com/site/about/guidelines.xhtml

interview guide for women, 1 interview guide for men (published as **supplementary file S1**) and a health services continuation questionnaire for women (published as part of **supplementary file S3**); and 1 interview guide for healthcare providers (published as **supplementary file S2**).

For the facility assessment, a facility and readiness assessment questionnaire has been developed based on the following validated tools [13-16]:

(1) WHO Service Availability and Readiness Assessment (SARA) guide
(2) WHO Health Facility Readiness Checklist
(3) WHO safe abortion assessment tool
(4) SGBV Secual and gender-based violence Quality Assurance Tool

The facility and readiness assessment tool comprises five modules: (1) health services continuation (; (2) family planning services; (3) abortion services; (4) STI and (5) SGBV; in line with the study SRH focus areas (published as **supplementary file S3**). The following indicators will be used the assess the availability and readiness:

(1) Policies and plans
(2) Service maintenance and referrals
(3) Infrastructure
(4) Commodities
(5) Human resources

Each study site will adapt (including translation to local language) the tool to its specific context, taking into consideration the existing policies, SRH treatment guidelines, staffing norms and standards, types of facilities, national essential medicines lists and national health information systems among others. After adaptation to specific country context all tools should be piloted before being used.

Data Collection Methods

Participant Informed Consent Process

All study participants will be taken through a detailed informed consent process (for participation and audio-recording), which will be documented and those who agree to participate in the study will be asked for signed written (oral consent for those who cannot write, or for situations that necessitate online data collection) consent.

Given that data collection will be conducted during COVID-19, an assessment of the risks to the study team and participants should be made. The decision of whether to conduct face-to-face or remote data collection should be based on the risk assessment, followed by context-specific recommendations on adaptations to the original data collection process. Remote data collection will necessitate adaptation to the length of interviews and discussions, rapport building, oral informed consent, privacy and confidentiality measures as well as making special arrangements where access to internet and mobile connectivity is limited [17]. Face-to-face data collection will require special arrangements to ensure the safety of the study team and participants, following WHO as well as country-specific safety protocols, including educating and training the research

For peer review only - http://bmjopen.bmj.com/site/about/guidelines.xhtml

team and participants about COVID-19, provision of PPE and hygiene supplies, mandatory hygiene practices and sanitization of venues and equipment and physical distancing [18].

The FGDs and IDIs will be conducted at times and in venues that are considered both convenient and safe for the participants to freely discuss the subject matters. In addition, qualified researchers with experience in conducting IDIs and facilitating FGDs will be trained to ensure the validity of the data collection and will be selected based on the gender of the participants. The FGDs and IDIs will be semi-structured and follow a topic guide specific to each group of participants but will take place as a conversation in which the researchers promote a safe, comfortable environment to enable a comprehensive and candid record. In addition to the semi-structured interview, narrative interview techniques may be used depending on the participants' narratives about life events and reproductive healthcare needs.

The FGDs and IDIs will be audio-recorded. Prior consent for recording will be sought again from each participant. Anonymity of the participants will be ensured by removing any personal or family identifiers and all recorded sessions will be coded for purposes of identification with a date, geographical site and session number. After each session, the recording will be sent to the team supervisor for secure storage for transcription. The audio files will be encrypted and sent to an electronic database which will be shared with WHO. Therefore, only the research team will be authorized to listen to the recordings. These audio recordings will be retained until they have been transcribed and checked for accuracy, after which they will be destroyed.

The FGDs will explore knowledge about COVID-19, care-seeking during COVID-19 and risk perceptions and availability of SRH services (end-line FGD will also explore post-pandemic services in comparison to the pandemic period). Before starting the FGD, the facilitators will collect socio-demographic data for each participant, build rapport with and among participants and set ground rules to ensure positive group dynamics that foster an effective and rich discussion. The facilitators will also use this time to know and understand the emic categories (field research and viewpoints obtained from within the social group, from the perspective of the subject) used by locals to describe their perceptions and practices related to the study topics. Each FGD is expected to last approximately 60 – 90 minutes.

IDIs with women will explore the psychosocial effects of COVID-19 on fertility desires, knowledge of COVID-19, risk perceptions and concerns about effects on SRH, care seeking behaviors, experience and barriers in accessing SRH services reproductive health, particularly comprehensive abortion, STI prevention and treatment and SGBV care and support, and their related needs for accurate information and reproductive health services. IDIs with partners will explore their knowledge of COVID-19, risk perceptions and concerns about effects on SRH, their influence on the access to SRH services of their partners and find out what role they play when their partners require SRH services like contraception, comprehensive abortion care, STI prevention and treatment and SGBV care and support. Each interview is expected to last approximately 40 – 60 minutes. IDIs for the women and their partners will be conducted separately.

For peer review only - http://bmjopen.bmj.com/site/about/guidelines.xhtml

In some countries, abortion is illegal, posing challenges in data quality since participants, fearing reprisals, may not provide accurate responses to abortion-related questions. Necessary precautions should be taken to ensure the interviews are conducted in a supportive and non-judgmental manner, encouraging the participants to respond freely. These include selection and training of interviewers to enable them to overcome their biases and stereotypes about abortion, building trust and rapport with participants and selection of interview venue to guarantee privacy [19].

Given the sensitive nature of the data being collected, care must be taken in the selection of data collectors to ensure protection of participants. The gender, experience and attitudes of the data collectors are important considerations. Training of data collectors is essential with emphasis on the risks to the participants and how to protect them, the importance of non-judgmental attitudes and provision of necessary support to participants.

Interviews with healthcare providers will be based on WHO's six building blocks framework [20 with a focus on the delivery of the focus SRH services during COVID-19; their perceptions on the roles and responsibilities of different parties to provide these services in the COVID-19 context; health system capacity to provide good quality of care for people during COVID-19; training needs, attitudes, biases about contraception, abortion, STI and SGBV in the context of COVID-19; and perceived psychosocial effects on men and women, their families and local communities.

The health availability and readiness assessment will be implemented in all the selected facilities using a cross-sectional survey design to highlight gaps or service delivery issues during COVID-19 and recovery in service availability and readiness in the same health facilities. The assessment will also include a review of health facility records in family planning and contraception, STI care and treatment and care and support for women who have experienced SGBV to assess the availability, type and range of commodities offered. A follow-up assessment will be done at end-line. The follow-up survey plans to assess the recovery in service availability and readiness in the same health facilities. The research team will fill out the data extraction tool and the questionnaire with the assistance of the most knowledgeable person in the health facility about SRH services provided (senior health care provider and/or administrator) for each section of the questionnaire. The assessment will also include a questionnaire to the clients of the health facility.

The client questionnaire contains questions related to general characteristics of clients and their experience in seeking SRH services. All the selected health facilities will be required to collect this data from all clients seeking SRH services once every month throughout the study period, to capture trends in service availability and utilization.

Data Analysis

Health facility assessment data will use the presence of the tracer items for the provision of these services, such as availability of guidelines, staff and essential commodities. Data will be entered into an electronic database using data entry programmes such as Epi data and CSPro. Study sites

For peer review only - http://bmjopen.bmj.com/site/about/guidelines.xhtml

using electronic data collection may skip this step and for WHO supported centres implementing the study an online platform (OpenClinica) will be used. Descriptive analysis will be used to illustrate the basic characteristics of the different facilities, including the monthly number of clients, types of procedures provided, number of medical staff, stocks of drugs, etc.

Qualitative analysis will form the main analysis for this study, beginning while data collection is ongoing to assess progress and determine any problems. The audio-recorded data will be transcribed verbatim and de-identified by using ID numbers in place of names. Where required, transcriptions will be translated to English and back-translated and analyzed using content analysis, according to the suggested steps by Elo & Kyngäs (2008) [21], as illustrated in Table 1. The WHO team based in Geneva, in conjunction with the study principal investigator (PI) and project team, will support and monitor the data analysis.

Table 1. Planned analytical procedures for content analysis

Phase	Procedures
Preparation	Reading through verbatim transcriptions of the interviews several times to familiarize with the data, gain an understanding of what has been expressed, selecting the unit of analysis, deciding on the analysis of manifest content.
Organizing	Open coding and creating categories, grouping codes under higher order headings, formulating a general description of the research topic through generating categories and subcategories as abstracting.
Reporting	Reporting the analysis process and the results through models, conceptual systems, conceptual map or categories, and a story line.

Data management and access

Data management plans should include information about how data will be stored, including levels of protection, who will have access to the data and when it would be destroyed, how data will be transferred (if needed) securely. In countries where data protection legislation exists, protocols should specify that data would be handled in accordance with those policies.

Quantitative data

Data collectors will be trained on data collection, transmission, verification, storage and primary analysis to assess errors. Data can be collected electronically, or on paper.

Qualitative data

The quality and trustworthiness of the qualitative data collected will be assured through triangulation, as we will conduct interviews with different parties, including health professionals, health workers, and clients. The process of the study will be clearly documented to ensure the transparency and the rigor of the study.

For peer review only - http://bmjopen.bmj.com/site/about/guidelines.xhtml

Ethics and dissemination

Scientific approval has been obtained from WHO research project review panel (RP2). Ethical approval for this study was also obtained from the WHO Ethics Review Committee (protocol ID CERC.0103). Each study site is required to obtain the necessary ethical and regulatory approvals that are required in the corresponding country. Ethical considerations of informed consent, voluntary participation, privacy and confidentiality, anonymity and compensation for incidental costs (the decision to offer compensation and its value will depend on the specific country context and respective local policies) will be respected and detailed in the informed consent process.

Given the sensitive nature of the data being collected, special care will be taken to ensure the "do no harm" principle is respected. This will include making arrangements with locally available support services (medical, psychosocial, legal) where participants can be referred, training the research team to respond to and provide immediate emotional support to study participants or for situations where support services are not locally available or are inadequate [22, 23].

At country level the results will be presented to policy makers, researchers, managers through policy briefs and workshops in collaboration with the WHO office. The results will also be disseminated among the communities/participants through online platforms/text messages, which will be provided to the participants by the data collectors/health workers. In addition, the results will also be presented to the scientific and funding community in collaboration with WHO country and regional offices, by communication and manuscript publications in peer-reviewed international journals

Discussion

This is a generic standardized protocol designed to be used across many different settings globally and to be useful as templates for use in health emergencies in the future. This will facilitate the comparison of results across regions and countries.

This study provides a unique opportunity to assess the availability, utilization and readiness of the SRH services during the COVID-19 pandemic. The main lesson learned will be the adaptations of the health system in pandemic situations and what can be done to ensure continuity of essential SRH services.

The mixed methods approach and panel design with two data collection points enable a comprehensive analysis of the barriers to availability, utilization and readiness of sexual and reproductive health services during COVID-19, as well as the post-pandemic recovery as transmission is contained.

This study is well placed to advocate for the development and strengthening of policies and services that are responsive to the needs of women and girls during health emergencies, given the potential to exacerbate existing gender and social inequalities and increase the vulnerability of women and girls to preventable health risks. The use of a mainly qualitative approach places women's rights and needs at the center of the debate. The inclusion of partners will enhance

For peer review only - http://bmjopen.bmj.com/site/about/guidelines.xhtml

understanding of gender dynamics and support efforts towards identifying strategies to enhance positive male involvement and engagement in women's sexual reproductive health and rights.

Contributorship statement

Contributors: MA and CRK conceptualized and designed the protocol. All authors contributed to developing the detailed methodology. SK, EC and MA drafted the first draft manuscript. JS, CK, LB, VB, JGC, EM, HK and PL edited the draft, provided critical inputs, and approved the final written version for publication.

Funding Statement

This research has been supported by German Federal Ministry of Health (BMG) COVID-19 Research and development funding to WHO. It has also been supported by the UNDP-UNICEF-WHO-World Bank Special Programme of Research, Development and Research Training in Human Reproduction (HRP); a co-sponsored programme executed by the World Health Organisation (WHO). The adaptation and implementation of this protocol in the HRP collaborating centres in Brazil, Burkina Faso, China, Ghana, Italy, Kenya, Pakistan, Thailand and the United Kingdom was also funded by the same. The views of the funding body have not influenced the content of this manuscript. This article represents the views of the named authors only and does not represent the view of their affiliated institutions.

For peer review only

Disclaimer

This report contains the collective views of an international group of experts and does not necessarily represent the decisions or the stated policy of the World Health Organization.

Data Sharing Statement

Data sharing not applicable as no datasets generated and/or analysed for this protocol.

Competing interests

The authors declare that they have no competing interests.

Patient and public involvement

No patient or public involvement.

Acronyms

COVID-19	Corona Virus Disease 2019
CSPro	Census and Survey Processing System
FGD	Focus Group discussion
SGBV	Gender Based Violence
IDI	In-depth Interview
RP2	Project Review Panel
SRH	Sexual and Reproductive Health
STI	Sexually Transmitted Infections

For peer review only - http://bmjopen.bmj.com/site/about/guidelines.xhtml

WHO World Health Organisation

References

1. Riley T, Sully E, Ahmed Z, Biddlecom A. Estimates of the potential impact of the COVID-19 pandemic on sexual and reproductive health in low- and middle-income countries. *Int Perspect Sex Reprod Health* 2020; 46:73–76. doi: 10.1363/46e9020.
2. Addressing human rights as key to the COVID-19 response. Geneva: World Health Organization; April 2020 (WHO/2019-nCoV/SRH/Rights/2020.1; https://www.who.int/publications/i/item/addressing-human-rights-as-key-to-the-covid-19-response (accessed August 15, 2020)
3. Sexual and Reproductive Health and Rights during the COVID-19 pandemic: A joint report by EPF & IPPF EN. April 2020. https://www.ippfen.org/sites/ippfen/files/2020-04/Sexual and Reproductive Health during the COVID-19 pandemic.pdf (accessed August 16, 2020)
4. COVID-19: operational guidance for maintaining essential health services during an outbreak: interim guidance. 25 March 2020. Geneva: World Health Organization. 2020 WHO/2019-nCoV/essential_health_services/2020.1; https://apps.who.int/iris/ handle/10665/331561 (accessed on August 15, 2020).
5. Maintaining essential health services: operational guidance for the COVID-19 context. World Health Organisation; 2020. https://apps.who.int/iris/handle/10665/332240 (Accessed August 15, 2020)
6. Impact of the COVID-19 pandemic on family planning and ending secual and gender-based violence, female genital mutilation and child marriage: pandemic threatens achievement of the transformative results committed to by UNFPA. New York, United Nations Population Fund; 2020 https://www.unfpa.org/resources/impact-covid-19-pandemic-family-planning-and-endinggender-based-violence-female-genital-mutilation (accessed on August 15, 2020).
7. Millions more cases of violence, child marriage, female genital mutilation, unintended pregnancy expected due to the COVID-19 pandemic [Internet]. Unfpa.org. 2020 [cited 3 May 2020]. Available from: https://www.unfpa.org/news/millions-more-cases-violence-child-marriage-female-genital-mutilation-unintended-pregnancies#
8. Coronavirus Disease (COVID-19) Pandemic UNFPA Response Plan. June 2020 https://www.unfpa.org/sites/default/files/resource-pdf/UNFPA_Global_Response_Plan_Revised_June_2020_.pdf (accessed August 16, 2020)
9. United Nations Population Fund. Impact of COVID-19 on family planning: What we know one year into the pandemic. Technical note. New York, UNFPA;2021
10. Julia Hussein. COVID-19: What implications for sexual and reproductive health and right globally? *Sex Reprod Health Matters* 2020;28(1):1746065 DOI:10.1080/26410397.20201756065.
11. World Health Organization. Considerations for implementing and adjusting public health and social measures in the context of COVID-19. Interim guidance. Geneva, World Health Organization;2020

For peer review only - http://bmjopen.bmj.com/site/about/guidelines.xhtml

https://www.who.int/publications/i/item/considerations-in-adjusting-public-health-and-social-measures-in-the-context-of-covid-19-interim-guidance (Accessed December 5, 2020)
12. Gelinas L, Pierce R, Winkler S, et.al. Using social media as a research recruitment tool: ethical issues and recommendations. *Am J Bioeth 2017*;17(3):3-14
13. World Health Organization. Service availability and readiness assessment (SARA): implementation guide. Geneva: World Health Organization; 2015 http://www.who.int/healthinfo/systems/sara_implementation_guide/en/. (Accessed August 20, 2020)
14. World Health Organization. Safe abortion: technical and policy guidance for health systems. 2nd ed. Geneva: World Health Organization; 2012 http://www.who.int/reproductivehealth/publications/unsafe_abortion/9789241548434/en/. (Accessed March 10, 2021)
15. JHPIEGO. 2018. Secual and gender-based violence Quality Assurance Tool: Standards for the Provision of High-Quality Post-Violence Care in Health Facilities. https://www.who.int/reproductivehealth/publications/post-violence-care-in-health-facilities/en/ (Accessed March 10, 2021)
16. World Health Organisation. Rapid Hospital Readiness Checklist: Harmonized Health Service Capacity Assessment in the Context of the COVID-19 Pandemic. Interim Guidance. Geneva: World Health Organization; 2020 https://apps.who.int/iris/handle/10665/332779
17. Hensen B, Mackworth-Young CRS, Simwinga M, et al. Remote data collection for public health research in the covid-19 era: ethical implications, challenges and opportunities. *Health Policy Plan* 2021;czaa158 https://doi.org/10.1093/heapol/czaa158
18. IMPACT. 2020. SOPs for Data Collection during COVID-19. https://www.reachresourcecentre.info/wp-content/uploads/2020/05/DataCollectionSOPCOVID-19.pdf (Accessed March 10, 2021)
19. Zulu JM, Ali J, Hallex K, et.al. Ethical challenges in research on post-abortion care with adolescents: experiences of researchers in Zambia. *Glob Bioeth* 2018; 31(1):104-119
20. World Health Organisation. Everybody business: strengthening health systems to improve health outcomes: WHO's framework for action. Geneva, World Health Organisation; 2007. https://www.who.int/healthsystems/strategy/everybodys_business.pdf?ua=1 (Accessed August 15, 2020)
21. Elo S, Kyngäs H. The qualitative content analysis process. *J Adv Nurse* 2008;62:107-115.
22. UN Women, World Health Organisation. Violence Against Women and Girls. Data Collection during COVID-19. New York, UN Women; 2020 https://www.unwomen.org/-/media/headquarters/attachments/sections/library/publications/2020/vawg-data-collection-during-covid-19 (Accessed March 10, 2021)
23. World Health Organisation. Putting Women First: Ethical and Safety Recommendations for research on Domestic Violence Against Women. Geneva, World Health Organization;2001 https://apps.who.int/iris/bitstream/handle/10665/65893/WHO_FCH_GWH_01.1.pdf?sequence=1 (Accessed Jan 6, 2021)

For peer review only - http://bmjopen.bmj.com/site/about/guidelines.xhtml

Focused group topic guide for women

Demographic information (Note: Collect data on individuals before the FGD)

- Age (in completed years)
- Education (highest level attained)
- Residence (actual, urban, rural, slum)
- COVID-19 infection history (self, close family, work mates, friends)
- Social economic status - Employment status (i.e. formal or informal employment), income earning status and possibly income ranges as suitable in the country setting.

Knowledge about COVID-19 (such as cause, signs and symptoms)

1. What is COVID-19? What do you know about COVID-19?
   PROBE: for causes, transmission, signs, symptoms, sources of information about COVID-19
2. Did you perceive yourself to be at risk for COVID-19 infection?
   PROMPT: Probe for any special considerations (pregnancy, pre-existing health conditions, others).

Care seeking and perception of risk about being infected by COVID-19

3. The decision-making process of treatment seeking during COVID-19
   PROBE: Ask who influenced the decision to seek care during COVID-19 (self, partner, other family member, etc?)
4. Perception of risk of infection during SRH care seeking.
   PROMPT: How did this affect health seeking behaviour during COVID-19?
5. The treatment-seeking process during COVID-19
   a. Were your needs for accurate information on reproductive health services fulfilled? How? Or Why not? PROBE: information sources: brochures, social media, billboards, other printed media, information on service hours or location where services are offered etc.
   b. What were your needs for reproductive health services? Were these needs fulfilled?
      PROBE: Contraception? Abortion care? STI?
   c. Did you encounter any barriers in your bid to access needed reproductive health services? PROBES: transportation, behaviour of other persons, etc.

   PROMPT: Interviewer should probe for other context specific intermediaries to seeking care. E.g. paperwork for insurance
6. Do you think that reproductive services were readily available to clients who needed them? Specifically, the availability of the reproductive health medicine and medical supplies needed by clients based on the following:
   a. Availability of staff, medicines and supplies for SRH during COVID-19
   b. Schedule of treatments/appointments during COVID-19
      PROMPT: Probe for changes in the delivery of SRH services (specifically for family planning, abortion, post-abortion care and STI care) i.e. appointment-based system introduced.
   c. Interaction between the service provider and the patient was during COVID-19. PROBE: Interactions with different cadres of health workers. Nurses? Midwives? Doctors? Records? OPD staff? Etc.
   d. Was the interaction between service providers and staff described above different from pre-COVID-19 time? How?

7. What were some of the safety measures ensured by the health facility to protect clients from COVID-19 during services
8. Do you think that the COVID-19 had any impact on client protection and privacy during services?
   PROBE: What was done (or could have been) differently?

Post-pandemic recovery of the facilities to provide services in comparison to the pandemic period.

GENERAL COMMENTS: Based on the country situation, the probes for the questions above can be extended to cover the post-pandemic period if applicable.

For peer review only

Focused group topic guide for men

Demographic information

- Age (in completed years)
- Education (highest level attained)
- Residence (actual, urban, rural, slum)
- COVID-19 infection history (self, close family, work mates, friends)
- Social economic status: Employment status (i.e. formal or informal employment), income earning status and possibly income ranges as suitable in the country setting.

Knowledge about COVID-19 (such as cause, signs and symptoms)

1. What is COVID-19? What do you know about COVID-19?
   PROBE: for causes, transmission, signs, symptoms, sources of information about COVID-19
2. Did you perceive yourself to be at risk for COVID-19 infection?
   PROMPT: Probe for any special considerations (pregnancy, pre-existing health conditions, others).

Care seeking and perception of risk about being infected by COVID-19

1. What role did you play in the decision-making process of treatment seeking during COVID-19?
   PROBE: final decision maker (man only, joint, woman only, other); stage of involvement (decision stage or care seeking stage).
2. The role of men in the treatment-seeking process during COVID-19
   a. What role did you play in seeking for accurate information on reproductive health services? Were your needs fulfilled? How? Or Why not?
      PROBE: information sources: brochures, social media, billboards, other printed media, etc. The role of men in looking for sexual reproductive health services (including contraception and abortion care) and whether the needs were fulfilled
   b. What role did you play in looking for reproductive health services? Were your needs for reproductive health services fulfilled?
      PROBE: Contraception? Abortion care? STI?
   c. Did you encounter any barriers in your bid to access needed reproductive health services? PROBES: transportation, behaviour of other persons, entry restrictions for men, etc.
      NOTE: Interviewer should probe for other context specific intermediaries to seeking care. E.g. paperwork for insurance
3. Do you think that reproductive services were readily available to clients who needed them? The availability of the reproductive health medicine and medical supplies needed by clients?
   a. The schedule of treatments/appointments during COVID-19?
4. How was the interaction between the service provider and you as the male partner during COVID-19?
   PROBE: Interactions with different cadres of health workers. Nurses? Midwives? Doctors? Records? OPD staff? Etc
5. Was the interaction between service providers and staff described above different from pre-COVID-19 time? How?

For peer review only

6. What were some of the safety measures ensured by the health facility to protect clients from COVID-19 during services?
   PROBE: Were male partners stopped from entering consulting and treatment rooms due to COVID-19?
7. Do you think that the COVID-19 had any impact on client protection and privacy during services?
8. **PROBE**: What was done (or could have been) differently? E.g. barriers in seeking SRH services during COVID

Post-pandemic recovery of the facilities to provide services in comparison to the pandemic period.

COMMENT: Based on the country situation, the probes for the questions above can be extended to cover the post-pandemic period if applicable.

For peer review only

<u>In-depth Interview topic guide for women</u>

1. Demographic information
   - Age
   - Ethnicity
   - Marital status
   - Socio-economic status
   - Occupation
   - Education status
   - Religion
   - Place of residence and housing condition
   - Obstetric characteristics (no. of pregnancies, parity)

2. Knowledge about COVID-19
   - Cause (sexual transmission)
     - How do you think COVID-19 virus is transmitted?
     - Do you think it can be transmitted sexually?
   - Signs and symptoms of COVID-19
     - What are the sign and symptoms of COVID-19?
     - If yes, can you mention some that you know?
   - Care-seeking for COVID-19 infection
     - Do you know what to do/who to contact/ where to go if you experience COVID-19 signs and systems?
     - If yes, can you explain?
   - Sources of information and trusted communication channels (v. rumors, misinformation) Experience of COVID-19 (direct / indirect)
     - Where did you get information about COVID-19?
     - Was the information you obtained accurate?
     - Can you give me an example of accurate information you obtained?
     - Were there any rumors and/or misinformation about COVID-19?
     - How did you know that this information was just rumors or misinformation?
     - Have you fund sources of information that you can trust?
     - How did you know which information sources to trust?
     - Have you been infected by COVID-19?
     - Do you know of anyone who has been infected? If yes, was it a close friend or family member?
   - Perception of risk about being infected by COVID-19
     - Do you think you are at risk of being infected by the COVID-19 virus?
     - If no, can you tell me why not?
     - Do you think you can be infected whilst seeking care?
   - Ways to prevent being infected by COVID-19 (probe at individual, household, community levels)
     - Do you know how to prevent infection from COVID-19?
     - What can individuals do? What can households do? What can communities do?
   - Responsibility to prevent COVID-19 (probe at individual, household, community levels, authorities)
     - Who is responsible for the prevention of COVID-19 infection? Do you think this

For peer review only

responsibility was fulfilled by individuals, households, communities and authorities? How?

- Religious / socio-cultural practices and influencers
  - Are there any religious / socio-cultural practices that are used to prevent COVID-19 infection?
  - Does participation in any religious / socio-cultural practices make you practice prevention of COVID-19 differently from what is promoted by health professionals?

3. Sexual and Reproductive Health
   - Decision-making and agency to act
     - Who makes the decisions related to your sexual and reproductive health needs (self, husband/partner or family member)?
     - Who is usually responsible for carrying out the decision?
   - Issues of access / utilization
     - Did you need to seek SRH services during COVID-19?
     - If yes, were you able to access the services? How did you access the services?
     - If no, what were the barriers? What did you do?
   - Challenges faced
     - Are there any other challenges you faced in seeking SRH services during COVID-19? What are they and how did you address/manage the challenges?

4. Contraceptive practices
   - Changes in practice (if any) due to COVID-19
     - Which method of contraception do you normally use?
     - Did you have to change your normal method of contraception during COVID-19? If yes, which method and what influenced the change?
     - If no, why didn't you change from your normal practice?
   - Is pregnancy being avoided / delayed due to COVID-19? If yes, why?
   - Access to / utilization of different methods of contraception (barriers / drivers)
   - Which methods of contraception were accessible to you during COVID-19?
   - Was there a difference in the methods of contraception accessible to you compared to before COVID-19?
   - What was causing the difference?
   - Challenges in access / utilization
   - Are there any other challenges you faced in accessing/utilizing contraception services during COVID-19?
   - If yes, what were these challenges and how did you cope with them?

5. Induced abortion
   - Perceptions about abortion at normal times
   - The role abortion plays in women's lives
     - Why do you think women seek abortion services?
     - Do you think abortion is an essential service for women? If yes, in what way?
   - Fear of stigma
     - How does family, community and society view women who seek or have sought abortion services?

- Does this stigma affect your decision whether to have an abortion?
- Does this stigma affect how you will seek abortion services? If yes, how?
- What do you think are the effects of this stigma on women who need abortion services?

- Inequities in access to safe abortion
  - Do you think safe abortion services are equally accessible to all women who need them?
- Perceptions about abortion during COVID-19
  - Would you choose to seek the abortion services during COVID-19, if you need one?
  - If no, why not? If yes, where and how would you access the service?
- Access to different methods of abortion (barriers / drivers)
  - Did you seek abortion services during COVID-19? If yes, which abortion methods were available for you (medical abortion or surgical abortion)?
  - What pushed you to have an abortion? If you were given a choice of methods, which one did you choose and why?
  - Did you face any barriers in seeking and accessing abortion services during COVID-19?
- Challenges in accessing / utilizing safe abortion services (inequities in access)
  - Are there any other challenges you faced in accessing/utilizing safe abortion services? If yes, what were the challenges and how did you address them?

- 9. Violence against women (*Interviewer should use the term that is used locally to describe VAW)*

- Formal and informal systems of support, and coping strategies for VAW
  - Have you or any woman you know experienced any form of VAW?
  - What are some of the systems of support available for women who have experienced VAW?
  - Do you know where and how to access this support?
  - What are some of the coping strategies women who have experience such violence use?
- Perceptions of safety in home and in community safety/increased risk during COVID-19
  - Do you think are safer or at increased risk of experiencing VAW at home or in the community during COVID-19?
  - What has made it safer/riskier for women during COVID-19?
- Women's expectations of services for gender-based violence
  - If you or any other woman experiences GBV during COVID-19, do you expect the care and support services to be available?
  - Do you know where and how to access these services?
  - Do you expect any barriers to accessing these services during COVID-19? If yes, what kind of barriers?
- What are additional stress factors (e.g. school-age children at home, older parents, loss of job/income, partner loss of job, etc.) that increase the likelihood of VAW?
- How does stress affect frequency and severity of violence, particularly during COVID-19?

For peer review only

6. Sexually transmitted infections *(interviewer should use the locally known terms for STIs and the specific diseases)*
   - Perceptions about STI
     - Is anybody at risk of getting a STI? Why or why not?
     - How are people who are known to have a STI perceived in the community?
   - Experience of STI during COVID-19
     - Have you required care and treatment for a STI during COVID-19? If yes, where and how did you seek this service?
     - Were there any barriers to accessing this service? If yes, which ones and what did you do?
     - If no, would you seek care and treatment if required during COVID-19?
     - If no, why not? If yes, where and how would you access this service?

7. Psycho-social
   - Psychosocial issues associated with being infected with COVID-19 (probe: more significant for women/men, more significant for pregnant women)
     - Did you experience any stress, anxiety or depression during COVID-19? If yes, at what level (mild, moderate, severe)?
     - What do you think led to the psychosocial issues you experienced? Was it due to being infected or other related issues or both?
     - Did your partner also experience any psychosocial issues during COVID-19?
     - Who was most affected, yourself or your partner?

8. Final questions
   - On-going concerns about COVID-19 and its possible impact
     - Are there any other concerns about COVID-19 and its impact on you, your family or in general that you wish to express?
   - Needs related to accurate information (probe further information needs – NB consider how to provide information if necessary)
     - Was accurate information readily available and accessible during COVID-19?
     - Was it easy to understand?
     - Was the information that was available adequate?
     - Was there additional information you would have liked to obtain but was missing?
     - Are there better ways of making accurate information accessible to you and others?
   - Needs related to sexual reproductive health services (probe further SRH needs – NB consider how to support/provide if necessary)
   - Do you think SRH services were responsive to your needs during COVID-19?
   - If no, which of your needs were not met?
   - Which SRH services are most important for you, that should be available even in health emergencies like COVID-19?
   - How do you think provision of SRH services can be supported during other health emergencies like COVID-19?

For peer review only

Additional themes will be added as appropriate for each target group and questions revised accordingly (with attention to first person / third person views), and across the three phases of the study.

For peer review only

In-depth Interview topic guide for men

1. Demographic information
   - Age
   - Ethnicity
   - Marital status
   - Socio-economic status
   - Occupation
   - Education status
   - Religion
   - Place of residence and housing condition

2. Knowledge about COVID-19
   - Cause (sexual transmission)
     - How do you think COVID-19 virus is transmitted?
     - Do you think it can be transmitted sexually?
   - Signs and symptoms of COVID-19
     - What are the sign and symptoms of COVID-19?
     - If yes, can you mention some that you know?
   - Care-seeking for COVID-19 infection
     - Do you know what to do/who to contact/ where to go if you experience COVID-19 signs and systems?
     - If yes, can you explain?
   - Sources of information and trusted communication channels (v. rumors, misinformation) Experience of COVID-19 (direct / indirect)
     - Where did you get information about COVID-19?
     - Was the information you obtained accurate?
     - Can you give me an example of accurate information you obtained?
     - Were there any rumors and/or misinformation about COVID-19?
     - How did you know that this information was just rumors or misinformation?
     - Have you fund sources of information that you can trust?
     - How did you know which information sources to trust?
     - Have you been infected by COVID-19?
     - Do you know of anyone who has been infected? If yes, was it a close friend or family member?
   - Perception of risk about being infected by COVID-19
     - Do you think you are at risk of being infected by the COVID-19 virus?
     - If no, can you tell me why not?
     - Do you think you can be infected whilst seeking care?
   - Ways to prevent being infected by COVID-19 (probe at individual, household, community levels)
     - Do you know how to prevent infection from COVID-19?
     - What can individuals do? What can households do? What can communities do?
   - Responsibility to prevent COVID-19 (probe at individual, household, community levels, authorities)
     - Who is responsible for the prevention of COVID-19 infection? Do you think this responsibility was fulfilled by individuals, households, communities and

For peer review only

authorities? How?

- Religious / socio-cultural practices and influencers
  - Are there any religious / socio-cultural practices that are used to prevent COVID-19 infection?
  - Does participation in any religious / socio-cultural practices make you practice prevention of COVID-19 differently from what is promoted by health professionals?

3. Sexual and Reproductive Health
   - Decision-making and agency to act
     - Who makes the decisions related to the sexual and reproductive health needs of your partner (you, partner or family member)?
     - Who is usually responsible for carrying out the decision?
   - Issues of access / utilization
     - Did your partner need to seek SRH services during COVID-19?
     - If yes, was your partner able to access the services? How did your partner access the services?
     - If no, what were the barriers? What did you and/or your partner do?
     - What role did you play in ensuring your partner sought and/or accessed the needed SRH services?
   - Challenges faced
     - Are there any other challenges your partner faced in seeking SRH services during COVID-19? What are they and how did you and/or your partner address/manage the challenges?

4. Contraceptive practices
   - Changes in practice (if any) due to COVID-19
     - Which method of contraception do you and/or your partner normally use?
     - Did you have to change your normal method of contraception during COVID-19? If yes, which method and what influenced the change?
     - If no, why didn't you change from your normal practice?
   - Did your partner avoid / delay pregnancy due to COVID-19? If yes, why?
   - Access to / utilization of different methods of contraception (barriers / drivers)
   - Which methods of contraception were accessible to you and your partner during COVID-19?
   - Was there a difference in the methods of contraception accessible to you compared to before COVID-19?
   - What was causing the difference?
   - Challenges in access / utilization
   - Are there any other challenges you and your partner faced in accessing/utilizing contraception services during COVID-19?
   - If yes, what were these challenges and how did you cope with them?
   - What role did you play in ensuring your partner sought and/or accessed contraception services?

5. Induced abortion

For peer review only

- Perceptions about abortion at normal times
- The role abortion plays in women's lives
  - Why do you think women seek abortion services?
  - Do you think abortion is an essential service for women? If yes, in what way?
- Fear of stigma
  - How does family, community and society view women who seek or have sought abortion services?
  - Does this stigma affect your decision whether to have an abortion?
  - Does this stigma affect how you will seek abortion services? If yes, how?
  - What do you think are the effects of this stigma on women who need abortion services?
- Inequities in access to safe abortion
  - Do you think safe abortion services are equally accessible to all women who need them?

- Perceptions about abortion during COVID-19
  - Would you choose to seek the abortion services during COVID-19, if your partner needed one?
  - If no, why not? If yes, where and how would you access the service?
- Access to different methods of abortion (barriers / drivers)
  - Did your partner seek abortion services during COVID-19? If yes, which abortion methods were available (medical abortion or surgical abortion)?
  - What pushed you and your partner to have an abortion? If you were given a choice of methods, which one did you choose and why?
  - Were there any barriers in seeking and accessing abortion services during COVID-19?
- Challenges in accessing / utilizing safe abortion services (inequities in access)
  - Are there any other challenges you faced in accessing/utilizing safe abortion services? If yes, what were the challenges and how did you address them?
- What was your role in ensuring your partner accessed the abortion service?

6. Violence against women (*Interviewer should use the term that is used locally to describe VAW)*
   - Formal and informal systems of support, and coping strategies for VAW
     - Has your partner or any woman you know experienced any form of VAW?
     - What are some of the systems of support available for women who have experienced VAW?
     - Do you know where and how to access this support?
     - What are some of the coping strategies that women who have experienced such violence use?
   - Perceptions of safety in home and in community safety/increased risk during COVID-19
     - Do you think women are safer or at increased risk of experiencing VAW at home or in the community during COVID-19?
     - What has made it safer/riskier for women during COVID-19?
   - Women's expectations of services for gender-based violence

For peer review only

- If your partner experiences GBV during COVID-19, do you expect the care and support services to be available?
- Do you know where and how to access these services?
- Do you expect any barriers to accessing these services during COVID-19? If yes, what kind of barriers?
- What do you perceive as your role in ensuring your partner get the care and support needed?

- What are additional stress factors (e.g. school-age children at home, older parents, loss of job/income, partner loss of job, etc.) that increase the likelihood of VAW?
- How does stress affect frequency and severity of violence, particularly during COVID-19?

6. Sexually transmitted infections *(interviewer should use the locally known terms for STIs and the specific diseases)*
   - Perceptions about STI
     - Is anybody at risk of getting a STI? Why or why not?
     - How are people who are known to have a STI perceived in the community?
   - Experience of STI during COVID-19
     - Has your partner required care and treatment for a STI during COVID-19? If yes, where and how did you seek this service?
     - Were there any barriers to accessing this service? If yes, which ones and what did you do?
     - If no, would you seek care and treatment if required during COVID-19?
     - If no, why not? If yes, where and how would you access this service?
     - What was your role in ensuring your partner accessed the care and treatment?

7. Psycho-social
   - Psychosocial issues associated with being infected with COVID-19 (probe: more significant for women/men, more significant for pregnant women)
     - Did you experience any stress, anxiety or depression during COVID-19? If yes, at what level (mild, moderate, severe)?
     - What do you think led to the psychosocial issues you experienced? Was it due to being infected or other related issues or both?
     - Did your partner also experience any psychosocial issues during COVID-19?
     - Who was most affected, yourself or your partner?

8. Final questions
   - On-going concerns about COVID-19 and its possible impact
     - Are there any other concerns about COVID-19 and its impact on you, your family or in general that you wish to express?
   - Needs related to accurate information (probe further information needs – NB consider how to provide information if necessary)
     - Was accurate information readily available and accessible during COVID-19?
     - Was it easy to understand?
     - Was the information that was available adequate?
     - Was there additional information you would have liked to obtain but was

For peer review only

missing?
- Are there better ways of making accurate information accessible to you and others?

- Needs related to sexual reproductive health services (probe further SRH needs – NB consider how to support/provide if necessary)
- Do you think SRH services were responsive to your needs during COVID-19?
- If no, which of your needs were not met?
- Which SRH services are most important for you, that should be available even in health emergencies like COVID-19?
- How do you think provision of SRH services can be supported during other health emergencies like COVID-19?

Additional themes will be added as appropriate for each target group and questions revised accordingly (with attention to first person / third person views), and across the three phases of the study.

For peer review only

In-depth Interview topic guide for SRH healthcare providers

Demographic information

- Age
- Ethnicity
- Marital status
- Socio-economic status
- Occupation
- Education status
- Religion

Health facility service availability and readiness

Health service delivery

1. Mobilization of resources for the diagnosis and the treatment of COVID-19 patients by the *(insert Country name*) government happened during COVID-19. Did the mobilization involve your facility? How was the facility involved? (Probe community, regional or national level involvement)

   COMMENT: *Indicate if facility was a designated COVID-19 treatment Centre*
2. What was the emergency response plan of your facility during the outbreak?
3. How did the mobilization of resources towards COVID-19 patients impact the service delivery in your facility, including the impact on human resources, space, medical supplies, etc.?
4. Did the mobilization of resources negatively or positively impact service delivery in your facility? Elaborate
5. Was there a set of essential Sexual & Reproductive Health services outlined to be maintained during the lockdown phase of the COVID-19 pandemic?
   PROMPT: Ask for each of the following services – family planning, abortion, post-abortion care, care and support for GBV and STI prevention and treatment
6. Were there disruptions to any of the SRH essential services outlined? What was the nature (services suspended, limited or shifted) of disruptions observed for each of these essential services (if any)?
   PROBES: for causes (lack of personnel, equipment, medicines or reduced demand, physical space), changes in usual service provision (introduction of appointment-based systems, telemedicine, shifting of usual location) and level of disruption (partial/ complete/not disrupted)
7. Do you think the delivery of SRH services during COVID-19 adequately responded to the needs of the clients at that time?
   PROBE: in what way?
8. What has the recovery process been like and what was the turning point?
   NOTE: Only for applicable countries.
   PROBE for coping mechanisms or approaches to ensuring SRH service delivery.

Health workforce （Human resources）

9. How did COVID-19 affect the health status of health workforce, such as doctors, nurses, ambulance drivers and hospital managers etc.? What measures were taken to protect them from the effects of the epidemic?

For peer review only

PROBE: for the provision of PPE, type of PPE, adequacy of supply and frequency of supply.

10. Was there any form of training on COVID-19? PROMPT: Probe for details of the training if provided – What areas of COVID-19? What form did this training take? Who received this training?
11. Were there employees infected? If someone was infected, how did the infection happen? What was the impact on service delivery due to the infection of SRH workers? How did it influence the service delivery?

> **COMMENT***: In facilities where SRH staff also provide general care in health facility, ask for the impact of an infected clinical staff in the facility even if not SRH-specific*

12. While knowing that they were at higher risk of contracting the virus, did female workers still have to fulfil the obligations such as caring for elders and immediate family (such as children or young dependents)? Is there any other conflict or challenge they faced during COVID-19? Note: Probe for any challenge worth considering in current setting.

Health information system

13. How does the reporting system for infectious diseases run?
    PROMPT: Probe for reporting system for COVID-19.
14. How did this system influence the availability of services in SRH facilities?
15. How did you keep information about the COVID-19 and SRH services flowing during the outbreak with your upper-level medical institutions and potential clients in your community?
    PROBE: how were changes in the delivery of SRH services communicated to clients? How was important COVID-19 information communicated to clients?

Access to essential medicines

16. Was the access to essential medicines, including regular medicines such as birth control pills, contraceptive methods impacted because of the lockdown/shutdown of the city during the COVID-19?

> ***COMMENT****: Shutdown can be replaced with lockdown/restrictions depending on what the COVID-19 response was in country using this guide*

PROBE: ask about medicines for all the study focus SRH services

17. How has the outbreak affected the access to essential medicines in SRH for the patients and the general population?

PROBE: for each- contraceptive methods, abortion, post abortion care, STI prevention and treatment and GBV care and support

COMMENT: Probe separately for routine medication (such as antibiotics, analgesics etc where applicable)

Health systems financing

18. How did the COVID-19 epidemic affect the availability of funds at your facility?
    PROMPT: Inquire about effect on the financing and maintenance of essential SRH services and possible disruptions due to the pandemic, availability of additional government funding allocated to assuring essential health services.

For peer review only

***COMMENT:** This section should be informed by the existing health financing policy (i.e. National health insurance, existing SRH related exemptions and waiver systems) or COVID-19 specific directives on financing.*

Leadership and governance

19. What are your perceptions on the roles and responsibilities of providing SRH services during outbreaks like COVID-19?
    PROBE: where does the primary responsibility to ensure continuity of services lie? Who are the enablers, implementers?
20. How did your facility respond to the emergency and manage to ensure the SRH-related service delivery in your facility?
    PROMPT: Probe for country-specific plan, policies for maintaining SRH service provision during the pandemic.
    PROBE: separately for, family planning, STI and abortion services, services prioritized by the facility during the pandemic.

Health service provider perceptions on the delivery of specific SRH services in the context of COVID-19

A. Gender based Violence

Perceptions of changes in the nature and magnitude of violence against women, and especially domestic violence during COVID-19

- Have you noticed any change in the number of women coming into the health facility reporting gender-based violence since the COVID-19 crisis began?
    - Follow up: Increase or decrease?
    - Follow up: Have the women coming in who report experiencing violence mostly been returning patients, or mostly been new patients?
    - Follow up: Have you noticed an increase in the frequency of abuse that women are experiencing since movement restrictions were put in place?
    - If possible, specify types of violence (physical, sexual, psychological by a partner), non-partner sexual abuse, other

- Do you think that the violence women have been experiencing has become more severe during the crisis, for example has there been an increase in injuries? Violence during pregnancy?
    - Follow up: Have the types of violence that women are reporting changed? How so?

Nature of service provision during COVID-19, whether services for VAW are being changed or adapted and if safety considerations are adequately addressed?

- Since the start of COVID-19, has your health facility changed or adapted services for survivors 'of violence?
    - Probe: (if not mentioned) In some health centers, providers have maintained 'case management' using video calls, or text messages. Did your health center make use of any new technologies to maintain services? If so, did you develop a protocol to ensure safety of survivors?

For peer review only

Access to /use of health services for VAW and related challenges

- Some health centers have had to deal with shortages of personal protective equipment and medications that have impacted their ability to offer services during the COVID-19 crisis. Have there been any new barriers to delivering services to survivors at your health facility?
    - What are these barriers and what approaches are being used to overcome these barriers?

- In a lot of places, external referral systems have also been impacted by the crisis, such as lower numbers of available spaces in shelters due to the need for physical distancing, or NGOs that have been temporarily shut down. To your knowledge, have any changes have been made to your external referral systems? What are these?
    - Follow up: Are any of the services to which you typically refer survivors available in a more limited capacity, or no longer available due to the crisis?

Attitudes towards provision of SRH services in the context of COVID-19

- Do you think it is essential to provide services for VAW during COVID-19? Why do you think so?

B. <u>Induced abortion</u>

Perceptions of changes in the demand for abortion during COVID-19.

- Have you noticed any change in the number of women seeking abortion services at your facility during COVID-19?
    - Follow up: Increase or decrease?
    - Follow-up: any changes in the abortion methods requested?
    - What do you think were the factors driving this change?

Delivery of safe abortion services during COVID-19?

- Where and how can people seek contraceptive services during COVID-19?
    - How many methods of abortion were available in your health facility during COVID-19? Which method of abortion did you suggest during COVID-19? Why?
    - What challenges did you meet in providing safe abortion services during COVID-19? What approaches did you use to overcome these challenges?

Attitudes towards provision of SRH services in the context of COVID-19

- Do you think it is essential to provide safe abortion services during COVID-19? Why do you think so?

C. <u>Contraceptive services</u>

Perceptions of changes in the demand for contraception services during COVID-19.

- Have you noticed any change in the number of women seeking contraception services at your facility during COVID-19?
    - Follow up: Increase or decrease?
    - Follow-up: any changes in the contraception methods requested?
    - What do you think were the factor driving this change?

Delivery of contraception services during COVID-19?

- Where and how can people seek contraceptive services during COVID-19?

For peer review only

- How many kinds of contraceptive methods were available in your health facility during COVID-19?
- What are the new/ altered ways by which contraceptive services are provided as a response to COVID-19?
- What challenges did you meet in providing contraceptive services during COVID-19? What approaches did you use to overcome these challenges?

Attitudes towards provision of SRH services in the context of COVID-19

- Do you think it is essential to provide contraception services during COVID-19? Why do you think so?

D. <u>Prevention and treatment for sexually transmitted infections</u>

Perceptions of changes in the demand for care and treatment for STI during COVID-19.

- Have you noticed any change in the number of women seeking care and treatment for STI at your facility during COVID-19?
  - Follow up: Increase or decrease?
  - What do you think were the factor driving this change?

Availability of STI care and treatment during COVID-19

- Where and how can people seek care and treatment for STI during COVID-19?
  - What are the new/ altered ways by which care and treatment for STI services are provided as a response to COVID-19?
  - Were all the medicines and supplies for providing the care and treatment available during COVID-19?
  - What challenges did you meet in providing care and treatment for STI services during COVID-19? What approaches did you use to overcome these challenges?

Attitudes towards provision of SRH services in the context of COVID-19

- Do you think it is essential to provide prevention and treatment for STIs during COVID-19? Why do you think so?

3. Perceived psychosocial effects of COVID-19
   - Did you experience stress/anxiety/depression during COVID-19?
   - How would rate your psychosocial health?
   - What role did COVID-19 play in influencing your psychosocial health?
   - In what way?
   - What do you think is the psychosocial impact of COVID-19 on people?
   - Do you think the impact was the same for men and women? If yes, why?
   - If no, in what ways is the psychosocial impact different?
   - What do you think is the psychosocial impact of COVID-19 on families?
   - What do you think is the psychosocial impact of COVID-19 on communities?

4. Final questions
   - On-going concerns about COVID-19 and its possible impact
     - Are there any other concerns about COVID-19 and its impact on you, your family or in general that you wish to express?
   - Needs related to accurate information (probe further information needs – NB consider how to provide information if necessary)

For peer review only

- Was accurate information readily available and accessible during COVID-19?
- Was it easy to understand?
- Was the information that was available adequate?
- Was there additional information you would have liked to obtain but was missing?
- Are there better ways of making accurate information accessible to you and others?

- Needs related to sexual reproductive health services (probe further SRH needs – NB consider how to support/provide if necessary)
- Do you think SRH services were responsive to the needs of patients during COVID-19?
- If no, which of needs were not met?
- Which SRH services are most important, that should be available even in health emergencies like COVID-19?
- How do you think provision of SRH services can be supported during other health emergencies like COVID-19? E.g. barriers in seeking SRH services during COVID

Additional themes will be added as appropriate for each target group and questions revised accordingly (with attention to first person / third person views), and across the three phases of the study.

For peer review only

BMJ Open

Health systems analysis and evaluation of the barriers to availability, utilization and readiness of sexual and reproductive health services in COVID-19 affected areas: A WHO mixed methods study protocol

Journal:	*BMJ Open*
Manuscript ID	bmjopen-2021-057810.R3
Article Type:	Protocol
Date Submitted by the Author:	04-Apr-2022
Complete List of Authors:	Kouanda, Seni; Institut de Recherche en Sciences de la Sante; Institut Africain de Sante Publique<br>Nahyuha Chomi, Eunice; Research Institute of Health Sciences<br>Kim, Caron; World Health Organization, Department of Sexual and Reproductive Health and Research. Preventing Unsafe Abortion Team<br>Jen, Sothornwit; Khon Kaen University, Department of Obstetrics and Gynaecology<br>Bahamondes, Luis; State University of Campinas – UNICAMP, Obst & Gynecol<br>Cecatti, Jose; University of Campinas, Obstetrics and Gynecology<br>Lumbiganon, Pisake; Khon Kaen University, Faculty of Medicine<br>Emefa, Modey; University of Ghana<br>Brizuela, Vanessa; WHO, Department of Reproductive Health and Research<br>Kuganantham, Hamsadvani; World Health Organization, Department of Reproductive Health and Research<br>Seuc, Armando; World Health Organization<br>Ali, Moazzam; Organisation mondiale de la Sante,
**Primary Subject Heading**:	Public health
Secondary Subject Heading:	Health policy, Health services research, Infectious diseases
Keywords:	COVID-19, PUBLIC HEALTH, Reproductive medicine < GYNAECOLOGY

SCHOLARONE™
Manuscripts

Manuscript Protocol.

Health systems analysis and evaluation of the barriers to availability, utilization and readiness of sexual and reproductive health services in COVID-19 affected areas: A WHO mixed methods study protocol

- Kouanda, Seni-Reproductive Health Unit, Institute for Research in Health Sciences, Ouagadougou, Burkina Faso
- Chomi, Eunice- Reproductive Health Unit, Institute for Research in Health Sciences, Ouagadougou, Burkina Faso
- Kim, Caron- Department of Reproductive Health and Research, World Health Organization, Avenue Appia 20, 1211 Genève 27, Switzerland
- Sothornwit, Jen- Department of Obstetrics and Gynaecology, Faculty of Medicine, Khon Kaen University, Khon Kaen, Thailand.
- Bahamondes, Luis- School of Medical Sciences, University of Campinas (UNICAMP), Campinas, Brazil
- Cecatti, Jose Guilherme- School of Medical Sciences, University of Campinas (UNICAMP), Campinas, Brazil
- Lumbiganon, Pisake- Faculty of Medicine, Khon Kaen University, Khon Kaen, Thailand
- Modey, Emefa-Department of population, family and Reproductive Health, University of Ghana, Legon-Accra, Ghana.
- Brizuela, Vanessa- Department of Reproductive Health and Research, World Health Organization, Avenue Appia 20, 1211 Genève 27, Switzerland
- Kuganathan, Hamsa- Department of Reproductive Health and Research, World Health Organization, Avenue Appia 20, 1211 Genève 27, Switzerland
- Seuc, Armando Humberto, Consultant, Department of Reproductive Health and Research, World Health Organization, Avenue Appia 20, 1211 Genève 27, Switzerland
- Ali, Moazzam* on behalf of WHO HRP social Science Research Team**- Department of Reproductive Health and Research, World Health Organization, Avenue Appia 20, 1211 Genève 27, Switzerland

*Corresponding author: Moazzam Ali (alimoa@who.int). Telephone: +41-(0)22-791-3442
Department of Reproductive Health and Research, World Health Organization, Avenue Appia 20, 1211 Genève 27, Switzerland.

**** WHO HRP Social Science Research Team**: **Brazil** (Luis Bahamondes, Jose Guilherme Cecatti); **Burkina Faso** (Eunice Chomi, Seni Kouanda); **China** (Kun Tang, Hanxiyue Zhang, Yifan Zhu, Yueping Guo, Ge Yang and Chunxiao Peng); **Ghana** (Deda Ogum Alangea, Kwasi Tropsey, Emefa Judith Modey); **Pakistan** (Rozina Karmaliani, Laila Ladak); **Thailand** (Pisake Lumbiganon, Jen Sothornwit); **Kenya** (Marleen Temmerman, Abdu Mohiddin, Ferdinand Okwaro); **Italy** (Massimo Mirandola, Maddalena Cordioli, Alessia Savoldi, Simone Garzon, Stefano Uccella, Ranieri Poli.) **UK** (Nigel Sherriff, Alexandra Sawyer, Jorg Huber, Jaime Vera, Debbie Williams).

For peer review only - http://bmjopen.bmj.com/site/about/guidelines.xhtml

WHO Secretariat (Moazzam Ali, Caron Kim, Hamsadvani Kuganantham, Igor Toskin, Vanessa Brizuela, Anna Thorson, Joy Jerop Chebet, Hugo Gamerro Abrego , Soe Soe Thwin, Armando Seuc)

Word count of text (excluding title page, abstract, references, figures and tables)- 4345

KEYWORDS
Contraception, sexual and reproductive health, abortion services, COVID-19, facility assessment, sexual and gender-based violence, sexually transmitted infections

For peer review only

Abstract

Introduction The novel Corona Virus Disease 2019 (COVID-19) has led to an unprecedented increase in demand on health systems to care for people infected, necessitating the allocation of significant resources, especially medical resources, towards the response. This, compounded by the restrictions on movement instituted may have led to disruptions in the provision of essential services, including sexual and reproductive health (SRH) services. This study aims to assess the availability of contraception, comprehensive abortion care, sexually transmitted infection (STI) prevention and treatment and sexual and gender-based violence (SGBV) care and support services in local health facilities during COVID-19 pandemic. This is a standardized generic protocol designed for use across different global settings.

Methods and Analysis This study adopts both quantitative and qualitative methods to assess health facilities' SRH service availability and readiness, and clients' and providers' perceptions of the availability and readiness of these services in COVID-19-affected areas. The study has 2 levels: (1) Perceptions of clients (and their partners) and healthcare providers, using qualitative methods, and (2) assessment of infrastructure availability and readiness to provide SRH services through reviews, facility service statistics for clients and a qualitative survey for healthcare provider perspectives. The health system assessment will use a cross-sectional panel survey design with two data collection points to capture changes in SRH services availability as a result of the COVID-19 epidemic. Data will be collected using focus group discussions, in-depth interviews and a health facility assessment survey.

Ethics and Dissemination Ethical approval for this study was obtained from the World Health Organisation (WHO) Scientific and Ethics Review Committee (protocol ID CERC.0103). Each study site is required to obtain the necessary ethical and regulatory approvals that are required in each specific country.

Strengths and limitation of this study

- The study approach and design enable a comprehensive analysis of the barriers to availability, utilization and readiness of sexual and reproductive health services during COVID-19, as well as the post-pandemic recovery as transmission is contained.
- The use of a mainly qualitative approach places women's rights and needs during health emergencies at the center of the debate, underscoring the need for more responsive policies.
- The inclusion of partners will enhance understanding of gender dynamics and support efforts towards identifying strategies to enhance positive male involvement and engagement in women's sexual reproductive health and rights.
- A possible limitation is that the study will be conducted during and after one of the waves of the pandemic, at which stage there may have been changes in the health system as lessons are learnt.
- Another limitation is that the estimation of the recovery period may be difficult in settings where the pandemic is yet to be brought under control. This part of the analysis may be subject to delays until an appropriate period is determined.

For peer review only

INTRODUCTION

The novel COVID-19 disease, which was first reported in December 2019 and rapidly spread globally, has led to an unprecedented increase in demand on health systems to care for people infected, necessitating the allocation of significant resources, especially medical resources, towards the response. The increased demand, compounded by the restrictions on movement instituted as part of containment measures may have led to disruptions in the provision of essential services, including sexual and reproductive health (SRH) services [1,2].

The novel coronavirus is new to humans and only limited scientific evidence is available on the impact of COVID-19 on SRH service delivery. However, lessons from the Ebola and Zika virus outbreaks have highlighted the severe disruptions in SRH services that expose women and girls in particular to preventable health risks [2,3]. Some services may be unavailable due to either facilities and health workers being repurposed to care for COVID-19 patients, patient safety concerns, movement restrictions disrupting travel to health facilities, supply chain disruptions or a reduction in health workers because of increasing numbers being themselves infected by COVID-19 [4,5]. In addition, overwhelmed with COVID-19 cases, clinical staff may not have the time or personal protective equipment (PPE) needed to provide family planning counselling and commodities [5]. Recent evidence suggests disruptions lasting 3-6 months in 2020 left between 4-23 million women in low- and middle-income countries unable to access modern contraceptives, a projected 1.4 million (500,000-2.7 million) unintended pregnancies and an additional 31 million cases of secual and gender-based violence (SGBV) [1, 6-9]. Furthermore, studies have modelled the potential impact, showing that even a 10% reduction in essential SRH services could lead to an estimated 15 million unintended pregnancies, 3.3 million unsafe abortions and 29, 000 additional maternal deaths during the next 12 months [7-10]. Continuity of essential health services while keeping people safe during the response to disease outbreaks such as the COVID-19 pandemic is therefore essential for the prevention of both direct and indirect mortality [4,5].

This is a generic standardized protocol designed to maximize the likelihood that data are systematically collected and shared rapidly in a format that can be easily reproducible, aggregated, tabulated and analyzed across many different settings globally and be useful as templates for use in health emergencies in the future. This will facilitate the comparison of results across regions and countries and will potentially improve the quality of observational studies by identifying and minimizing biases. Given that use in different settings will require some adaptation, these possibilities have been highlighted throughout the protocol.

The introduction should be updated with country specific data on COVID-19 epidemiology and current research findings prior to submission to local/country national institutional review boards

For peer review only - http://bmjopen.bmj.com/site/about/guidelines.xhtml

The main aim of this study is to assess the availability of contraception, comprehensive abortion care, STI prevention and treatment (including HIV) and SGBV care and support services in local health facilities during COVID-19 pandemic. The four specific objectives of the study are:

1. To explore the availability of, and health facility readiness to provide these services in areas most affected by COVID-19.
2. To assess the availability and quality of services and barriers to the utilization of these services from clients' and providers' perspectives in the selected COVID-19 affected areas.
3. To assess the post-pandemic recovery* of the facilities in the provision of these services in comparison to the pandemic period.
4. To enhance the SRH service capacity in COVID-19 through advocacy, policy briefs, media dissemination and academic papers towards national and regional stakeholders including policymakers, academia, healthcare providers and the community.

**Post-pandemic recovery refers to the period when ideally health facilities have been able to recover from the disruption in service provision, following reduced transmission levels. Caution should be applied to the term "post-pandemic" as services may be still impacted even with lower levels of transmission. Therefore, the research team should be cognizant of the distinct time periods in their facility assessments.*

METHODS AND ANALYSIS

Study design

This is a repeated cross-sectional study, using both quantitative and qualitative methods. The two data collection points, baseline and end-line, will be 9 to 12-months apart. The aim of having two data collection points is to document and share the local evidence with authorities on the SRH services at baseline, and then at end line to track and demonstrate the changes and improvement in services over time. The World Health Organisation (WHO) situational level assessment of COVID-19 transmission will be used as the basis for determining when to conduct baseline and endpoint data collection [11]:

- Level 0: no known transmission in the preceding 28 days, no restriction on daily activities.
- Level 1: basic measures in place, clusters of cases reported controlled with basic measures, limited and transient disease.
- Level 2: local transmission from imported cases to close contacts. Contact tracing, physical distancing and quarantine measures can contain the spread.
- Level 3: community transmission, where source of infection is untraceable, outbreak rapidly spreads in clusters.
- Level 4: disease outbreak has become an epidemic, where there are major clusters of infection all over the country, high number of deaths and it is very difficult to control transmission without strict containment measures.

Countries should time the baseline and end-line data collection as follows: Baseline data collection is rproposed when countries are experiencing level 3 or 4 transmission (referred to here as peak transmission/pandemic period), as disruptions in service provision are likely to occur during these periods. End-line data collection is proposed when countries have tentatively

For peer review only - http://bmjopen.bmj.com/site/about/guidelines.xhtml

managed to contain the spread and are at levels 0,1 or 2 (referred to here as post-pandemic period/after the pandemic) after experiencing levels 3 or 4.
Given the dynamic nature of the epidemic, where each country is experiencing varying levels of COVID-19 transmission (and the challenges from the resurgence of variants and sudden increase in the number of cases), it is difficult to assume standardized pandemic conditions at country level. Therefore, the time interval between data collection points is just an estimation. For countries that have contained the spread, it may be decided to collect baseline data retrospectively, selecting the peak period. However, strategies to minimize or avoid recall bias should be used.

Study Setting

The study will be conducted in geographical areas selected based on the epidemic status. To get variation in responses, health facilities will be selected as focal points considering their qualification to provide SRH services and other criteria (see section 3.4) for the researchers to access community members for participation in the study. It is expected that variation in health facilities distributed within different geographical areas will provide access to communities of all socioeconomic backgrounds.

Due to various local realities and political factors, countries should select the research study sites based on geographical location, organization of SRH service delivery and epidemic status where COVID-19 is likely to have significantly affected service delivery (given that even within countries the transmission status differs, hence the differential impact on facilities in different geographical areas). Consultation with the Ministry of Health will help identify the areas most affected by COVID-19.

Study Population

The study population will be women seeking SRH services from the selected health facilities and the partners, who will be from different households.This technique will offer a measure of protection for those women who may be at risk just because they participated in the study.

In contexts where it is not customary for men to accompany their partners when they seek SRH services, other options can be used to access them. Healthcare providers can be medical doctors, nurses, midwives, nurse assistants, allied health professionals and other cadres depending on the norms and standards for the provision of SRH services in different country contexts.

Sample selection

Each sample will be selected based on specific criteria, as well as specific country contextual factors.

For peer review only - http://bmjopen.bmj.com/site/about/guidelines.xhtml

Health facility selection

The minimum criteria for selection should include availability of human resources, primarily the qualification to provide contraception, safe abortion, including the treatment of complications and the provision of post-abortion care, STI care and treatment and support for women experiencing SGBV. In addition, the diversity of health facility capacity, administrative rank, urban/rural setting and the willingness of the providers in charge to participate in the study, as well as how the COVID-19 response has been organized in terms of treatment centers. These criteria for selection of health facilities within the geographical sites were made to encourage a representative mix of facilities. The same health facilities will be used for both data collection points to highlight the changes in SRH delivery due to COVID-19 and post-pandemic recovery.

Given the dynamic nature of the pandemic *some flexibility should be given to all study sites in the selection of facilities, maintaining the minimum criteria specified above*

Qualitative sample

Selection of women

Women will be recruited for in-depth interviews (IDIs) and focus group discussions (FGDs). The women will be purposively selected to obtain a sample of women and their partners. Selection will be based on 1) being of reproductive age (18-49) and 2) having sought or tried to receive SRH services from local health facilities. The study will use gatekeepers, who will be healthcare provides not involved in the study to approach the women as they leave the health facility. Their role will be limited to the identification and introduction of potential participants to the study team.

For the IDIs, a sample of 10~15 women (or until saturation) and 6~12 partners (or until saturation) will be recruited. For countries with more enrolled health facilities in the study, the maximum number of the IDIs will be with 10 women (or until saturation) at reproductive age seeking for reproductive healthcare services, and with 5 partners (or until saturation) per health facility. This will be done as an exit interview and the women will be consecutively selected until the desired sample (or saturation) has been achieved.

For FGDs, gatekeepers will also ask participants about their level of comfort discussing experience seeking SRH issues in a group before being recruited. About 6-8 participants per FGD will be recruited, with the expectation of at least two focus groups per facility. Some female and male participants will also be invited for individual interviews after the FGDs.

This is also to note that by interviewing husbands and wives will be from different households; by not interviewing husband-wife from the same household will operationalize the concept of "do no harm" and offer a measure of protection for those women who may be at risk just because they participated in the study.

For peer review only - http://bmjopen.bmj.com/site/about/guidelines.xhtml

The ages can be adjusted to account for differences in the nationally recognised reproductive age group in different contexts. Inclusion of girls younger than 18 years should take into consideration ethical issues of assent and consent.

To adjust to challenges posed by COVID-19 transmission, additional participants can be recruited through online recruitment from chat groups such as Facebook, Twitter or WhatsApp. Careful attention must be paid to the ethical issues of privacy and confidentiality of participants and integrity of the researchers (transparency of aims, details, risks and benefits of the study, obtaining the necessary permission to join restricted groups and the presence of the researcher in the group). Other challenges of online recruitment that need attention include retention of participants, potential selection bias, verification of participant identity and comprehension of informed consent [12]. Care needs to be taken by hiding the names and faces (e.g., via video conference calls) of the online participants, responsible handling of participants' personal information to minimize the likelihood of embarrassment, loss of dignity or harm because of the online recruitment process to address the online privacy and confidentiality concerns. The moderators will ask the participants to give a short self-introduction, using pseudonyms/numbers, instead of their actual names, and not using video options. This includes not disclosing any of this information without the participants consent.

Selection of healthcare providers

Approximately 1 to 2 healthcare providers per health facility will be purposively selected and only those who 1) deliver SRH services, 2) are most knowledgeable about readiness and availability of SRH services 3) are stationed in the SRH clinic and have been working at the clinic for at least 6 months before the pandemic started will be selected for inclusion in the study. This information will be obtained from the facility in-charges.

Quantitative sample

Selection of Women

In each health facility, a maximum of 3 clients from each section (post-abortion care, family planning, SGBV, STI, abortion) will be consecutively selected to achieve up to 20 clients per health facility.

Selection of healthcare providers

One healthcare provider, preferably most knowledgeable in the health facility about SRH services provided, as determined by seniority, position or function, for example, will be selected to assist in the health facility assessment.

Data Collection

FGDs and IDIs will be used to understand client's perspectives on their experiences in accessing SRH services during and after peak transmission and healthcare providers' perspectives of SRH service availability and readiness in COVID-19-affected areas. The health system assessment will

For peer review only - http://bmjopen.bmj.com/site/about/guidelines.xhtml

be used to assess the health system response to COVID-19. Study teams should be aware of the possibility that healthcare provider burnout or heavy workload may affect willingness or ability to participate in the study. Adaptations to data collection should be made to avoid overburdening the healthcare providers and clearly explained during recruitment.

Data Collection tools

There will be five sets of tools for clients- 1 FGD guide for women (published as **supplementary file S1**), 1 FGD guide for partners/men (published as **supplementary file S2**), 1 interview guide for women (published as **supplementary file S3**), 1 interview guide for men (published as **supplementary file S4**) and and 1 interview guide for healthcare providers (published as **supplementary file S5**).

For the facility assessment, a facility and readiness assessment questionnaire has been developed based on the following validated tools [13-16]:

(1) WHO Service Availability and Readiness Assessment (SARA) guide
(2) WHO Health Facility Readiness Checklist
(3) WHO safe abortion assessment tool
(4) SGBV Secual and gender-based violence Quality Assurance Tool

The facility and readiness assessment tool comprises five modules: (1) health services continuation; (2) family planning services; (3) abortion services; (4) STI and (5) SGBV; in line with the study SRH focus areas. The following indicators will be used the assess the availability and readiness:

(1) Policies and plans
(2) Service maintenance and referrals
(3) Infrastructure
(4) Commodities
(5) Human resources

Each study site will adapt (including translation to local language) the tool to its specific context, taking into consideration the existing policies, SRH treatment guidelines, staffing norms and standards, types of facilities, national essential medicines lists and national health information systems among others. After adaptation to specific country context all tools should be piloted before being used.

Data Collection Methods

Participant Informed Consent Process

All study participants will be taken through a detailed informed consent process (for participation and audio-recording), which will be documented and those who agree to participate in the study will be asked for signed written (oral consent for those who cannot write, or for situations that necessitate online data collection) consent.

Given that data collection will be conducted during COVID-19, an assessment of the risks to the study team and participants should be made. The decision of whether to conduct face-to-face

For peer review only - http://bmjopen.bmj.com/site/about/guidelines.xhtml

or remote data collection should be based on the risk assessment, followed by context-specific recommendations on adaptations to the original data collection process. Remote data collection will necessitate adaptation to the length of interviews and discussions, rapport building, oral informed consent, privacy and confidentiality measures as well as making special arrangements where access to internet and mobile connectivity is limited [17]. Face-to-face data collection will require special arrangements to ensure the safety of the study team and participants, following WHO as well as country-specific safety protocols, including educating and training the research team and participants about COVID-19, provision of PPE and hygiene supplies, mandatory hygiene practices and sanitization of venues and equipment and physical distancing [18].

The FGDs and IDIs will be conducted at times and in venues that are considered both convenient and safe for the participants to freely discuss the subject matters. In addition, qualified researchers with experience in conducting IDIs and facilitating FGDs will be trained to ensure the validity of the data collection and will be selected based on the gender of the participants. The FGDs and IDIs will be semi-structured and follow a topic guide specific to each group of participants but will take place as a conversation in which the researchers promote a safe, comfortable environment to enable a comprehensive and candid record. In addition to the semi-structured interview, narrative interview techniques may be used depending on the participants' narratives about life events and reproductive healthcare needs.

The FGDs and IDIs will be audio-recorded. Prior consent for recording will be sought again from each participant. Anonymity of the participants will be ensured by removing any personal or family identifiers and all recorded sessions will be coded for purposes of identification with a date, geographical site and session number. After each session, the recording will be sent to the team supervisor for secure storage for transcription. The audio files will be encrypted and sent to an electronic database which will be shared with WHO. Therefore, only the research team will be authorized to listen to the recordings. These audio recordings will be retained until they have been transcribed and checked for accuracy, after which they will be destroyed.

The FGDs will explore knowledge about COVID-19, care-seeking during COVID-19 and risk perceptions and availability of SRH services (end-line FGD will also explore post-pandemic services in comparison to the pandemic period). Before starting the FGD, the facilitators will collect socio-demographic data for each participant, build rapport with and among participants and set ground rules to ensure positive group dynamics that foster an effective and rich discussion. The facilitators will also use this time to know and understand the emic categories (field research and viewpoints obtained from within the social group, from the perspective of the subject) used by locals to describe their perceptions and practices related to the study topics. Each FGD is expected to last approximately 60 – 90 minutes.

IDIs with women will explore the psychosocial effects of COVID-19 on fertility desires, knowledge of COVID-19, risk perceptions and concerns about effects on SRH, care seeking behaviors, experience and barriers in accessing SRH services reproductive health, particularly comprehensive abortion, STI prevention and treatment and SGBV care and support, and their related needs for accurate information and reproductive health services. IDIs with partners will

For peer review only - http://bmjopen.bmj.com/site/about/guidelines.xhtml

explore their knowledge of COVID-19, risk perceptions and concerns about effects on SRH, their influence on the access to SRH services of their partners and find out what role they play when their partners require SRH services like contraception, comprehensive abortion care, STI prevention and treatment and SGBV care and support. Each interview is expected to last approximately 40 – 60 minutes. IDIs for the women and their partners will be conducted separately.

In some countries, abortion is illegal, posing challenges in data quality since participants, fearing reprisals, may not provide accurate responses to abortion-related questions. Necessary precautions should be taken to ensure the interviews are conducted in a supportive and non-judgmental manner, encouraging the participants to respond freely. These include selection and training of interviewers to enable them to overcome their biases and stereotypes about abortion, building trust and rapport with participants and selection of interview venue to guarantee privacy [19].

Given the sensitive nature of the data being collected, care must be taken in the selection of data collectors to ensure protection of participants. The gender, experience and attitudes of the data collectors are important considerations. Training of data collectors is essential with emphasis on the risks to the participants and how to protect them, the importance of non-judgmental attitudes and provision of necessary support to participants.

Interviews with healthcare providers will be based on WHO's six building blocks framework [20 with a focus on the delivery of the focus SRH services during COVID-19; their perceptions on the roles and responsibilities of different parties to provide these services in the COVID-19 context; health system capacity to provide good quality of care for people during COVID-19; training needs, attitudes, biases about contraception, abortion, STI and SGBV in the context of COVID-19; and perceived psychosocial effects on men and women, their families and local communities.

The health availability and readiness assessment will be implemented in all the selected facilities using a cross-sectional survey design to highlight gaps or service delivery issues during COVID-19 and recovery in service availability and readiness in the same health facilities. The assessment will also include a review of health facility records in family planning and contraception, STI care and treatment and care and support for women who have experienced SGBV to assess the availability, type and range of commodities offered. A follow-up assessment will be done at end-line. The follow-up survey plans to assess the recovery in service availability and readiness in the same health facilities. The research team will fill out the data extraction tool and the questionnaire with the assistance of the most knowledgeable person in the health facility about SRH services provided (senior health care provider and/or administrator) for each section of the questionnaire. The assessment will also include a questionnaire to the clients of the health facility.

The client questionnaire contains questions related to general characteristics of clients and their experience in seeking SRH services. All the selected health facilities will be required to collect this

For peer review only - http://bmjopen.bmj.com/site/about/guidelines.xhtml

data from all clients seeking SRH services once every month throughout the study period, to capture trends in service availability and utilization.

Data Analysis

Health facility assessment data will use the presence of the tracer items for the provision of these services, such as availability of guidelines, staff and essential commodities. Data will be entered into an electronic database using data entry programmes such as Epi data and CSPro. Study sites using electronic data collection may skip this step and for WHO supported centres implementing the study an online platform (OpenClinica) will be used. Descriptive analysis will be used to illustrate the basic characteristics of the different facilities, including the monthly number of clients, types of procedures provided, number of medical staff, stocks of drugs, etc.

Qualitative analysis will form the main analysis for this study, beginning while data collection is ongoing to assess progress and determine any problems. The audio-recorded data will be transcribed verbatim and de-identified by using ID numbers in place of names. Where required, transcriptions will be translated to English and back-translated and analyzed using content analysis, according to the suggested steps by Elo & Kyngäs (2008) [21], as illustrated in Table 1. The WHO team based in Geneva, in conjunction with the study principal investigator (PI) and project team, will support and monitor the data analysis.

Table 1. Planned analytical procedures for content analysis

Phase	Procedures
Preparation	Reading through verbatim transcriptions of the interviews several times to familiarize with the data, gain an understanding of what has been expressed, selecting the unit of analysis, deciding on the analysis of manifest content.
Organizing	Open coding and creating categories, grouping codes under higher order headings, formulating a general description of the research topic through generating categories and subcategories as abstracting.
Reporting	Reporting the analysis process and the results through models, conceptual systems, conceptual map or categories, and a story line.

Data management and access

Data management plans should include information about how data will be stored, including levels of protection, who will have access to the data and when it would be destroyed, how data will be transferred (if needed) securely. In countries where data protection legislation exists, protocols should specify that data would be handled in accordance with those policies.

Quantitative data

For peer review only - http://bmjopen.bmj.com/site/about/guidelines.xhtml

Data collectors will be trained on data collection, transmission, verification, storage and primary analysis to assess errors. Data can be collected electronically, or on paper.

Qualitative data

The quality and trustworthiness of the qualitative data collected will be assured through triangulation, as we will conduct interviews with different parties, including health professionals, health workers, and clients. The process of the study will be clearly documented to ensure the transparency and the rigor of the study.

Ethics and dissemination

Scientific approval has been obtained from WHO research project review panel (RP2). Ethical approval for this study was also obtained from the WHO Ethics Review Committee (protocol ID CERC.0103). Each study site is required to obtain the necessary ethical and regulatory approvals that are required in the corresponding country. Ethical considerations of informed consent, voluntary participation, privacy and confidentiality, anonymity and compensation for incidental costs (the decision to offer compensation and its value will depend on the specific country context and respective local policies) will be respected and detailed in the informed consent process.

Given the sensitive nature of the data being collected, special care will be taken to ensure the “do no harm” principle is respected. This will include making arrangements with locally available support services (medical, psychosocial, legal) where participants can be referred, training the research team to respond to and provide immediate emotional support to study participants or for situations where support services are not locally available or are inadequate [22, 23].

At country level the results will be presented to policy makers, researchers, managers through policy briefs and workshops in collaboration with the WHO office. The results will also be disseminated among the communities/participants through online platforms/text messages, which will be provided to the participants by the data collectors/health workers. In addition, the results will also be presented to the scientific and funding community in collaboration with WHO country and regional offices, by communication and manuscript publications in peer-reviewed international journals

Discussion

This is a generic standardized protocol designed to be used across many different settings globally and to be useful as templates for use in health emergencies in the future. This will facilitate the comparison of results across regions and countries.

This study provides a unique opportunity to assess the availability, utilization, and readiness of the SRH services during the COVID-19 pandemic. The main lesson learned will be the adaptations of the health system in pandemic situations and what can be done to ensure continuity of essential SRH services.

For peer review only - http://bmjopen.bmj.com/site/about/guidelines.xhtml

The mixed methods approach and panel design with two data collection points enable a comprehensive analysis of the barriers to availability, utilization and readiness of sexual and reproductive health services during COVID-19, as well as the post-pandemic recovery as transmission is contained.

This study is well placed to advocate for the development and strengthening of policies and services that are responsive to the needs of women and girls during health emergencies, given the potential to exacerbate existing gender and social inequalities and increase the vulnerability of women and girls to preventable health risks. The use of a mainly qualitative approach places women's rights and needs at the center of the debate. The inclusion of partners will enhance understanding of gender dynamics and support efforts towards identifying strategies to enhance positive male involvement and engagement in women's sexual reproductive health and rights.

Contributor ship statement

Contributors: MA and CK conceptualized and designed the protocol. All authors contributed to developing the detailed methodology. SK, EC and MA drafted the first draft manuscript. SJ, BL, CJG, LP, ME, BV, KH, and SA edited the draft, provided critical inputs, and approved the final written version for publication.

Funding Statement

This research has been supported by German Federal Ministry of Health (BMG) COVID-19 Research and development funding to WHO. It has also been supported by the UNDP-UNICEF-WHO-World Bank Special Programme of Research, Development and Research Training in Human Reproduction (HRP); a co-sponsored programme executed by the World Health Organisation (WHO). The adaptation and implementation of this protocol in the HRP collaborating centres in Brazil, Burkina Faso, China, Ghana, Italy, Kenya, Pakistan, Thailand and the United Kingdom was also funded by the same. The views of the funding body have not influenced the content of this manuscript. This article represents the views of the named authors only and does not represent the view of their affiliated institutions.

Disclaimer

This report contains the collective views of an international group of experts and does not necessarily represent the decisions or the stated policy of the World Health Organization.

Data Sharing Statement

Data sharing not applicable as no datasets generated and/or analysed for this protocol.

Competing interests

The authors declare that they have no competing interests.

Patient and public involvement

No patient or public involvement.

For peer review only - http://bmjopen.bmj.com/site/about/guidelines.xhtml

Acronyms

COVID-19	Corona Virus Disease 2019
CSPro	Census and Survey Processing System
FGD	Focus Group discussion
SGBV	Gender Based Violence
IDI	In-depth Interview
RP2	Project Review Panel
SRH	Sexual and Reproductive Health
STI	Sexually Transmitted Infections
WHO	World Health Organisation

References

1. Riley T, Sully E, Ahmed Z, Biddlecom A. Estimates of the potential impact of the COVID-19 pandemic on sexual and reproductive health in low- and middle-income countries. *Int Perspect Sex Reprod Health* 2020; 46:73–76. doi: 10.1363/46e9020.
2. Addressing human rights as key to the COVID-19 response. Geneva: World Health Organization; April 2020 (WHO/2019-nCoV/SRH/Rights/2020.1; https://www.who.int/publications/i/item/addressing-human-rights-as-key-to-the-covid-19-response (accessed August 15, 2020)
3. Sexual and Reproductive Health and Rights during the COVID-19 pandemic: A joint report by EPF & IPPF EN. April 2020. https://www.ippfen.org/sites/ippfen/files/2020-04/Sexual and Reproductive Health during the COVID-19 pandemic.pdf (accessed August 16, 2020)
4. COVID-19: operational guidance for maintaining essential health services during an outbreak: interim guidance. 25 March 2020. Geneva: World Health Organization. 2020 WHO/2019-nCoV/essential_health_services/2020.1; https://apps.who.int/iris/handle/10665/331561 (accessed on August 15, 2020).
5. Maintaining essential health services: operational guidance for the COVID-19 context. World Health Organisation; 2020. https://apps.who.int/iris/handle/10665/332240 (Accessed August 15, 2020)
6. Impact of the COVID-19 pandemic on family planning and ending secual and gender-based violence, female genital mutilation and child marriage: pandemic threatens achievement of the transformative results committed to by UNFPA. New York, United Nations Population Fund; 2020 https://www.unfpa.org/resources/impact-covid-19-pandemic-family-planning-and-endinggender-based-violence-female-genital-mutilation (accessed on August 15, 2020).
7. Millions more cases of violence, child marriage, female genital mutilation, unintended pregnancy expected due to the COVID-19 pandemic [Internet]. Unfpa.org. 2020 [cited 3 May 2020]. Available from: https://www.unfpa.org/news/millions-more-cases-violence-child-marriage-female-genital-mutilation-unintended-pregnancies#
8. Coronavirus Disease (COVID-19) Pandemic UNFPA Response Plan. June 2020 https://www.unfpa.org/sites/default/files/resource-pdf/UNFPA_Global_Response_Plan_Revised_June_2020_.pdf (accessed August 16, 2020)

For peer review only - http://bmjopen.bmj.com/site/about/guidelines.xhtml

9. United Nations Population Fund. Impact of COVID-19 on family planning: What we know one year into the pandemic. Technical note. New York, UNFPA;2021
10. Julia Hussein. COVID-19: What implications for sexual and reproductive health and right globally? *Sex Reprod Health Matters* 2020;28(1):1746065 DOI:10.1080/26410397.20201756065.
11. World Health Organization. Considerations for implementing and adjusting public health and social measures in the context of COVID-19. Interim guidance. Geneva, World Health Organization;2020 https://www.who.int/publications/i/item/considerations-in-adjusting-public-health-and-social-measures-in-the-context-of-covid-19-interim-guidance (Accessed December 5, 2020)
12. Gelinas L, Pierce R, Winkler S, et.al. Using social media as a research recruitment tool: ethical issues and recommendations. *Am J Bioeth 2017*;17(3):3-14
13. World Health Organization. Service availability and readiness assessment (SARA): implementation guide. Geneva: World Health Organization; 2015 http://www.who.int/healthinfo/systems/sara_implementation_guide/en/. (Accessed August 20, 2020)
14. World Health Organization. Safe abortion: technical and policy guidance for health systems. 2nd ed. Geneva: World Health Organization; 2012 http://www.who.int/reproductivehealth/publications/unsafe_abortion/9789241548434/en/. (Accessed March 10, 2021)
15. JHPIEGO. 2018. Secual and gender-based violence Quality Assurance Tool: Standards for the Provision of High-Quality Post-Violence Care in Health Facilities. https://www.who.int/reproductivehealth/publications/post-violence-care-in-health-facilities/en/ (Accessed March 10, 2021)
16. World Health Organisation. Rapid Hospital Readiness Checklist: Harmonized Health Service Capacity Assessment in the Context of the COVID-19 Pandemic. Interim Guidance. Geneva: World Health Organization; 2020 https://apps.who.int/iris/handle/10665/332779
17. Hensen B, Mackworth-Young CRS, Simwinga M, et al. Remote data collection for public health research in the covid-19 era: ethical implications, challenges and opportunities. *Health Policy Plan* 2021;czaa158 https://doi.org/10.1093/heapol/czaa158
18. IMPACT. 2020. SOPs for Data Collection during COVID-19. https://www.reachresourcecentre.info/wp-content/uploads/2020/05/DataCollectionSOPCOVID-19.pdf (Accessed March 10, 2021)
19. Zulu JM, Ali J, Hallex K, et.al. Ethical challenges in research on post-abortion care with adolescents: experiences of researchers in Zambia. *Glob Bioeth* 2018; 31(1):104-119
20. World Health Organisation. Everybody business: strengthening health systems to improve health outcomes: WHO's framework for action. Geneva, World Health Organisation; 2007. https://www.who.int/healthsystems/strategy/everybodys_business.pdf?ua=1 (Accessed August 15, 2020)
21. Elo S, Kyngäs H. The qualitative content analysis process. *J Adv Nurse* 2008;62:107-115.
22. UN Women, World Health Organisation. Violence Against Women and Girls. Data Collection during COVID-19. New York, UN Women; 2020 https://www.unwomen.org/-

For peer review only - http://bmjopen.bmj.com/site/about/guidelines.xhtml

/media/headquarters/attachments/sections/library/publications/2020/vawg-data-collection-during-covid-19 (Accessed March 10, 2021)

23. World Health Organisation. Putting Women First: Ethical and Safety Recommendations for research on Domestic Violence Against Women. Geneva, World Health Organization;2001 https://apps.who.int/iris/bitstream/handle/10665/65893/WHO_FCH_GWH_01.1.pdf?sequence=1 (Accessed Jan 6, 2021)

For peer review only

Focused group topic guide for women

Demographic information (Note: Collect data on individuals before the FGD)

- Age (in completed years)
- Education (highest level attained)
- Residence (actual, urban, rural, slum)
- COVID-19 infection history (self, close family, work mates, friends)
- Social economic status - Employment status (i.e. formal or informal employment), income earning status and possibly income ranges as suitable in the country setting.

Knowledge about COVID-19 (such as cause, signs and symptoms)

1. What is COVID-19? What do you know about COVID-19?
   PROBE: for causes, transmission, signs, symptoms, sources of information about COVID-19
2. Did you perceive yourself to be at risk for COVID-19 infection?
   PROMPT: Probe for any special considerations (pregnancy, pre-existing health conditions, others).

Care seeking and perception of risk about being infected by COVID-19

3. The decision-making process of treatment seeking during COVID-19
   PROBE: Ask who influenced the decision to seek care during COVID-19 (self, partner, other family member, etc?)
4. Perception of risk of infection during SRH care seeking.
   PROMPT: How did this affect health seeking behaviour during COVID-19?
5. The treatment-seeking process during COVID-19
   a. Were your needs for accurate information on reproductive health services fulfilled? How? Or Why not? PROBE: information sources: brochures, social media, billboards, other printed media, information on service hours or location where services are offered etc.
   b. What were your needs for reproductive health services? Were these needs fulfilled? **PROBE**: Contraception? Abortion care? STI?
   c. Did you encounter any barriers in your bid to access needed reproductive health services? PROBES: transportation, behaviour of other persons, etc.

   PROMPT: Interviewer should probe for other context specific intermediaries to seeking care. E.g. paperwork for insurance
6. Do you think that reproductive services were readily available to clients who needed them? Specifically, the availability of the reproductive health medicine and medical supplies needed by clients based on the following:
   a. Availability of staff, medicines and supplies for SRH during COVID-19
   b. Schedule of treatments/appointments during COVID-19
      PROMPT: Probe for changes in the delivery of SRH services (specifically for family planning, abortion, post-abortion care and STI care) i.e. appointment-based system introduced.
   c. Interaction between the service provider and the patient was during COVID-19. PROBE: Interactions with different cadres of health workers. Nurses? Midwives? Doctors? Records? OPD staff? Etc.
   d. Was the interaction between service providers and staff described above different from pre-COVID-19 time? How?
7. What were some of the safety measures ensured by the health facility to protect clients from COVID-19 during services
8. Do you think that the COVID-19 had any impact on client protection and privacy during services?
   PROBE: What was done (or could have been) differently?

Post-pandemic recovery of the facilities to provide services in comparison to the pandemic period.

GENERAL COMMENTS: Based on the country situation, the probes for the questions above can be extended to cover the post-pandemic period if applicable.

For peer review only

Focused group topic guide for men

Demographic information

- Age (in completed years)
- Education (highest level attained)
- Residence (actual, urban, rural, slum)
- COVID-19 infection history (self, close family, work mates, friends)
- Social economic status: Employment status (i.e. formal or informal employment), income earning status and possibly income ranges as suitable in the country setting.

Knowledge about COVID-19 (such as cause, signs and symptoms)

1. What is COVID-19? What do you know about COVID-19?
   PROBE: for causes, transmission, signs, symptoms, sources of information about COVID-19
2. Did you perceive yourself to be at risk for COVID-19 infection?
   PROMPT: Probe for any special considerations (pregnancy, pre-existing health conditions, others).

Care seeking and perception of risk about being infected by COVID-19

1. What role did you play in the decision-making process of treatment seeking during COVID-19?
   PROBE: final decision maker (man only, joint, woman only, other); stage of involvement (decision stage or care seeking stage).
2. The role of men in the treatment-seeking process during COVID-19
   a. What role did you play in seeking for accurate information on reproductive health services? Were your needs fulfilled? How? Or Why not?
      PROBE: information sources: brochures, social media, billboards, other printed media, etc. The role of men in looking for sexual reproductive health services (including contraception and abortion care) and whether the needs were fulfilled
   b. What role did you play in looking for reproductive health services? Were your needs for reproductive health services fulfilled?
      PROBE: Contraception? Abortion care? STI?
   c. Did you encounter any barriers in your bid to access needed reproductive health services? PROBES: transportation, behaviour of other persons, entry restrictions for men, etc.
      NOTE: Interviewer should probe for other context specific intermediaries to seeking care. E.g. paperwork for insurance
3. Do you think that reproductive services were readily available to clients who needed them? The availability of the reproductive health medicine and medical supplies needed by clients?
   a. The schedule of treatments/appointments during COVID-19?
4. How was the interaction between the service provider and you as the male partner during COVID-19?
   PROBE: Interactions with different cadres of health workers. Nurses? Midwives? Doctors? Records? OPD staff? Etc
5. Was the interaction between service providers and staff described above different from pre-COVID-19 time? How?

6. What were some of the safety measures ensured by the health facility to protect clients from COVID-19 during services?
   PROBE: Were male partners stopped from entering consulting and treatment rooms due to COVID-19?
7. Do you think that the COVID-19 had any impact on client protection and privacy during services?
8. **PROBE**: What was done (or could have been) differently? E.g. barriers in seeking SRH services during COVID

Post-pandemic recovery of the facilities to provide services in comparison to the pandemic period.

COMMENT: Based on the country situation, the probes for the questions above can be extended to cover the post-pandemic period if applicable.

For peer review only

In-depth Interview topic guide for women

1. Demographic information
    - Age
    - Ethnicity
    - Marital status
    - Socio-economic status
    - Occupation
    - Education status
    - Religion
    - Place of residence and housing condition
    - Obstetric characteristics (no. of pregnancies, parity)

2. Knowledge about COVID-19
    - Cause (sexual transmission)
        - How do you think COVID-19 virus is transmitted?
        - Do you think it can be transmitted sexually?
    - Signs and symptoms of COVID-19
        - What are the sign and symptoms of COVID-19?
        - If yes, can you mention some that you know?
    - Care-seeking for COVID-19 infection
        - Do you know what to do/who to contact/ where to go if you experience COVID-19 signs and systems?
        - If yes, can you explain?
    - Sources of information and trusted communication channels (v. rumors, misinformation) Experience of COVID-19 (direct / indirect)
        - Where did you get information about COVID-19?
        - Was the information you obtained accurate?
        - Can you give me an example of accurate information you obtained?
        - Were there any rumors and/or misinformation about COVID-19?
        - How did you know that this information was just rumors or misinformation?
        - Have you fund sources of information that you can trust?
        - How did you know which information sources to trust?
        - Have you been infected by COVID-19?
        - Do you know of anyone who has been infected? If yes, was it a close friend or family member?
    - Perception of risk about being infected by COVID-19
        - Do you think you are at risk of being infected by the COVID-19 virus?
        - If no, can you tell me why not?
        - Do you think you can be infected whilst seeking care?
    - Ways to prevent being infected by COVID-19 (probe at individual, household, community levels)
        - Do you know how to prevent infection from COVID-19?
        - What can individuals do? What can households do? What can communities do?
    - Responsibility to prevent COVID-19 (probe at individual, household, community levels, authorities)
        - Who is responsible for the prevention of COVID-19 infection? Do you think this

For peer review only

responsibility was fulfilled by individuals, households, communities and authorities? How?

- Religious / socio-cultural practices and influencers
  - Are there any religious / socio-cultural practices that are used to prevent COVID-19 infection?
  - Does participation in any religious / socio-cultural practices make you practice prevention of COVID-19 differently from what is promoted by health professionals?

3. Sexual and Reproductive Health
   - Decision-making and agency to act
     - Who makes the decisions related to your sexual and reproductive health needs (self, husband/partner or family member)?
     - Who is usually responsible for carrying out the decision?
   - Issues of access / utilization
     - Did you need to seek SRH services during COVID-19?
     - If yes, were you able to access the services? How did you access the services?
     - If no, what were the barriers? What did you do?
   - Challenges faced
     - Are there any other challenges you faced in seeking SRH services during COVID-19? What are they and how did you address/manage the challenges?

4. Contraceptive practices
   - Changes in practice (if any) due to COVID-19
     - Which method of contraception do you normally use?
     - Did you have to change your normal method of contraception during COVID-19? If yes, which method and what influenced the change?
     - If no, why didn't you change from your normal practice?
   - Is pregnancy being avoided / delayed due to COVID-19? If yes, why?
   - Access to / utilization of different methods of contraception (barriers / drivers)
   - Which methods of contraception were accessible to you during COVID-19?
   - Was there a difference in the methods of contraception accessible to you compared to before COVID-19?
   - What was causing the difference?
   - Challenges in access / utilization
   - Are there any other challenges you faced in accessing/utilizing contraception services during COVID-19?
   - If yes, what were these challenges and how did you cope with them?

5. Induced abortion
   - Perceptions about abortion at normal times
   - The role abortion plays in women's lives
     - Why do you think women seek abortion services?
     - Do you think abortion is an essential service for women? If yes, in what way?
   - Fear of stigma
     - How does family, community and society view women who seek or have sought abortion services?

For peer review only

  - Does this stigma affect your decision whether to have an abortion?
  - Does this stigma affect how you will seek abortion services? If yes, how?
  - What do you think are the effects of this stigma on women who need abortion services?
- Inequities in access to safe abortion
  - Do you think safe abortion services are equally accessible to all women who need them?
- Perceptions about abortion during COVID-19
  - Would you choose to seek the abortion services during COVID-19, if you need one?
  - If no, why not? If yes, where and how would you access the service?

- Access to different methods of abortion (barriers / drivers)
  - Did you seek abortion services during COVID-19? If yes, which abortion methods were available for you (medical abortion or surgical abortion)?
  - What pushed you to have an abortion? If you were given a choice of methods, which one did you choose and why?
  - Did you face any barriers in seeking and accessing abortion services during COVID-19?
- Challenges in accessing / utilizing safe abortion services (inequities in access)
  - Are there any other challenges you faced in accessing/utilizing safe abortion services? If yes, what were the challenges and how did you address them?

- 9. Violence against women (*Interviewer should use the term that is used locally to describe VAW)*

- Formal and informal systems of support, and coping strategies for VAW
  - Have you or any woman you know experienced any form of VAW?
  - What are some of the systems of support available for women who have experienced VAW?
  - Do you know where and how to access this support?
  - What are some of the coping strategies women who have experience such violence use?
- Perceptions of safety in home and in community safety/increased risk during COVID-19
  - Do you think are safer or at increased risk of experiencing VAW at home or in the community during COVID-19?
  - What has made it safer/riskier for women during COVID-19?
- Women's expectations of services for gender-based violence
  - If you or any other woman experiences GBV during COVID-19, do you expect the care and support services to be available?
  - Do you know where and how to access these services?
  - Do you expect any barriers to accessing these services during COVID-19? If yes, what kind of barriers?
- What are additional stress factors (e.g. school-age children at home, older parents, loss of job/income, partner loss of job, etc.) that increase the likelihood of VAW?
- How does stress affect frequency and severity of violence, particularly during COVID-19?

For peer review only

6. Sexually transmitted infections *(interviewer should use the locally known terms for STIs and the specific diseases)*
   - Perceptions about STI
     - Is anybody at risk of getting a STI? Why or why not?
     - How are people who are known to have a STI perceived in the community?
   - Experience of STI during COVID-19
     - Have you required care and treatment for a STI during COVID-19? If yes, where and how did you seek this service?
     - Were there any barriers to accessing this service? If yes, which ones and what did you do?
     - If no, would you seek care and treatment if required during COVID-19?
     - If no, why not? If yes, where and how would you access this service?

7. Psycho-social
   - Psychosocial issues associated with being infected with COVID-19 (probe: more significant for women/men, more significant for pregnant women)
     - Did you experience any stress, anxiety or depression during COVID-19? If yes, at what level (mild, moderate, severe)?
     - What do you think led to the psychosocial issues you experienced? Was it due to being infected or other related issues or both?
     - Did your partner also experience any psychosocial issues during COVID-19?
     - Who was most affected, yourself or your partner?

8. Final questions
   - On-going concerns about COVID-19 and its possible impact
     - Are there any other concerns about COVID-19 and its impact on you, your family or in general that you wish to express?
   - Needs related to accurate information (probe further information needs – NB consider how to provide information if necessary)
     - Was accurate information readily available and accessible during COVID-19?
     - Was it easy to understand?
     - Was the information that was available adequate?
     - Was there additional information you would have liked to obtain but was missing?
     - Are there better ways of making accurate information accessible to you and others?
   - Needs related to sexual reproductive health services (probe further SRH needs – NB consider how to support/provide if necessary)
   - Do you think SRH services were responsive to your needs during COVID-19?
   - If no, which of your needs were not met?
   - Which SRH services are most important for you, that should be available even in health emergencies like COVID-19?
   - How do you think provision of SRH services can be supported during other health emergencies like COVID-19?

For peer review only

Additional themes will be added as appropriate for each target group and questions revised accordingly (with attention to first person / third person views), and across the three phases of the study.

For peer review only

In-depth Interview topic guide for men

1. Demographic information
    - Age
    - Ethnicity
    - Marital status
    - Socio-economic status
    - Occupation
    - Education status
    - Religion
    - Place of residence and housing condition

2. Knowledge about COVID-19
    - Cause (sexual transmission)
        - How do you think COVID-19 virus is transmitted?
        - Do you think it can be transmitted sexually?
    - Signs and symptoms of COVID-19
        - What are the sign and symptoms of COVID-19?
        - If yes, can you mention some that you know?
    - Care-seeking for COVID-19 infection
        - Do you know what to do/who to contact/ where to go if you experience COVID-19 signs and systems?
        - If yes, can you explain?
    - Sources of information and trusted communication channels (v. rumors, misinformation) Experience of COVID-19 (direct / indirect)
        - Where did you get information about COVID-19?
        - Was the information you obtained accurate?
        - Can you give me an example of accurate information you obtained?
        - Were there any rumors and/or misinformation about COVID-19?
        - How did you know that this information was just rumors or misinformation?
        - Have you fund sources of information that you can trust?
        - How did you know which information sources to trust?
        - Have you been infected by COVID-19?
        - Do you know of anyone who has been infected? If yes, was it a close friend or family member?
    - Perception of risk about being infected by COVID-19
        - Do you think you are at risk of being infected by the COVID-19 virus?
        - If no, can you tell me why not?
        - Do you think you can be infected whilst seeking care?
    - Ways to prevent being infected by COVID-19 (probe at individual, household, community levels)
        - Do you know how to prevent infection from COVID-19?
        - What can individuals do? What can households do? What can communities do?
    - Responsibility to prevent COVID-19 (probe at individual, household, community levels, authorities)
        - Who is responsible for the prevention of COVID-19 infection? Do you think this responsibility was fulfilled by individuals, households, communities and

For peer review only

authorities? How?

- Religious / socio-cultural practices and influencers
  - Are there any religious / socio-cultural practices that are used to prevent COVID-19 infection?
  - Does participation in any religious / socio-cultural practices make you practice prevention of COVID-19 differently from what is promoted by health professionals?

3. Sexual and Reproductive Health
   - Decision-making and agency to act
     - Who makes the decisions related to the sexual and reproductive health needs of your partner (you, partner or family member)?
     - Who is usually responsible for carrying out the decision?
   - Issues of access / utilization
     - Did your partner need to seek SRH services during COVID-19?
     - If yes, was your partner able to access the services? How did your partner access the services?
     - If no, what were the barriers? What did you and/or your partner do?
     - What role did you play in ensuring your partner sought and/or accessed the needed SRH services?
   - Challenges faced
     - Are there any other challenges your partner faced in seeking SRH services during COVID-19? What are they and how did you and/or your partner address/manage the challenges?

4. Contraceptive practices
   - Changes in practice (if any) due to COVID-19
     - Which method of contraception do you and/or your partner normally use?
     - Did you have to change your normal method of contraception during COVID-19? If yes, which method and what influenced the change?
     - If no, why didn't you change from your normal practice?
   - Did your partner avoid / delay pregnancy due to COVID-19? If yes, why?
   - Access to / utilization of different methods of contraception (barriers / drivers)
   - Which methods of contraception were accessible to you and your partner during COVID-19?
   - Was there a difference in the methods of contraception accessible to you compared to before COVID-19?
   - What was causing the difference?
   - Challenges in access / utilization
   - Are there any other challenges you and your partner faced in accessing/utilizing contraception services during COVID-19?
   - If yes, what were these challenges and how did you cope with them?
   - What role did you play in ensuring your partner sought and/or accessed contraception services?

5. Induced abortion

For peer review only

- Perceptions about abortion at normal times
- The role abortion plays in women's lives
  - Why do you think women seek abortion services?
  - Do you think abortion is an essential service for women? If yes, in what way?
- Fear of stigma
  - How does family, community and society view women who seek or have sought abortion services?
  - Does this stigma affect your decision whether to have an abortion?
  - Does this stigma affect how you will seek abortion services? If yes, how?
  - What do you think are the effects of this stigma on women who need abortion services?
- Inequities in access to safe abortion
  - Do you think safe abortion services are equally accessible to all women who need them?

- Perceptions about abortion during COVID-19
  - Would you choose to seek the abortion services during COVID-19, if your partner needed one?
  - If no, why not? If yes, where and how would you access the service?
- Access to different methods of abortion (barriers / drivers)
  - Did your partner seek abortion services during COVID-19? If yes, which abortion methods were available (medical abortion or surgical abortion)?
  - What pushed you and your partner to have an abortion? If you were given a choice of methods, which one did you choose and why?
  - Were there any barriers in seeking and accessing abortion services during COVID-19?
- Challenges in accessing / utilizing safe abortion services (inequities in access)
  - Are there any other challenges you faced in accessing/utilizing safe abortion services? If yes, what were the challenges and how did you address them?
- What was your role in ensuring your partner accessed the abortion service?

6. Violence against women (*Interviewer should use the term that is used locally to describe VAW)*
   - Formal and informal systems of support, and coping strategies for VAW
     - Has your partner or any woman you know experienced any form of VAW?
     - What are some of the systems of support available for women who have experienced VAW?
     - Do you know where and how to access this support?
     - What are some of the coping strategies that women who have experienced such violence use?
   - Perceptions of safety in home and in community safety/increased risk during COVID-19
     - Do you think women are safer or at increased risk of experiencing VAW at home or in the community during COVID-19?
     - What has made it safer/riskier for women during COVID-19?
   - Women's expectations of services for gender-based violence

For peer review only

  - If your partner experiences GBV during COVID-19, do you expect the care and support services to be available?
  - Do you know where and how to access these services?
  - Do you expect any barriers to accessing these services during COVID-19? If yes, what kind of barriers?
  - What do you perceive as your role in ensuring your partner get the care and support needed?
- What are additional stress factors (e.g. school-age children at home, older parents, loss of job/income, partner loss of job, etc.) that increase the likelihood of VAW?
- How does stress affect frequency and severity of violence, particularly during COVID-19?

6. Sexually transmitted infections *(interviewer should use the locally known terms for STIs and the specific diseases)*

- Perceptions about STI
  - Is anybody at risk of getting a STI? Why or why not?
  - How are people who are known to have a STI perceived in the community?
- Experience of STI during COVID-19
  - Has your partner required care and treatment for a STI during COVID-19? If yes, where and how did you seek this service?
  - Were there any barriers to accessing this service? If yes, which ones and what did you do?
  - If no, would you seek care and treatment if required during COVID-19?
  - If no, why not? If yes, where and how would you access this service?
  - What was your role in ensuring your partner accessed the care and treatment?

7. Psycho-social

- Psychosocial issues associated with being infected with COVID-19 (probe: more significant for women/men, more significant for pregnant women)
  - Did you experience any stress, anxiety or depression during COVID-19? If yes, at what level (mild, moderate, severe)?
  - What do you think led to the psychosocial issues you experienced? Was it due to being infected or other related issues or both?
  - Did your partner also experience any psychosocial issues during COVID-19?
  - Who was most affected, yourself or your partner?

8. Final questions

- On-going concerns about COVID-19 and its possible impact
  - Are there any other concerns about COVID-19 and its impact on you, your family or in general that you wish to express?
- Needs related to accurate information (probe further information needs – NB consider how to provide information if necessary)
  - Was accurate information readily available and accessible during COVID-19?
  - Was it easy to understand?
  - Was the information that was available adequate?
  - Was there additional information you would have liked to obtain but was

For peer review only

missing?

- Are there better ways of making accurate information accessible to you and others?

- Needs related to sexual reproductive health services (probe further SRH needs – NB consider how to support/provide if necessary)
- Do you think SRH services were responsive to your needs during COVID-19?
- If no, which of your needs were not met?
- Which SRH services are most important for you, that should be available even in health emergencies like COVID-19?
- How do you think provision of SRH services can be supported during other health emergencies like COVID-19?

Additional themes will be added as appropriate for each target group and questions revised accordingly (with attention to first person / third person views), and across the three phases of the study.

For peer review only

In-depth Interview topic guide for SRH healthcare providers

Demographic information

- Age
- Ethnicity
- Marital status
- Socio-economic status
- Occupation
- Education status
- Religion

Health facility service availability and readiness

Health service delivery

1. Mobilization of resources for the diagnosis and the treatment of COVID-19 patients by the *(insert Country name*) government happened during COVID-19. Did the mobilization involve your facility? How was the facility involved? (Probe community, regional or national level involvement)

   COMMENT: *Indicate if facility was a designated COVID-19 treatment Centre*
2. What was the emergency response plan of your facility during the outbreak?
3. How did the mobilization of resources towards COVID-19 patients impact the service delivery in your facility, including the impact on human resources, space, medical supplies, etc.?
4. Did the mobilization of resources negatively or positively impact service delivery in your facility? Elaborate
5. Was there a set of essential Sexual & Reproductive Health services outlined to be maintained during the lockdown phase of the COVID-19 pandemic?

   PROMPT: Ask for each of the following services – family planning, abortion, post-abortion care, care and support for GBV and STI prevention and treatment
6. Were there disruptions to any of the SRH essential services outlined? What was the nature (services suspended, limited or shifted) of disruptions observed for each of these essential services (if any)?

   PROBES: for causes (lack of personnel, equipment, medicines or reduced demand, physical space), changes in usual service provision (introduction of appointment-based systems, telemedicine, shifting of usual location) and level of disruption (partial/ complete/not disrupted)
7. Do you think the delivery of SRH services during COVID-19 adequately responded to the needs of the clients at that time?

   PROBE: in what way?
8. What has the recovery process been like and what was the turning point?

   NOTE: Only for applicable countries.

   PROBE for coping mechanisms or approaches to ensuring SRH service delivery.

Health workforce （Human resources）

9. How did COVID-19 affect the health status of health workforce, such as doctors, nurses, ambulance drivers and hospital managers etc.? What measures were taken to protect them from the effects of the epidemic?

For peer review only

PROBE: for the provision of PPE, type of PPE, adequacy of supply and frequency of supply.

10. Was there any form of training on COVID-19? PROMPT: Probe for details of the training if provided – What areas of COVID-19? What form did this training take? Who received this training?
11. Were there employees infected? If someone was infected, how did the infection happen? What was the impact on service delivery due to the infection of SRH workers? How did it influence the service delivery?

> **COMMENT:** *In facilities where SRH staff also provide general care in health facility, ask for the impact of an infected clinical staff in the facility even if not SRH-specific*

12. While knowing that they were at higher risk of contracting the virus, did female workers still have to fulfil the obligations such as caring for elders and immediate family (such as children or young dependents)? Is there any other conflict or challenge they faced during COVID-19? Note: Probe for any challenge worth considering in current setting.

Health information system

13. How does the reporting system for infectious diseases run?
    PROMPT: Probe for reporting system for COVID-19.
14. How did this system influence the availability of services in SRH facilities?
15. How did you keep information about the COVID-19 and SRH services flowing during the outbreak with your upper-level medical institutions and potential clients in your community?
    PROBE: how were changes in the delivery of SRH services communicated to clients? How was important COVID-19 information communicated to clients?

Access to essential medicines

16. Was the access to essential medicines, including regular medicines such as birth control pills, contraceptive methods impacted because of the lockdown/shutdown of the city during the COVID-19?

> ***COMMENT***: *Shutdown can be replaced with lockdown/restrictions depending on what the COVID-19 response was in country using this guide*

PROBE: ask about medicines for all the study focus SRH services

17. How has the outbreak affected the access to essential medicines in SRH for the patients and the general population?

PROBE: for each- contraceptive methods, abortion, post abortion care, STI prevention and treatment and GBV care and support

COMMENT: Probe separately for routine medication (such as antibiotics, analgesics etc where applicable)

Health systems financing

18. How did the COVID-19 epidemic affect the availability of funds at your facility?
    PROMPT: Inquire about effect on the financing and maintenance of essential SRH services and possible disruptions due to the pandemic, availability of additional government funding allocated to assuring essential health services.

COMMENT: *This section should be informed by the existing health financing policy (i.e. National health insurance, existing SRH related exemptions and waiver systems) or COVID-19 specific directives on financing.*

Leadership and governance

19. What are your perceptions on the roles and responsibilities of providing SRH services during outbreaks like COVID-19?
    PROBE: where does the primary responsibility to ensure continuity of services lie? Who are the enablers, implementers?
20. How did your facility respond to the emergency and manage to ensure the SRH-related service delivery in your facility?
    PROMPT: Probe for country-specific plan, policies for maintaining SRH service provision during the pandemic.
    PROBE: separately for, family planning, STI and abortion services, services prioritized by the facility during the pandemic.

Health service provider perceptions on the delivery of specific SRH services in the context of COVID-19

A. Gender based Violence

Perceptions of changes in the nature and magnitude of violence against women, and especially domestic violence during COVID-19

- Have you noticed any change in the number of women coming into the health facility reporting gender-based violence since the COVID-19 crisis began?
  - Follow up: Increase or decrease?
  - Follow up: Have the women coming in who report experiencing violence mostly been returning patients, or mostly been new patients?
  - Follow up: Have you noticed an increase in the frequency of abuse that women are experiencing since movement restrictions were put in place?
  - If possible, specify types of violence (physical, sexual, psychological by a partner), non-partner sexual abuse, other

- Do you think that the violence women have been experiencing has become more severe during the crisis, for example has there been an increase in injuries? Violence during pregnancy?
  - Follow up: Have the types of violence that women are reporting changed? How so?

Nature of service provision during COVID-19, whether services for VAW are being changed or adapted and if safety considerations are adequately addressed?

- Since the start of COVID-19, has your health facility changed or adapted services for survivors 'of violence?
  - Probe: (if not mentioned) In some health centers, providers have maintained 'case management' using video calls, or text messages. Did your health center make use of any new technologies to maintain services? If so, did you develop a protocol to ensure safety of survivors?

For peer review only

Access to /use of health services for VAW and related challenges

- Some health centers have had to deal with shortages of personal protective equipment and medications that have impacted their ability to offer services during the COVID-19 crisis. Have there been any new barriers to delivering services to survivors at your health facility?
  - What are these barriers and what approaches are being used to overcome these barriers?

- In a lot of places, external referral systems have also been impacted by the crisis, such as lower numbers of available spaces in shelters due to the need for physical distancing, or NGOs that have been temporarily shut down. To your knowledge, have any changes have been made to your external referral systems? What are these?
  - Follow up: Are any of the services to which you typically refer survivors available in a more limited capacity, or no longer available due to the crisis?

Attitudes towards provision of SRH services in the context of COVID-19

- Do you think it is essential to provide services for VAW during COVID-19? Why do you think so?

B. <u>Induced abortion</u>

Perceptions of changes in the demand for abortion during COVID-19.

- Have you noticed any change in the number of women seeking abortion services at your facility during COVID-19?
  - Follow up: Increase or decrease?
  - Follow-up: any changes in the abortion methods requested?
  - What do you think were the factors driving this change?

Delivery of safe abortion services during COVID-19?

- Where and how can people seek contraceptive services during COVID-19?
  - How many methods of abortion were available in your health facility during COVID-19? Which method of abortion did you suggest during COVID-19? Why?
  - What challenges did you meet in providing safe abortion services during COVID-19? What approaches did you use to overcome these challenges?

Attitudes towards provision of SRH services in the context of COVID-19

- Do you think it is essential to provide safe abortion services during COVID-19? Why do you think so?

C. <u>Contraceptive services</u>

Perceptions of changes in the demand for contraception services during COVID-19.

- Have you noticed any change in the number of women seeking contraception services at your facility during COVID-19?
  - Follow up: Increase or decrease?
  - Follow-up: any changes in the contraception methods requested?
  - What do you think were the factor driving this change?

Delivery of contraception services during COVID-19?

- Where and how can people seek contraceptive services during COVID-19?

For peer review only

- How many kinds of contraceptive methods were available in your health facility during COVID-19?
- What are the new/ altered ways by which contraceptive services are provided as a response to COVID-19?
- What challenges did you meet in providing contraceptive services during COVID-19? What approaches did you use to overcome these challenges?

Attitudes towards provision of SRH services in the context of COVID-19

- Do you think it is essential to provide contraception services during COVID-19? Why do you think so?

D. <u>Prevention and treatment for sexually transmitted infections</u>

Perceptions of changes in the demand for care and treatment for STI during COVID-19.

- Have you noticed any change in the number of women seeking care and treatment for STI at your facility during COVID-19?
    - Follow up: Increase or decrease?
    - What do you think were the factor driving this change?

Availability of STI care and treatment during COVID-19

- Where and how can people seek care and treatment for STI during COVID-19?
  - What are the new/ altered ways by which care and treatment for STI services are provided as a response to COVID-19?
  - Were all the medicines and supplies for providing the care and treatment available during COVID-19?
  - What challenges did you meet in providing care and treatment for STI services during COVID-19? What approaches did you use to overcome these challenges?

Attitudes towards provision of SRH services in the context of COVID-19

- Do you think it is essential to provide prevention and treatment for STIs during COVID-19? Why do you think so?

3. Perceived psychosocial effects of COVID-19
   - Did you experience stress/anxiety/depression during COVID-19?
   - How would rate your psychosocial health?
   - What role did COVID-19 play in influencing your psychosocial health?
   - In what way?
   - What do you think is the psychosocial impact of COVID-19 on people?
   - Do you think the impact was the same for men and women? If yes, why?
   - If no, in what ways is the psychosocial impact different?
   - What do you think is the psychosocial impact of COVID-19 on families?
   - What do you think is the psychosocial impact of COVID-19 on communities?

4. Final questions
   - On-going concerns about COVID-19 and its possible impact
     - Are there any other concerns about COVID-19 and its impact on you, your family or in general that you wish to express?
   - Needs related to accurate information (probe further information needs – NB consider how to provide information if necessary)

For peer review only

- Was accurate information readily available and accessible during COVID-19?
- Was it easy to understand?
- Was the information that was available adequate?
- Was there additional information you would have liked to obtain but was missing?
- Are there better ways of making accurate information accessible to you and others?

- Needs related to sexual reproductive health services (probe further SRH needs – NB consider how to support/provide if necessary)
- Do you think SRH services were responsive to the needs of patients during COVID-19?
- If no, which of needs were not met?
- Which SRH services are most important, that should be available even in health emergencies like COVID-19?
- How do you think provision of SRH services can be supported during other health emergencies like COVID-19? E.g. barriers in seeking SRH services during COVID

Additional themes will be added as appropriate for each target group and questions revised accordingly (with attention to first person / third person views), and across the three phases of the study.

For peer review only

BMJ Open

Health systems analysis and evaluation of the barriers to availability, utilization and readiness of sexual and reproductive health services in COVID-19 affected areas: A WHO mixed methods study protocol

Journal:	*BMJ Open*
Manuscript ID	bmjopen-2021-057810.R4
Article Type:	Protocol
Date Submitted by the Author:	22-Apr-2022
Complete List of Authors:	Kouanda, Seni; Institut de Recherche en Sciences de la Sante; Institut Africain de Sante Publique<br>Nahyuha Chomi, Eunice; Research Institute of Health Sciences<br>Kim, Caron; World Health Organization, Department of Sexual and Reproductive Health and Research. Preventing Unsafe Abortion Team<br>Jen, Sothornwit; Khon Kaen University, Department of Obstetrics and Gynaecology<br>Bahamondes, Luis; State University of Campinas – UNICAMP, Obst & Gynecol<br>Cecatti, Jose; University of Campinas, Obstetrics and Gynecology<br>Lumbiganon, Pisake; Khon Kaen University, Faculty of Medicine<br>Emefa, Modey; University of Ghana<br>Brizuela, Vanessa; WHO, Department of Reproductive Health and Research<br>Kuganantham, Hamsa; World Health Organization, Department of Reproductive Health and Research<br>Seuc, Armando; World Health Organization<br>Ali, Moazzam; Organisation mondiale de la Sante,
**Primary Subject Heading**:	Public health
Secondary Subject Heading:	Health policy, Health services research, Infectious diseases
Keywords:	COVID-19, PUBLIC HEALTH, Reproductive medicine < GYNAECOLOGY

SCHOLARONE™
Manuscripts

Manuscript Protocol.

Health systems analysis and evaluation of the barriers to availability, utilization and readiness of sexual and reproductive health services in COVID-19 affected areas: A WHO mixed methods study protocol

- Kouanda, Seni-Reproductive Health Unit, Institute for Research in Health Sciences, Ouagadougou, Burkina Faso
- Chomi, Eunice- Reproductive Health Unit, Institute for Research in Health Sciences, Ouagadougou, Burkina Faso
- Kim, Caron- Department of Reproductive Health and Research, World Health Organization, Avenue Appia 20, 1211 Genève 27, Switzerland
- Sothornwit, Jen- Department of Obstetrics and Gynaecology, Faculty of Medicine, Khon Kaen University, Khon Kaen, Thailand.
- Bahamondes, Luis- School of Medical Sciences, University of Campinas (UNICAMP), Campinas, Brazil
- Cecatti, Jose Guilherme- School of Medical Sciences, University of Campinas (UNICAMP), Campinas, Brazil
- Lumbiganon, Pisake- Faculty of Medicine, Khon Kaen University, Khon Kaen, Thailand
- Modey, Emefa-Department of population, family and Reproductive Health, University of Ghana, Legon-Accra, Ghana.
- Brizuela, Vanessa- Department of Reproductive Health and Research, World Health Organization, Avenue Appia 20, 1211 Genève 27, Switzerland
- Kuganantham, Hamsadvani- Department of Reproductive Health and Research, World Health Organization, Avenue Appia 20, 1211 Genève 27, Switzerland
- Seuc, Armando Humberto, Consultant, Department of Reproductive Health and Research, World Health Organization, Avenue Appia 20, 1211 Genève 27, Switzerland
- Ali, Moazzam* on behalf of WHO HRP social Science Research Team**- Department of Reproductive Health and Research, World Health Organization, Avenue Appia 20, 1211 Genève 27, Switzerland

*Corresponding author: Moazzam Ali (alimoa@who.int). Telephone: +41-(0)22-791-3442 Department of Reproductive Health and Research, World Health Organization, Avenue Appia 20, 1211 Genève 27, Switzerland.

**** WHO HRP Social Science Research Team**: **Brazil** (Luis Bahamondes, Jose Guilherme Cecatti); **Burkina Faso** (Eunice Chomi, Seni Kouanda); **China** (Kun Tang, Hanxiyue Zhang, Yifan Zhu, Yueping Guo, Ge Yang and Chunxiao Peng); **Ghana** (Deda Ogum Alangea, Kwasi Tropsey, Emefa Judith Modey); **Pakistan** (Rozina Karmaliani, Laila Ladak); **Thailand** (Pisake Lumbiganon, Jen Sothornwit); **Kenya** (Marleen Temmerman, Abdu Mohiddin, Ferdinand Okwaro); **Italy** (Massimo Mirandola, Maddalena Cordioli, Alessia Savoldi, Simone Garzon, Stefano Uccella, Ranieri Poli.) **UK** (Nigel Sherriff, Alexandra Sawyer, Jorg Huber, Jaime Vera, Debbie Williams).

For peer review only

WHO Secretariat (Moazzam Ali, Caron Kim, Hamsadvani Kuganantham, Igor Toskin, Vanessa Brizuela, Anna Thorson, Joy Jerop Chebet, Hugo Gamerro Abrego , Soe Soe Thwin, Armando Seuc)

Word count of text (excluding title page, abstract, references, figures and tables)- 4345

KEYWORDS
Contraception, sexual and reproductive health, abortion services, COVID-19, facility assessment, sexual and gender-based violence, sexually transmitted infections

For peer review only

For peer review only - http://bmjopen.bmj.com/site/about/guidelines.xhtml

Abstract

Introduction The novel Corona Virus Disease 2019 (COVID-19) has led to an unprecedented increase in demand on health systems to care for people infected, necessitating the allocation of significant resources, especially medical resources, towards the response. This, compounded by the restrictions on movement instituted may have led to disruptions in the provision of essential services, including sexual and reproductive health (SRH) services. This study aims to assess the availability of contraception, comprehensive abortion care, sexually transmitted infection (STI) prevention and treatment and sexual and gender-based violence (SGBV) care and support services in local health facilities during COVID-19 pandemic. This is a standardized generic protocol designed for use across different global settings.

Methods and Analysis This study adopts both quantitative and qualitative methods to assess health facilities' SRH service availability and readiness, and clients' and providers' perceptions of the availability and readiness of these services in COVID-19-affected areas. The study has 2 levels: (1) Perceptions of clients (and the partners) and healthcare providers, using qualitative methods, and (2) assessment of infrastructure availability and readiness to provide SRH services through reviews, facility service statistics for clients and a qualitative survey for healthcare provider perspectives. The health system assessment will use a cross-sectional panel survey design with two data collection points to capture changes in SRH services availability as a result of the COVID-19 epidemic. Data will be collected using focus group discussions, in-depth interviews and a health facility assessment survey.

Ethics and Dissemination Ethical approval for this study was obtained from the World Health Organisation (WHO) Scientific and Ethics Review Committee (protocol ID CERC.0103). Each study site is required to obtain the necessary ethical and regulatory approvals that are required in each specific country.

Strengths and limitation of this study

- The study approach and design enable a comprehensive analysis of the barriers to availability, utilization and readiness of sexual and reproductive health services during COVID-19, as well as the post-pandemic recovery as transmission is contained.
- The use of a mainly qualitative approach places women's rights and needs during health emergencies at the center of the debate, underscoring the need for more responsive policies.
- The inclusion of partners will enhance understanding of gender dynamics and support efforts towards identifying strategies to enhance positive male involvement and engagement in women's sexual reproductive health and rights.
- A possible limitation is that the study will be conducted during and after one of the waves of the pandemic, at which stage there may have been changes in the health system as lessons are learnt.
- Another limitation is that the estimation of the recovery period may be difficult in settings where the pandemic is yet to be brought under control. This part of the analysis may be subject to delays until an appropriate period is determined.

For peer review only - http://bmjopen.bmj.com/site/about/guidelines.xhtml

INTRODUCTION

The novel COVID-19 disease, which was first reported in December 2019 and rapidly spread globally, has led to an unprecedented increase in demand on health systems to care for people infected, necessitating the allocation of significant resources, especially medical resources, towards the response. The increased demand, compounded by the restrictions on movement instituted as part of containment measures may have led to disruptions in the provision of essential services, including sexual and reproductive health (SRH) services [1,2].

The novel coronavirus is new to humans and only limited scientific evidence is available on the impact of COVID-19 on SRH service delivery. However, lessons from the Ebola and Zika virus outbreaks have highlighted the severe disruptions in SRH services that expose women and girls in particular to preventable health risks [2,3]. Some services may be unavailable due to either facilities and health workers being repurposed to care for COVID-19 patients, patient safety concerns, movement restrictions disrupting travel to health facilities, supply chain disruptions or a reduction in health workers because of increasing numbers being themselves infected by COVID-19 [4,5]. In addition, overwhelmed with COVID-19 cases, clinical staff may not have the time or personal protective equipment (PPE) needed to provide family planning counselling and commodities [5]. Recent evidence suggests disruptions lasting 3-6 months in 2020 left between 4-23 million women in low- and middle-income countries unable to access modern contraceptives, a projected 1.4 million (500,000-2.7 million) unintended pregnancies and an additional 31 million cases of secual and gender-based violence (SGBV) [1, 6-9]. Furthermore, studies have modelled the potential impact, showing that even a 10% reduction in essential SRH services could lead to an estimated 15 million unintended pregnancies, 3.3 million unsafe abortions and 29, 000 additional maternal deaths during the next 12 months [7-10]. Continuity of essential health services while keeping people safe during the response to disease outbreaks such as the COVID-19 pandemic is therefore essential for the prevention of both direct and indirect mortality [4,5].

This is a generic standardized protocol designed to maximize the likelihood that data are systematically collected and shared rapidly in a format that can be easily reproducible, aggregated, tabulated and analyzed across many different settings globally and be useful as templates for use in health emergencies in the future. This will facilitate the comparison of results across regions and countries and will potentially improve the quality of observational studies by identifying and minimizing biases. Given that use in different settings will require some adaptation, these possibilities have been highlighted throughout the protocol.

The introduction should be updated with country specific data on COVID-19 epidemiology and current research findings prior to submission to local/country national institutional review boards

For peer review only - http://bmjopen.bmj.com/site/about/guidelines.xhtml

The main aim of this study is to assess the availability of contraception, comprehensive abortion care, STI prevention and treatment (including HIV) and SGBV care and support services in local health facilities during COVID-19 pandemic. The four specific objectives of the study are:

1. To explore the availability of, and health facility readiness to provide these services in areas most affected by COVID-19.
2. To assess the availability and quality of services and barriers to the utilization of these services from clients' and providers' perspectives in the selected COVID-19 affected areas.
3. To assess the post-pandemic recovery* of the facilities in the provision of these services in comparison to the pandemic period.
4. To enhance the SRH service capacity in COVID-19 through advocacy, policy briefs, media dissemination and academic papers towards national and regional stakeholders including policymakers, academia, healthcare providers and the community.

**Post-pandemic recovery refers to the period when ideally health facilities have been able to recover from the disruption in service provision, following reduced transmission levels. Caution should be applied to the term "post-pandemic" as services may be still impacted even with lower levels of transmission. Therefore, the research team should be cognizant of the distinct time periods in their facility assessments.*

METHODS AND ANALYSIS

Study design

This is a repeated cross-sectional study, using both quantitative and qualitative methods. The two data collection points, baseline and end-line, will be 9 to 12-months apart. The aim of having two data collection points is to document and share the local evidence with authorities on the SRH services at baseline, and then at end line to track and demonstrate the changes and improvement in services over time. The World Health Organisation (WHO) situational level assessment of COVID-19 transmission will be used as the basis for determining when to conduct baseline and endpoint data collection [11]:

- Level 0: no known transmission in the preceding 28 days, no restriction on daily activities.
- Level 1: basic measures in place, clusters of cases reported controlled with basic measures, limited and transient disease.
- Level 2: local transmission from imported cases to close contacts. Contact tracing, physical distancing and quarantine measures can contain the spread.
- Level 3: community transmission, where source of infection is untraceable, outbreak rapidly spreads in clusters.
- Level 4: disease outbreak has become an epidemic, where there are major clusters of infection all over the country, high number of deaths and it is very difficult to control transmission without strict containment measures.

Countries should time the baseline and end-line data collection as follows: Baseline data collection is proposed when countries are experiencing level 3 or 4 transmission (referred to here as peak transmission/pandemic period), as disruptions in service provision are likely to occur during these periods. End-line data collection is proposed when countries have tentatively

For peer review only

managed to contain the spread and are at levels 0,1 or 2 (referred to here as post-pandemic period/after the pandemic) after experiencing levels 3 or 4.
Given the dynamic nature of the epidemic, where each country is experiencing varying levels of COVID-19 transmission (and the challenges from the resurgence of variants and sudden increase in the number of cases), it is difficult to assume standardized pandemic conditions at country level. Therefore, the time interval between data collection points is just an estimation. For countries that have contained the spread, it may be decided to collect baseline data retrospectively, selecting the peak period. However, strategies to minimize or avoid recall bias should be used.

Study Setting

The study will be conducted in geographical areas selected based on the epidemic status. To get variation in responses, health facilities will be selected as focal points considering their qualification to provide SRH services and other criteria for the researchers to access community members for participation in the study. It is expected that variation in health facilities distributed within different geographical areas will provide access to communities of all socioeconomic backgrounds[1].

Due to various local realities and political factors, countries should select the research study sites based on geographical location, organization of SRH service delivery and epidemic status where COVID-19 is likely to have significantly affected service delivery (given that even within countries the transmission status differs, hence the differential impact on facilities in different geographical areas). Consultation with the Ministry of Health will help identify the areas most affected by COVID-19.

Study Population

The study population will be women seeking SRH services from the selected health facilities and the partners, who will be from different households.This technique will offer a measure of protection for those women who may be at risk just because they participated in the study.

In contexts where it is not customary for men to accompany their partners when they seek SRH services, other options can be used to access them. Healthcare providers can be medical doctors, nurses, midwives, nurse assistants, allied health professionals and other cadres depending on the norms and standards for the provision of SRH services in different country contexts.

Sample selection

[1] Countries included are: Brazil, Burkina Faso, China, England, Ghana, Italy, Kenya, Pakistan, Thailand

For peer review only - http://bmjopen.bmj.com/site/about/guidelines.xhtml

Each sample will be selected based on specific criteria, as well as specific country contextual factors.

Health facility selection

The minimum criteria for selection should include availability of human resources, primarily the qualification to provide contraception, safe abortion, including the treatment of complications and the provision of post-abortion care, STI care and treatment and support for women experiencing SGBV. In addition, the diversity of health facility capacity, administrative rank, urban/rural setting and the willingness of the providers in charge to participate in the study, as well as how the COVID-19 response has been organized in terms of treatment centers. These criteria for selection of health facilities within the geographical sites were made to encourage a representative mix of facilities. The same health facilities will be used for both data collection points to highlight the changes in SRH delivery due to COVID-19 and post-pandemic recovery.

Given the dynamic nature of the pandemic *some flexibility should be given to all study sites in the selection of facilities, maintaining the minimum criteria specified above*

Qualitative sample

Selection of women

Women will be recruited for in-depth interviews (IDIs) and focus group discussions (FGDs). The women will be purposively selected to obtain a sample of women and the partners. Selection will be based on 1) being of reproductive age (18-49) and 2) having sought or tried to receive SRH services from local health facilities. The study will use gatekeepers, who will be healthcare provides not involved in the study to approach the women as they leave the health facility. Their role will be limited to the identification and introduction of potential participants to the study team.

For the IDIs, a sample of 10~15 women (or until saturation) and 6~12 partners (or until saturation) will be recruited. For countries with more enrolled health facilities in the study, the maximum number of the IDIs will be with 10 women (or until saturation) at reproductive age seeking for reproductive healthcare services, and with 5 partners (or until saturation) per health facility. This will be done as an exit interview and the women will be consecutively selected until the desired sample (or saturation) has been achieved.

For FGDs, gatekeepers will also ask participants about their level of comfort discussing experience seeking SRH issues in a group before being recruited. About 6-8 participants per FGD will be recruited, with the expectation of at least two focus groups per facility. Some female and male participants will also be invited for individual interviews after the FGDs.
This is also to note that by interviewing husbands and wives will be from different households; by not interviewing husband-wife from the same household will operationalize the concept of "do no harm" and offer a measure of protection for those women who may be at risk just because they participated in the study.

For peer review only

The ages can be adjusted to account for differences in the nationally recognised reproductive age group in different contexts. Inclusion of girls younger than 18 years should take into consideration ethical issues of assent and consent.

To adjust to challenges posed by COVID-19 transmission, additional participants can be recruited through online recruitment from chat groups such as Facebook, Twitter or WhatsApp. Careful attention must be paid to the ethical issues of privacy and confidentiality of participants and integrity of the researchers (transparency of aims, details, risks and benefits of the study, obtaining the necessary permission to join restricted groups and the presence of the researcher in the group). Other challenges of online recruitment that need attention include retention of participants, potential selection bias, verification of participant identity and comprehension of informed consent [12]. Care needs to be taken by hiding the names and faces (e.g., via video conference calls) of the online participants, responsible handling of participants' personal information to minimize the likelihood of embarrassment, loss of dignity or harm because of the online recruitment process to address the online privacy and confidentiality concerns. The moderators will ask the participants to give a short self-introduction, using pseudonyms/numbers, instead of their actual names, and not using video options. This includes not disclosing any of this information without the participants consent.

Selection of healthcare providers

Approximately 1 to 2 healthcare providers per health facility will be purposively selected and only those who 1) deliver SRH services, 2) are most knowledgeable about readiness and availability of SRH services 3) are stationed in the SRH clinic and have been working at the clinic for at least 6 months before the pandemic started will be selected for inclusion in the study. This information will be obtained from the facility in-charges.

Quantitative sample

Selection of Women

In each health facility, a maximum of 3 clients from each section (post-abortion care, family planning, SGBV, STI, abortion) will be consecutively selected to achieve up to 20 clients per health facility.

Selection of healthcare providers

One healthcare provider, preferably most knowledgeable in the health facility about SRH services provided, as determined by seniority, position or function, for example, will be selected to assist in the health facility assessment.

Data Collection

FGDs and IDIs will be used to understand client's perspectives on their experiences in accessing SRH services during and after peak transmission and healthcare providers' perspectives of SRH service availability and readiness in COVID-19-affected areas. The health system assessment will

For peer review only - http://bmjopen.bmj.com/site/about/guidelines.xhtml

be used to assess the health system response to COVID-19. Study teams should be aware of the possibility that healthcare provider burnout or heavy workload may affect willingness or ability to participate in the study. Adaptations to data collection should be made to avoid overburdening the healthcare providers and clearly explained during recruitment.

Data Collection tools

There will be five sets of tools for clients- 1 FGD guide for women (published as **supplementary file S1**), 1 FGD guide for partners/men (published as **supplementary file S2**), 1 interview guide for women (published as **supplementary file S3**), 1 interview guide for men (published as **supplementary file S4**) and and 1 interview guide for healthcare providers (published as **supplementary file S5**).

For the facility assessment, a facility and readiness assessment questionnaire has been developed based on the following validated tools [13-16]:

(1) WHO Service Availability and Readiness Assessment (SARA) guide
(2) WHO Health Facility Readiness Checklist
(3) WHO safe abortion assessment tool
(4) SGBV Secual and gender-based violence Quality Assurance Tool

The facility and readiness assessment tool comprises five modules: (1) health services continuation; (2) family planning services; (3) abortion services; (4) STI and (5) SGBV; in line with the study SRH focus areas. The following indicators will be used the assess the availability and readiness:

(1) Policies and plans
(2) Service maintenance and referrals
(3) Infrastructure
(4) Commodities
(5) Human resources

Each study site will adapt (including translation to local language) the tool to its specific context, taking into consideration the existing policies, SRH treatment guidelines, staffing norms and standards, types of facilities, national essential medicines lists and national health information systems among others. After adaptation to specific country context all tools should be piloted before being used.

Data Collection Methods

Participant Informed Consent Process

All study participants will be taken through a detailed informed consent process (for participation and audio-recording), which will be documented and those who agree to participate in the study will be asked for signed written (oral consent for those who cannot write, or for situations that necessitate online data collection) consent.

Given that data collection will be conducted during COVID-19, an assessment of the risks to the study team and participants should be made. The decision of whether to conduct face-to-face

For peer review only - http://bmjopen.bmj.com/site/about/guidelines.xhtml

or remote data collection should be based on the risk assessment, followed by context-specific recommendations on adaptations to the original data collection process. Remote data collection will necessitate adaptation to the length of interviews and discussions, rapport building, oral informed consent, privacy and confidentiality measures as well as making special arrangements where access to internet and mobile connectivity is limited [17]. Face-to-face data collection will require special arrangements to ensure the safety of the study team and participants, following WHO as well as country-specific safety protocols, including educating and training the research team and participants about COVID-19, provision of PPE and hygiene supplies, mandatory hygiene practices and sanitization of venues and equipment and physical distancing [18].

The FGDs and IDIs will be conducted at times and in venues that are considered both convenient and safe for the participants to freely discuss the subject matters. In addition, qualified researchers with experience in conducting IDIs and facilitating FGDs will be trained to ensure the validity of the data collection and will be selected based on the gender of the participants. The FGDs and IDIs will be semi-structured and follow a topic guide specific to each group of participants but will take place as a conversation in which the researchers promote a safe, comfortable environment to enable a comprehensive and candid record. In addition to the semi-structured interview, narrative interview techniques may be used depending on the participants' narratives about life events and reproductive healthcare needs.

The FGDs and IDIs will be audio-recorded. Prior consent for recording will be sought again from each participant. Anonymity of the participants will be ensured by removing any personal or family identifiers and all recorded sessions will be coded for purposes of identification with a date, geographical site and session number. After each session, the recording will be sent to the team supervisor for secure storage for transcription. The audio files will be encrypted and sent to an electronic database which will be shared with WHO. Therefore, only the research team will be authorized to listen to the recordings. These audio recordings will be retained until they have been transcribed and checked for accuracy, after which they will be destroyed.

The FGDs will explore knowledge about COVID-19, care-seeking during COVID-19 and risk perceptions and availability of SRH services (end-line FGD will also explore post-pandemic services in comparison to the pandemic period). Before starting the FGD, the facilitators will collect socio-demographic data for each participant, build rapport with and among participants and set ground rules to ensure positive group dynamics that foster an effective and rich discussion. The facilitators will also use this time to know and understand the emic categories (field research and viewpoints obtained from within the social group, from the perspective of the subject) used by locals to describe their perceptions and practices related to the study topics. Each FGD is expected to last approximately 60 – 90 minutes.

IDIs with women will explore the psychosocial effects of COVID-19 on fertility desires, knowledge of COVID-19, risk perceptions and concerns about effects on SRH, care seeking behaviors, experience and barriers in accessing SRH services reproductive health, particularly comprehensive abortion, STI prevention and treatment and SGBV care and support, and their related needs for accurate information and reproductive health services. IDIs with partners will

For peer review only - http://bmjopen.bmj.com/site/about/guidelines.xhtml

explore their knowledge of COVID-19, risk perceptions and concerns about effects on SRH, their influence on the access to SRH services of the partners and find out what role they play when the partners require SRH services like contraception, comprehensive abortion care, STI prevention and treatment and SGBV care and support. Each interview is expected to last approximately 40 – 60 minutes. IDIs for the women and the partners will be conducted separately.

In some countries, abortion is illegal, posing challenges in data quality since participants, fearing reprisals, may not provide accurate responses to abortion-related questions. Necessary precautions should be taken to ensure the interviews are conducted in a supportive and non-judgmental manner, encouraging the participants to respond freely. These include selection and training of interviewers to enable them to overcome their biases and stereotypes about abortion, building trust and rapport with participants and selection of interview venue to guarantee privacy [19].

Given the sensitive nature of the data being collected, care must be taken in the selection of data collectors to ensure protection of participants. The gender, experience and attitudes of the data collectors are important considerations. Training of data collectors is essential with emphasis on the risks to the participants and how to protect them, the importance of non-judgmental attitudes and provision of necessary support to participants.

Interviews with healthcare providers will be based on WHO's six building blocks framework [20 with a focus on the delivery of the focus SRH services during COVID-19; their perceptions on the roles and responsibilities of different parties to provide these services in the COVID-19 context; health system capacity to provide good quality of care for people during COVID-19; training needs, attitudes, biases about contraception, abortion, STI and SGBV in the context of COVID-19; and perceived psychosocial effects on men and women, their families and local communities.

The health availability and readiness assessment will be implemented in all the selected facilities using a cross-sectional survey design to highlight gaps or service delivery issues during COVID-19 and recovery in service availability and readiness in the same health facilities. The assessment will also include a review of health facility records in family planning and contraception, STI care and treatment and care and support for women who have experienced SGBV to assess the availability, type and range of commodities offered. A follow-up assessment will be done at end-line. The follow-up survey plans to assess the recovery in service availability and readiness in the same health facilities. The research team will fill out the data extraction tool and the questionnaire with the assistance of the most knowledgeable person in the health facility about SRH services provided (senior health care provider and/or administrator) for each section of the questionnaire. The assessment will also include a questionnaire to the clients of the health facility.

The client questionnaire contains questions related to general characteristics of clients and their experience in seeking SRH services. All the selected health facilities will be required to collect this data from all clients seeking SRH services once every month throughout the study period, to capture trends in service availability and utilization.

For peer review only - http://bmjopen.bmj.com/site/about/guidelines.xhtml

Data Analysis

Health facility assessment data will use the presence of the tracer items for the provision of these services, such as availability of guidelines, staff and essential commodities. Data will be entered into an electronic database using data entry programmes such as Epi data and CSPro. Study sites using electronic data collection may skip this step and for WHO supported centres implementing the study an online platform (OpenClinica) will be used. Descriptive analysis will be used to illustrate the basic characteristics of the different facilities, including the monthly number of clients, types of procedures provided, number of medical staff, stocks of drugs, etc.

Qualitative analysis will form the main analysis for this study, beginning while data collection is ongoing to assess progress and determine any problems. The audio-recorded data will be transcribed verbatim and de-identified by using ID numbers in place of names. Where required, transcriptions will be translated to English and back-translated and analyzed using content analysis, according to the suggested steps by Elo & Kyngäs (2008) [21], as illustrated in Table 1. The WHO team based in Geneva, in conjunction with the study principal investigator (PI) and project team, will support and monitor the data analysis.

Table 1. Planned analytical procedures for content analysis

Phase	Procedures
Preparation	Reading through verbatim transcriptions of the interviews several times to familiarize with the data, gain an understanding of what has been expressed, selecting the unit of analysis, deciding on the analysis of manifest content.
Organizing	Open coding and creating categories, grouping codes under higher order headings, formulating a general description of the research topic through generating categories and subcategories as abstracting.
Reporting	Reporting the analysis process and the results through models, conceptual systems, conceptual map or categories, and a story line.

Data management and access

Data management plans should include information about how data will be stored, including levels of protection, who will have access to the data and when it would be destroyed, how data will be transferred (if needed) securely. In countries where data protection legislation exists, protocols should specify that data would be handled in accordance with those policies.

Quantitative data

Data collectors will be trained on data collection, transmission, verification, storage and primary analysis to assess errors. Data can be collected electronically, or on paper.

Qualitative data

For peer review only - http://bmjopen.bmj.com/site/about/guidelines.xhtml

The quality and trustworthiness of the qualitative data collected will be assured through triangulation, as we will conduct interviews with different parties, including health professionals, health workers, and clients. The process of the study will be clearly documented to ensure the transparency and the rigor of the study.

Ethics and dissemination

Scientific approval has been obtained from WHO research project review panel (RP2). Ethical approval for this study was also obtained from the WHO Ethics Review Committee (protocol ID CERC.0103). Each study site is required to obtain the necessary ethical and regulatory approvals that are required in the corresponding country. Ethical considerations of informed consent, voluntary participation, privacy and confidentiality, anonymity and compensation for incidental costs (the decision to offer compensation and its value will depend on the specific country context and respective local policies) will be respected and detailed in the informed consent process.

Given the sensitive nature of the data being collected, special care will be taken to ensure the "do no harm" principle is respected. This will include making arrangements with locally available support services (medical, psychosocial, legal) where participants can be referred, training the research team to respond to and provide immediate emotional support to study participants or for situations where support services are not locally available or are inadequate [22, 23].

At country level the results will be presented to policy makers, researchers, managers through policy briefs and workshops in collaboration with the WHO office. The results will also be disseminated among the communities/participants through online platforms/text messages, which will be provided to the participants by the data collectors/health workers. In addition, the results will also be presented to the scientific and funding community in collaboration with WHO country and regional offices, by communication and manuscript publications in peer-reviewed international journals

Discussion

This is a generic standardized protocol designed to be used across many different settings globally and to be useful as templates for use in health emergencies in the future. This will facilitate the comparison of results across regions and countries.

This study provides a unique opportunity to assess the availability, utilization, and readiness of the SRH services during the COVID-19 pandemic. The main lesson learned will be the adaptations of the health system in pandemic situations and what can be done to ensure continuity of essential SRH services.

The mixed methods approach and panel design with two data collection points enable a comprehensive analysis of the barriers to availability, utilization and readiness of sexual and reproductive health services during COVID-19, as well as the post-pandemic recovery as transmission is contained.

For peer review only - http://bmjopen.bmj.com/site/about/guidelines.xhtml

This study is well placed to advocate for the development and strengthening of policies and services that are responsive to the needs of women and girls during health emergencies, given the potential to exacerbate existing gender and social inequalities and increase the vulnerability of women and girls to preventable health risks. The use of a mainly qualitative approach places women's rights and needs at the center of the debate. The inclusion of partners will enhance understanding of gender dynamics and support efforts towards identifying strategies to enhance positive male involvement and engagement in women's sexual reproductive health and rights.

Contributor ship statement

Contributors: MA and CK conceptualized and designed the protocol. All authors contributed to developing the detailed methodology. SK, EC and MA drafted the first draft manuscript. SJ, BL, CJG, LP, ME, BV, KH, and SA edited the draft, provided critical inputs, and approved the final written version for publication.

Funding Statement

This research has been supported by German Federal Ministry of Health (BMG) COVID-19 Research and development funding to WHO. It has also been supported by the UNDP-UNICEF-WHO-World Bank Special Programme of Research, Development and Research Training in Human Reproduction (HRP); a co-sponsored programme executed by the World Health Organisation (WHO). The adaptation and implementation of this protocol in the HRP collaborating centres in Brazil, Burkina Faso, China, Ghana, Italy, Kenya, Pakistan, Thailand and the United Kingdom was also funded by the same. The views of the funding body have not influenced the content of this manuscript. This article represents the views of the named authors only and does not represent the view of their affiliated institutions.

Disclaimer

This report contains the collective views of an international group of experts and does not necessarily represent the decisions or the stated policy of the World Health Organization.

Data Sharing Statement

Data sharing not applicable as no datasets generated and/or analysed for this protocol.

Competing interests

The authors declare that they have no competing interests.

Patient and public involvement

No patient or public involvement.

Acronyms

COVID-19	Corona Virus Disease 2019
CSPro	Census and Survey Processing System
FGD	Focus Group discussion

For peer review only

SGBV Gender Based Violence
IDI In-depth Interview
RP2 Project Review Panel
SRH Sexual and Reproductive Health
STI Sexually Transmitted Infections
WHO World Health Organisation

References

1. Riley T, Sully E, Ahmed Z, Biddlecom A. Estimates of the potential impact of the COVID-19 pandemic on sexual and reproductive health in low- and middle-income countries. *Int Perspect Sex Reprod Health* 2020; 46:73–76. doi: 10.1363/46e9020.
2. Addressing human rights as key to the COVID-19 response. Geneva: World Health Organization; April 2020 (WHO/2019-nCoV/SRH/Rights/2020.1; https://www.who.int/publications/i/item/addressing-human-rights-as-key-to-the-covid-19-response (accessed August 15, 2020)
3. Sexual and Reproductive Health and Rights during the COVID-19 pandemic: A joint report by EPF & IPPF EN. April 2020. https://www.ippfen.org/sites/ippfen/files/2020-04/Sexual and Reproductive Health during the COVID-19 pandemic.pdf (accessed August 16, 2020)
4. COVID-19: operational guidance for maintaining essential health services during an outbreak: interim guidance. 25 March 2020. Geneva: World Health Organization. 2020 WHO/2019-nCoV/essential_health_services/2020.1; https://apps.who.int/iris/handle/10665/331561 (accessed on August 15, 2020).
5. Maintaining essential health services: operational guidance for the COVID-19 context. World Health Organisation; 2020. https://apps.who.int/iris/handle/10665/332240 (Accessed August 15, 2020)
6. Impact of the COVID-19 pandemic on family planning and ending secual and gender-based violence, female genital mutilation and child marriage: pandemic threatens achievement of the transformative results committed to by UNFPA. New York, United Nations Population Fund; 2020 https://www.unfpa.org/resources/impact-covid-19-pandemic-family-planning-and-endinggender-based-violence-female-genital-mutilation (accessed on August 15, 2020).
7. Millions more cases of violence, child marriage, female genital mutilation, unintended pregnancy expected due to the COVID-19 pandemic [Internet]. Unfpa.org. 2020 [cited 3 May 2020]. Available from: https://www.unfpa.org/news/millions-more-cases-violence-child-marriage-female-genital-mutilation-unintended-pregnancies#
8. Coronavirus Disease (COVID-19) Pandemic UNFPA Response Plan. June 2020 https://www.unfpa.org/sites/default/files/resource-pdf/UNFPA_Global_Response_Plan_Revised_June_2020_.pdf (accessed August 16, 2020)
9. United Nations Population Fund. Impact of COVID-19 on family planning: What we know one year into the pandemic. Technical note. New York, UNFPA;2021
10. Julia Hussein. COVID-19: What implications for sexual and reproductive health and right globally? *Sex Reprod Health Matters* 2020;28(1):1746065 DOI:10.1080/26410397.20201756065.

For peer review only - http://bmjopen.bmj.com/site/about/guidelines.xhtml

11. World Health Organization. Considerations for implementing and adjusting public health and social measures in the context of COVID-19. Interim guidance. Geneva, World Health Organization;2020 https://www.who.int/publications/i/item/considerations-in-adjusting-public-health-and-social-measures-in-the-context-of-covid-19-interim-guidance (Accessed December 5, 2020)
12. Gelinas L, Pierce R, Winkler S, et.al. Using social media as a research recruitment tool: ethical issues and recommendations. *Am J Bioeth 2017*;17(3):3-14
13. World Health Organization. Service availability and readiness assessment (SARA): implementation guide. Geneva: World Health Organization; 2015 http://www.who.int/healthinfo/systems/sara_implementation_guide/en/. (Accessed August 20, 2020)
14. World Health Organization. Safe abortion: technical and policy guidance for health systems. 2nd ed. Geneva: World Health Organization; 2012 http://www.who.int/reproductivehealth/publications/unsafe_abortion/9789241548434/en/. (Accessed March 10, 2021)
15. JHPIEGO. 2018. Secual and gender-based violence Quality Assurance Tool: Standards for the Provision of High-Quality Post-Violence Care in Health Facilities. https://www.who.int/reproductivehealth/publications/post-violence-care-in-health-facilities/en/ (Accessed March 10, 2021)
16. World Health Organisation. Rapid Hospital Readiness Checklist: Harmonized Health Service Capacity Assessment in the Context of the COVID-19 Pandemic. Interim Guidance. Geneva: World Health Organization; 2020 https://apps.who.int/iris/handle/10665/332779
17. Hensen B, Mackworth-Young CRS, Simwinga M, et al. Remote data collection for public health research in the covid-19 era: ethical implications, challenges and opportunities. *Health Policy Plan* 2021;czaa158 https://doi.org/10.1093/heapol/czaa158
18. IMPACT. 2020. SOPs for Data Collection during COVID-19. https://www.reachresourcecentre.info/wp-content/uploads/2020/05/DataCollectionSOPCOVID-19.pdf (Accessed March 10, 2021)
19. Zulu JM, Ali J, Hallex K, et.al. Ethical challenges in research on post-abortion care with adolescents: experiences of researchers in Zambia. *Glob Bioeth* 2018; 31(1):104-119
20. World Health Organisation. Everybody business: strengthening health systems to improve health outcomes: WHO's framework for action. Geneva, World Health Organisation; 2007. https://www.who.int/healthsystems/strategy/everybodys_business.pdf?ua=1 (Accessed August 15, 2020)
21. Elo S, Kyngäs H. The qualitative content analysis process. *J Adv Nurse* 2008;62:107-115.
22. UN Women, World Health Organisation. Violence Against Women and Girls. Data Collection during COVID-19. New York, UN Women; 2020 https://www.unwomen.org/-/media/headquarters/attachments/sections/library/publications/2020/vawg-data-collection-during-covid-19 (Accessed March 10, 2021)
23. World Health Organisation. Putting Women First: Ethical and Safety Recommendations for research on Domestic Violence Against Women. Geneva, World Health Organization;2001

For peer review only - http://bmjopen.bmj.com/site/about/guidelines.xhtml

https://apps.who.int/iris/bitstream/handle/10665/65893/WHO_FCH_GWH_01.1.pdf?sequence=1 (Accessed Jan 6, 2021)

For peer review only

For peer review only - http://bmjopen.bmj.com/site/about/guidelines.xhtml

Focused group topic guide for women

Demographic information (Note: Collect data on individuals before the FGD)

- Age (in completed years)
- Education (highest level attained)
- Residence (actual, urban, rural, slum)
- COVID-19 infection history (self, close family, work mates, friends)
- Social economic status - Employment status (i.e. formal or informal employment), income earning status and possibly income ranges as suitable in the country setting.

Knowledge about COVID-19 (such as cause, signs and symptoms)

1. What is COVID-19? What do you know about COVID-19?
   PROBE: for causes, transmission, signs, symptoms, sources of information about COVID-19
2. Did you perceive yourself to be at risk for COVID-19 infection?
   PROMPT: Probe for any special considerations (pregnancy, pre-existing health conditions, others).

Care seeking and perception of risk about being infected by COVID-19

3. The decision-making process of treatment seeking during COVID-19
   PROBE: Ask who influenced the decision to seek care during COVID-19 (self, partner, other family member, etc?)
4. Perception of risk of infection during SRH care seeking.
   PROMPT: How did this affect health seeking behaviour during COVID-19?
5. The treatment-seeking process during COVID-19
   a. Were your needs for accurate information on reproductive health services fulfilled? How? Or Why not? PROBE: information sources: brochures, social media, billboards, other printed media, information on service hours or location where services are offered etc.
   b. What were your needs for reproductive health services? Were these needs fulfilled? **PROBE**: Contraception? Abortion care? STI?
   c. Did you encounter any barriers in your bid to access needed reproductive health services? PROBES: transportation, behaviour of other persons, etc.

   PROMPT: Interviewer should probe for other context specific intermediaries to seeking care. E.g. paperwork for insurance
6. Do you think that reproductive services were readily available to clients who needed them? Specifically, the availability of the reproductive health medicine and medical supplies needed by clients based on the following:
   a. Availability of staff, medicines and supplies for SRH during COVID-19
   b. Schedule of treatments/appointments during COVID-19
      PROMPT: Probe for changes in the delivery of SRH services (specifically for family planning, abortion, post-abortion care and STI care) i.e. appointment-based system introduced.
   c. Interaction between the service provider and the patient was during COVID-19. PROBE: Interactions with different cadres of health workers. Nurses? Midwives? Doctors? Records? OPD staff? Etc.
   d. Was the interaction between service providers and staff described above different from pre-COVID-19 time? How?

7. What were some of the safety measures ensured by the health facility to protect clients from COVID-19 during services
8. Do you think that the COVID-19 had any impact on client protection and privacy during services?
   PROBE: What was done (or could have been) differently?

Post-pandemic recovery of the facilities to provide services in comparison to the pandemic period.

GENERAL COMMENTS: Based on the country situation, the probes for the questions above can be extended to cover the post-pandemic period if applicable.

For peer review only

Focused group topic guide for men

Demographic information

- Age (in completed years)
- Education (highest level attained)
- Residence (actual, urban, rural, slum)
- COVID-19 infection history (self, close family, work mates, friends)
- Social economic status: Employment status (i.e. formal or informal employment), income earning status and possibly income ranges as suitable in the country setting.

Knowledge about COVID-19 (such as cause, signs and symptoms)

1. What is COVID-19? What do you know about COVID-19?
   PROBE: for causes, transmission, signs, symptoms, sources of information about COVID-19
2. Did you perceive yourself to be at risk for COVID-19 infection?
   PROMPT: Probe for any special considerations (pregnancy, pre-existing health conditions, others).

Care seeking and perception of risk about being infected by COVID-19

1. What role did you play in the decision-making process of treatment seeking during COVID-19?
   PROBE: final decision maker (man only, joint, woman only, other); stage of involvement (decision stage or care seeking stage).
2. The role of men in the treatment-seeking process during COVID-19
   a. What role did you play in seeking for accurate information on reproductive health services? Were your needs fulfilled? How? Or Why not?
      PROBE: information sources: brochures, social media, billboards, other printed media, etc. The role of men in looking for sexual reproductive health services (including contraception and abortion care) and whether the needs were fulfilled
   b. What role did you play in looking for reproductive health services? Were your needs for reproductive health services fulfilled?
      PROBE: Contraception? Abortion care? STI?
   c. Did you encounter any barriers in your bid to access needed reproductive health services? PROBES: transportation, behaviour of other persons, entry restrictions for men, etc.
      NOTE: Interviewer should probe for other context specific intermediaries to seeking care. E.g. paperwork for insurance
3. Do you think that reproductive services were readily available to clients who needed them? The availability of the reproductive health medicine and medical supplies needed by clients?
   a. The schedule of treatments/appointments during COVID-19?
4. How was the interaction between the service provider and you as the male partner during COVID-19?
   PROBE: Interactions with different cadres of health workers. Nurses? Midwives? Doctors? Records? OPD staff? Etc
5. Was the interaction between service providers and staff described above different from pre-COVID-19 time? How?

For peer review only

6. What were some of the safety measures ensured by the health facility to protect clients from COVID-19 during services?
   PROBE: Were male partners stopped from entering consulting and treatment rooms due to COVID-19?
7. Do you think that the COVID-19 had any impact on client protection and privacy during services?
8. **PROBE**: What was done (or could have been) differently? E.g. barriers in seeking SRH services during COVID

Post-pandemic recovery of the facilities to provide services in comparison to the pandemic period.

COMMENT: Based on the country situation, the probes for the questions above can be extended to cover the post-pandemic period if applicable.

For peer review only

In-depth Interview topic guide for women

1. Demographic information
    - Age
    - Ethnicity
    - Marital status
    - Socio-economic status
    - Occupation
    - Education status
    - Religion
    - Place of residence and housing condition
    - Obstetric characteristics (no. of pregnancies, parity)

2. Knowledge about COVID-19
    - Cause (sexual transmission)
        - How do you think COVID-19 virus is transmitted?
        - Do you think it can be transmitted sexually?
    - Signs and symptoms of COVID-19
        - What are the sign and symptoms of COVID-19?
        - If yes, can you mention some that you know?
    - Care-seeking for COVID-19 infection
        - Do you know what to do/who to contact/ where to go if you experience COVID-19 signs and systems?
        - If yes, can you explain?
    - Sources of information and trusted communication channels (v. rumors, misinformation) Experience of COVID-19 (direct / indirect)
        - Where did you get information about COVID-19?
        - Was the information you obtained accurate?
        - Can you give me an example of accurate information you obtained?
        - Were there any rumors and/or misinformation about COVID-19?
        - How did you know that this information was just rumors or misinformation?
        - Have you fund sources of information that you can trust?
        - How did you know which information sources to trust?
        - Have you been infected by COVID-19?
        - Do you know of anyone who has been infected? If yes, was it a close friend or family member?
    - Perception of risk about being infected by COVID-19
        - Do you think you are at risk of being infected by the COVID-19 virus?
        - If no, can you tell me why not?
        - Do you think you can be infected whilst seeking care?
    - Ways to prevent being infected by COVID-19 (probe at individual, household, community levels)
        - Do you know how to prevent infection from COVID-19?
        - What can individuals do? What can households do? What can communities do?
    - Responsibility to prevent COVID-19 (probe at individual, household, community levels, authorities)
        - Who is responsible for the prevention of COVID-19 infection? Do you think this

For peer review only

responsibility was fulfilled by individuals, households, communities and authorities? How?

- Religious / socio-cultural practices and influencers
  - Are there any religious / socio-cultural practices that are used to prevent COVID-19 infection?
  - Does participation in any religious / socio-cultural practices make you practice prevention of COVID-19 differently from what is promoted by health professionals?

3. Sexual and Reproductive Health
   - Decision-making and agency to act
     - Who makes the decisions related to your sexual and reproductive health needs (self, husband/partner or family member)?
     - Who is usually responsible for carrying out the decision?
   - Issues of access / utilization
     - Did you need to seek SRH services during COVID-19?
     - If yes, were you able to access the services? How did you access the services?
     - If no, what were the barriers? What did you do?
   - Challenges faced
     - Are there any other challenges you faced in seeking SRH services during COVID-19? What are they and how did you address/manage the challenges?

4. Contraceptive practices
   - Changes in practice (if any) due to COVID-19
     - Which method of contraception do you normally use?
     - Did you have to change your normal method of contraception during COVID-19? If yes, which method and what influenced the change?
     - If no, why didn't you change from your normal practice?
   - Is pregnancy being avoided / delayed due to COVID-19? If yes, why?
   - Access to / utilization of different methods of contraception (barriers / drivers)
   - Which methods of contraception were accessible to you during COVID-19?
   - Was there a difference in the methods of contraception accessible to you compared to before COVID-19?
   - What was causing the difference?
   - Challenges in access / utilization
   - Are there any other challenges you faced in accessing/utilizing contraception services during COVID-19?
   - If yes, what were these challenges and how did you cope with them?

5. Induced abortion
   - Perceptions about abortion at normal times
   - The role abortion plays in women's lives
     - Why do you think women seek abortion services?
     - Do you think abortion is an essential service for women? If yes, in what way?
   - Fear of stigma
     - How does family, community and society view women who seek or have sought abortion services?

  - Does this stigma affect your decision whether to have an abortion?
  - Does this stigma affect how you will seek abortion services? If yes, how?
  - What do you think are the effects of this stigma on women who need abortion services?
- Inequities in access to safe abortion
  - Do you think safe abortion services are equally accessible to all women who need them?
- Perceptions about abortion during COVID-19
  - Would you choose to seek the abortion services during COVID-19, if you need one?
  - If no, why not? If yes, where and how would you access the service?
- Access to different methods of abortion (barriers / drivers)
  - Did you seek abortion services during COVID-19? If yes, which abortion methods were available for you (medical abortion or surgical abortion)?
  - What pushed you to have an abortion? If you were given a choice of methods, which one did you choose and why?
  - Did you face any barriers in seeking and accessing abortion services during COVID-19?
- Challenges in accessing / utilizing safe abortion services (inequities in access)
  - Are there any other challenges you faced in accessing/utilizing safe abortion services? If yes, what were the challenges and how did you address them?

- 9. Violence against women (*Interviewer should use the term that is used locally to describe VAW)*

- Formal and informal systems of support, and coping strategies for VAW
  - Have you or any woman you know experienced any form of VAW?
  - What are some of the systems of support available for women who have experienced VAW?
  - Do you know where and how to access this support?
  - What are some of the coping strategies women who have experience such violence use?
- Perceptions of safety in home and in community safety/increased risk during COVID-19
  - Do you think are safer or at increased risk of experiencing VAW at home or in the community during COVID-19?
  - What has made it safer/riskier for women during COVID-19?
- Women's expectations of services for gender-based violence
  - If you or any other woman experiences GBV during COVID-19, do you expect the care and support services to be available?
  - Do you know where and how to access these services?
  - Do you expect any barriers to accessing these services during COVID-19? If yes, what kind of barriers?
- What are additional stress factors (e.g. school-age children at home, older parents, loss of job/income, partner loss of job, etc.) that increase the likelihood of VAW?
- How does stress affect frequency and severity of violence, particularly during COVID-19?

For peer review only

6. Sexually transmitted infections *(interviewer should use the locally known terms for STIs and the specific diseases)*
    - Perceptions about STI
        - Is anybody at risk of getting a STI? Why or why not?
        - How are people who are known to have a STI perceived in the community?
    - Experience of STI during COVID-19
        - Have you required care and treatment for a STI during COVID-19? If yes, where and how did you seek this service?
        - Were there any barriers to accessing this service? If yes, which ones and what did you do?
        - If no, would you seek care and treatment if required during COVID-19?
        - If no, why not? If yes, where and how would you access this service?

7. Psycho-social
    - Psychosocial issues associated with being infected with COVID-19 (probe: more significant for women/men, more significant for pregnant women)
        - Did you experience any stress, anxiety or depression during COVID-19? If yes, at what level (mild, moderate, severe)?
        - What do you think led to the psychosocial issues you experienced? Was it due to being infected or other related issues or both?
        - Did your partner also experience any psychosocial issues during COVID-19?
        - Who was most affected, yourself or your partner?

8. Final questions
    - On-going concerns about COVID-19 and its possible impact
        - Are there any other concerns about COVID-19 and its impact on you, your family or in general that you wish to express?
    - Needs related to accurate information (probe further information needs – NB consider how to provide information if necessary)
        - Was accurate information readily available and accessible during COVID-19?
        - Was it easy to understand?
        - Was the information that was available adequate?
        - Was there additional information you would have liked to obtain but was missing?
        - Are there better ways of making accurate information accessible to you and others?
    - Needs related to sexual reproductive health services (probe further SRH needs – NB consider how to support/provide if necessary)
    - Do you think SRH services were responsive to your needs during COVID-19?
    - If no, which of your needs were not met?
    - Which SRH services are most important for you, that should be available even in health emergencies like COVID-19?
    - How do you think provision of SRH services can be supported during other health emergencies like COVID-19?

For peer review only

Additional themes will be added as appropriate for each target group and questions revised accordingly (with attention to first person / third person views), and across the three phases of the study.

For peer review only

In-depth Interview topic guide for men

1. Demographic information
   - Age
   - Ethnicity
   - Marital status
   - Socio-economic status
   - Occupation
   - Education status
   - Religion
   - Place of residence and housing condition

2. Knowledge about COVID-19
   - Cause (sexual transmission)
     - How do you think COVID-19 virus is transmitted?
     - Do you think it can be transmitted sexually?
   - Signs and symptoms of COVID-19
     - What are the sign and symptoms of COVID-19?
     - If yes, can you mention some that you know?
   - Care-seeking for COVID-19 infection
     - Do you know what to do/who to contact/ where to go if you experience COVID-19 signs and systems?
     - If yes, can you explain?
   - Sources of information and trusted communication channels (v. rumors, misinformation) Experience of COVID-19 (direct / indirect)
     - Where did you get information about COVID-19?
     - Was the information you obtained accurate?
     - Can you give me an example of accurate information you obtained?
     - Were there any rumors and/or misinformation about COVID-19?
     - How did you know that this information was just rumors or misinformation?
     - Have you fund sources of information that you can trust?
     - How did you know which information sources to trust?
     - Have you been infected by COVID-19?
     - Do you know of anyone who has been infected? If yes, was it a close friend or family member?
   - Perception of risk about being infected by COVID-19
     - Do you think you are at risk of being infected by the COVID-19 virus?
     - If no, can you tell me why not?
     - Do you think you can be infected whilst seeking care?
   - Ways to prevent being infected by COVID-19 (probe at individual, household, community levels)
     - Do you know how to prevent infection from COVID-19?
     - What can individuals do? What can households do? What can communities do?
   - Responsibility to prevent COVID-19 (probe at individual, household, community levels, authorities)
     - Who is responsible for the prevention of COVID-19 infection? Do you think this responsibility was fulfilled by individuals, households, communities and

For peer review only

authorities? How?

- Religious / socio-cultural practices and influencers
  - Are there any religious / socio-cultural practices that are used to prevent COVID-19 infection?
  - Does participation in any religious / socio-cultural practices make you practice prevention of COVID-19 differently from what is promoted by health professionals?

3. Sexual and Reproductive Health
   - Decision-making and agency to act
     - Who makes the decisions related to the sexual and reproductive health needs of your partner (you, partner or family member)?
     - Who is usually responsible for carrying out the decision?
   - Issues of access / utilization
     - Did your partner need to seek SRH services during COVID-19?
     - If yes, was your partner able to access the services? How did your partner access the services?
     - If no, what were the barriers? What did you and/or your partner do?
     - What role did you play in ensuring your partner sought and/or accessed the needed SRH services?
   - Challenges faced
     - Are there any other challenges your partner faced in seeking SRH services during COVID-19? What are they and how did you and/or your partner address/manage the challenges?

4. Contraceptive practices
   - Changes in practice (if any) due to COVID-19
     - Which method of contraception do you and/or your partner normally use?
     - Did you have to change your normal method of contraception during COVID-19? If yes, which method and what influenced the change?
     - If no, why didn't you change from your normal practice?
   - Did your partner avoid / delay pregnancy due to COVID-19? If yes, why?
   - Access to / utilization of different methods of contraception (barriers / drivers)
   - Which methods of contraception were accessible to you and your partner during COVID-19?
   - Was there a difference in the methods of contraception accessible to you compared to before COVID-19?
   - What was causing the difference?
   - Challenges in access / utilization
   - Are there any other challenges you and your partner faced in accessing/utilizing contraception services during COVID-19?
   - If yes, what were these challenges and how did you cope with them?
   - What role did you play in ensuring your partner sought and/or accessed contraception services?

5. Induced abortion

For peer review only

- Perceptions about abortion at normal times
- The role abortion plays in women's lives
    - Why do you think women seek abortion services?
    - Do you think abortion is an essential service for women? If yes, in what way?
- Fear of stigma
    - How does family, community and society view women who seek or have sought abortion services?
    - Does this stigma affect your decision whether to have an abortion?
    - Does this stigma affect how you will seek abortion services? If yes, how?
    - What do you think are the effects of this stigma on women who need abortion services?
- Inequities in access to safe abortion
    - Do you think safe abortion services are equally accessible to all women who need them?

- Perceptions about abortion during COVID-19
    - Would you choose to seek the abortion services during COVID-19, if your partner needed one?
    - If no, why not? If yes, where and how would you access the service?
- Access to different methods of abortion (barriers / drivers)
    - Did your partner seek abortion services during COVID-19? If yes, which abortion methods were available (medical abortion or surgical abortion)?
    - What pushed you and your partner to have an abortion? If you were given a choice of methods, which one did you choose and why?
    - Were there any barriers in seeking and accessing abortion services during COVID-19?
- Challenges in accessing / utilizing safe abortion services (inequities in access)
    - Are there any other challenges you faced in accessing/utilizing safe abortion services? If yes, what were the challenges and how did you address them?
- What was your role in ensuring your partner accessed the abortion service?

6. Violence against women (*Interviewer should use the term that is used locally to describe VAW)*
    - Formal and informal systems of support, and coping strategies for VAW
        - Has your partner or any woman you know experienced any form of VAW?
        - What are some of the systems of support available for women who have experienced VAW?
        - Do you know where and how to access this support?
        - What are some of the coping strategies that women who have experienced such violence use?
    - Perceptions of safety in home and in community safety/increased risk during COVID-19
        - Do you think women are safer or at increased risk of experiencing VAW at home or in the community during COVID-19?
        - What has made it safer/riskier for women during COVID-19?
    - Women's expectations of services for gender-based violence

- If your partner experiences GBV during COVID-19, do you expect the care and support services to be available?
- Do you know where and how to access these services?
- Do you expect any barriers to accessing these services during COVID-19? If yes, what kind of barriers?
- What do you perceive as your role in ensuring your partner get the care and support needed?

• What are additional stress factors (e.g. school-age children at home, older parents, loss of job/income, partner loss of job, etc.) that increase the likelihood of VAW?
• How does stress affect frequency and severity of violence, particularly during COVID-19?

6. Sexually transmitted infections *(interviewer should use the locally known terms for STIs and the specific diseases)*
   • Perceptions about STI
     - Is anybody at risk of getting a STI? Why or why not?
     - How are people who are known to have a STI perceived in the community?
   • Experience of STI during COVID-19
     - Has your partner required care and treatment for a STI during COVID-19? If yes, where and how did you seek this service?
     - Were there any barriers to accessing this service? If yes, which ones and what did you do?
     - If no, would you seek care and treatment if required during COVID-19?
     - If no, why not? If yes, where and how would you access this service?
     - What was your role in ensuring your partner accessed the care and treatment?

7. Psycho-social
   • Psychosocial issues associated with being infected with COVID-19 (probe: more significant for women/men, more significant for pregnant women)
     - Did you experience any stress, anxiety or depression during COVID-19? If yes, at what level (mild, moderate, severe)?
     - What do you think led to the psychosocial issues you experienced? Was it due to being infected or other related issues or both?
     - Did your partner also experience any psychosocial issues during COVID-19?
     - Who was most affected, yourself or your partner?

8. Final questions
   • On-going concerns about COVID-19 and its possible impact
     - Are there any other concerns about COVID-19 and its impact on you, your family or in general that you wish to express?
   • Needs related to accurate information (probe further information needs – NB consider how to provide information if necessary)
     - Was accurate information readily available and accessible during COVID-19?
     - Was it easy to understand?
     - Was the information that was available adequate?
     - Was there additional information you would have liked to obtain but was

missing?
- Are there better ways of making accurate information accessible to you and others?

- Needs related to sexual reproductive health services (probe further SRH needs – NB consider how to support/provide if necessary)
- Do you think SRH services were responsive to your needs during COVID-19?
- If no, which of your needs were not met?
- Which SRH services are most important for you, that should be available even in health emergencies like COVID-19?
- How do you think provision of SRH services can be supported during other health emergencies like COVID-19?

Additional themes will be added as appropriate for each target group and questions revised accordingly (with attention to first person / third person views), and across the three phases of the study.

For peer review only

In-depth Interview topic guide for SRH healthcare providers

Demographic information

- Age
- Ethnicity
- Marital status
- Socio-economic status
- Occupation
- Education status
- Religion

Health facility service availability and readiness

Health service delivery

1. Mobilization of resources for the diagnosis and the treatment of COVID-19 patients by the *(insert Country name*) government happened during COVID-19. Did the mobilization involve your facility? How was the facility involved? (Probe community, regional or national level involvement)

    ***COMMENT:** Indicate if facility was a designated COVID-19 treatment Centre*
2. What was the emergency response plan of your facility during the outbreak?
3. How did the mobilization of resources towards COVID-19 patients impact the service delivery in your facility, including the impact on human resources, space, medical supplies, etc.?
4. Did the mobilization of resources negatively or positively impact service delivery in your facility? Elaborate
5. Was there a set of essential Sexual & Reproductive Health services outlined to be maintained during the lockdown phase of the COVID-19 pandemic?

    PROMPT: Ask for each of the following services – family planning, abortion, post-abortion care, care and support for GBV and STI prevention and treatment
6. Were there disruptions to any of the SRH essential services outlined? What was the nature (services suspended, limited or shifted) of disruptions observed for each of these essential services (if any)?

    PROBES: for causes (lack of personnel, equipment, medicines or reduced demand, physical space), changes in usual service provision (introduction of appointment-based systems, telemedicine, shifting of usual location) and level of disruption (partial/ complete/not disrupted)
7. Do you think the delivery of SRH services during COVID-19 adequately responded to the needs of the clients at that time?

    PROBE: in what way?
8. What has the recovery process been like and what was the turning point?

    NOTE: Only for applicable countries.

    PROBE for coping mechanisms or approaches to ensuring SRH service delivery.

Health workforce （Human resources）

9. How did COVID-19 affect the health status of health workforce, such as doctors, nurses, ambulance drivers and hospital managers etc.? What measures were taken to protect them from the effects of the epidemic?

For peer review only

PROBE: for the provision of PPE, type of PPE, adequacy of supply and frequency of supply.

10. Was there any form of training on COVID-19? PROMPT: Probe for details of the training if provided – What areas of COVID-19? What form did this training take? Who received this training?
11. Were there employees infected? If someone was infected, how did the infection happen? What was the impact on service delivery due to the infection of SRH workers? How did it influence the service delivery?

COMMENT*: In facilities where SRH staff also provide general care in health facility, ask for the impact of an infected clinical staff in the facility even if not SRH-specific*

12. While knowing that they were at higher risk of contracting the virus, did female workers still have to fulfil the obligations such as caring for elders and immediate family (such as children or young dependents)? Is there any other conflict or challenge they faced during COVID-19? Note: Probe for any challenge worth considering in current setting.

Health information system

13. How does the reporting system for infectious diseases run?
PROMPT: Probe for reporting system for COVID-19.
14. How did this system influence the availability of services in SRH facilities?
15. How did you keep information about the COVID-19 and SRH services flowing during the outbreak with your upper-level medical institutions and potential clients in your community?
PROBE: how were changes in the delivery of SRH services communicated to clients? How was important COVID-19 information communicated to clients?

Access to essential medicines

16. Was the access to essential medicines, including regular medicines such as birth control pills, contraceptive methods impacted because of the lockdown/shutdown of the city during the COVID-19?

COMMENT*: Shutdown can be replaced with lockdown/restrictions depending on what the COVID-19 response was in country using this guide*

PROBE: ask about medicines for all the study focus SRH services

17. How has the outbreak affected the access to essential medicines in SRH for the patients and the general population?

PROBE: for each- contraceptive methods, abortion, post abortion care, STI prevention and treatment and GBV care and support

COMMENT: Probe separately for routine medication (such as antibiotics, analgesics etc where applicable)

Health systems financing

18. How did the COVID-19 epidemic affect the availability of funds at your facility?
PROMPT: Inquire about effect on the financing and maintenance of essential SRH services and possible disruptions due to the pandemic, availability of additional government funding allocated to assuring essential health services.

For peer review only

> ***COMMENT:*** *This section should be informed by the existing health financing policy (i.e. National health insurance, existing SRH related exemptions and waiver systems) or COVID-19 specific directives on financing.*

Leadership and governance

19. What are your perceptions on the roles and responsibilities of providing SRH services during outbreaks like COVID-19?
    PROBE: where does the primary responsibility to ensure continuity of services lie? Who are the enablers, implementers?
20. How did your facility respond to the emergency and manage to ensure the SRH-related service delivery in your facility?
    PROMPT: Probe for country-specific plan, policies for maintaining SRH service provision during the pandemic.
    PROBE: separately for, family planning, STI and abortion services, services prioritized by the facility during the pandemic.

Health service provider perceptions on the delivery of specific SRH services in the context of COVID-19

A. Gender based Violence

Perceptions of changes in the nature and magnitude of violence against women, and especially domestic violence during COVID-19

- Have you noticed any change in the number of women coming into the health facility reporting gender-based violence since the COVID-19 crisis began?
  - Follow up: Increase or decrease?
  - Follow up: Have the women coming in who report experiencing violence mostly been returning patients, or mostly been new patients?
  - Follow up: Have you noticed an increase in the frequency of abuse that women are experiencing since movement restrictions were put in place?
  - If possible, specify types of violence (physical, sexual, psychological by a partner), non-partner sexual abuse, other

- Do you think that the violence women have been experiencing has become more severe during the crisis, for example has there been an increase in injuries? Violence during pregnancy?
  - Follow up: Have the types of violence that women are reporting changed? How so?

Nature of service provision during COVID-19, whether services for VAW are being changed or adapted and if safety considerations are adequately addressed?

- Since the start of COVID-19, has your health facility changed or adapted services for survivors ‘of violence?
  - Probe: (if not mentioned) In some health centers, providers have maintained ‘case management’ using video calls, or text messages. Did your health center make use of any new technologies to maintain services? If so, did you develop a protocol to ensure safety of survivors?

For peer review only

Access to /use of health services for VAW and related challenges

- Some health centers have had to deal with shortages of personal protective equipment and medications that have impacted their ability to offer services during the COVID-19 crisis. Have there been any new barriers to delivering services to survivors at your health facility?
    - What are these barriers and what approaches are being used to overcome these barriers?

- In a lot of places, external referral systems have also been impacted by the crisis, such as lower numbers of available spaces in shelters due to the need for physical distancing, or NGOs that have been temporarily shut down. To your knowledge, have any changes have been made to your external referral systems? What are these?
    - Follow up: Are any of the services to which you typically refer survivors available in a more limited capacity, or no longer available due to the crisis?

Attitudes towards provision of SRH services in the context of COVID-19

- Do you think it is essential to provide services for VAW during COVID-19? Why do you think so?

B. <u>Induced abortion</u>

Perceptions of changes in the demand for abortion during COVID-19.

- Have you noticed any change in the number of women seeking abortion services at your facility during COVID-19?
    - Follow up: Increase or decrease?
    - Follow-up: any changes in the abortion methods requested?
    - What do you think were the factors driving this change?

Delivery of safe abortion services during COVID-19?

- Where and how can people seek contraceptive services during COVID-19?
    - How many methods of abortion were available in your health facility during COVID-19? Which method of abortion did you suggest during COVID-19? Why?
    - What challenges did you meet in providing safe abortion services during COVID-19? What approaches did you use to overcome these challenges?

Attitudes towards provision of SRH services in the context of COVID-19

- Do you think it is essential to provide safe abortion services during COVID-19? Why do you think so?

C. <u>Contraceptive services</u>

Perceptions of changes in the demand for contraception services during COVID-19.

- Have you noticed any change in the number of women seeking contraception services at your facility during COVID-19?
    - Follow up: Increase or decrease?
    - Follow-up: any changes in the contraception methods requested?
    - What do you think were the factor driving this change?

Delivery of contraception services during COVID-19?

- Where and how can people seek contraceptive services during COVID-19?

For peer review only

- How many kinds of contraceptive methods were available in your health facility during COVID-19?
- What are the new/ altered ways by which contraceptive services are provided as a response to COVID-19?
- What challenges did you meet in providing contraceptive services during COVID-19? What approaches did you use to overcome these challenges?

Attitudes towards provision of SRH services in the context of COVID-19

- Do you think it is essential to provide contraception services during COVID-19? Why do you think so?

D. <u>Prevention and treatment for sexually transmitted infections</u>

Perceptions of changes in the demand for care and treatment for STI during COVID-19.

- Have you noticed any change in the number of women seeking care and treatment for STI at your facility during COVID-19?
  - Follow up: Increase or decrease?
  - What do you think were the factor driving this change?

Availability of STI care and treatment during COVID-19

- Where and how can people seek care and treatment for STI during COVID-19?
  - What are the new/ altered ways by which care and treatment for STI services are provided as a response to COVID-19?
  - Were all the medicines and supplies for providing the care and treatment available during COVID-19?
  - What challenges did you meet in providing care and treatment for STI services during COVID-19? What approaches did you use to overcome these challenges?

Attitudes towards provision of SRH services in the context of COVID-19

- Do you think it is essential to provide prevention and treatment for STIs during COVID-19? Why do you think so?

3. Perceived psychosocial effects of COVID-19
   - Did you experience stress/anxiety/depression during COVID-19?
   - How would rate your psychosocial health?
   - What role did COVID-19 play in influencing your psychosocial health?
   - In what way?
   - What do you think is the psychosocial impact of COVID-19 on people?
   - Do you think the impact was the same for men and women? If yes, why?
   - If no, in what ways is the psychosocial impact different?
   - What do you think is the psychosocial impact of COVID-19 on families?
   - What do you think is the psychosocial impact of COVID-19 on communities?

4. Final questions
   - On-going concerns about COVID-19 and its possible impact
     - Are there any other concerns about COVID-19 and its impact on you, your family or in general that you wish to express?
   - Needs related to accurate information (probe further information needs – NB consider how to provide information if necessary)

For peer review only

- Was accurate information readily available and accessible during COVID-19?
- Was it easy to understand?
- Was the information that was available adequate?
- Was there additional information you would have liked to obtain but was missing?
- Are there better ways of making accurate information accessible to you and others?

- Needs related to sexual reproductive health services (probe further SRH needs – NB consider how to support/provide if necessary)
- Do you think SRH services were responsive to the needs of patients during COVID-19?
- If no, which of needs were not met?
- Which SRH services are most important, that should be available even in health emergencies like COVID-19?
- How do you think provision of SRH services can be supported during other health emergencies like COVID-19? E.g. barriers in seeking SRH services during COVID

Additional themes will be added as appropriate for each target group and questions revised accordingly (with attention to first person / third person views), and across the three phases of the study.

For peer review only
